# The effect of balance and gait training on specific balance abilities of survivors with stroke: a systematic review and network meta-analysis

**DOI:** 10.3389/fneur.2023.1234017

**Published:** 2023-11-02

**Authors:** Meng Zhang, Zhide Liang, Yali Li, Jun Meng, Xu Jiang, Bichan Xu, Haojie Li, Tao Liu

**Affiliations:** ^1^Postgraduate Department, Xi’an Physical Education University, Xi’an, China; ^2^School of Physical Education, Qingdao University, Qingdao, China; ^3^School of Physical Education, Gunagxi Minzu Normal University, Chongzuo, China; ^4^School of Exercise and Health Sciences, Xi’an Physical Education University, Xi’an, China

**Keywords:** stroke, balance, gait, rehabilitation, network meta-analysis

## Abstract

**Background:**

Stroke, which is a common clinical cerebrovascular disease, causes approximately 83% of survivors to suffer from balance impairments. Balance and gait training (BGT) is widely used to restore balance in patients with stroke. However, its wide variety presents clinicians with a dilemma when selecting interventions. This study aimed to compare and rank BGT interventions by quantifying information based on randomized controlled trials (RCTs).

**Methods:**

We conducted a network meta-analysis (NMA) of non-gait-trained controls and head-to-head RCTs and compared the effects of 12 BGT interventions. A total of nine literature databases, including Medline, Embase, Cochrane Library, Web of Science, Scopus, SPORTDiscus, ClinicalTrials.gov, CNKI, and Chinese biomedical literature databases, were searched from their database inception to August 2023. Two authors independently selected studies and extracted data. The difference in outcomes, which were expressed as standardized mean differences and confidence intervals (CIs) of 95%, were explored in this meta-analysis.

**Results:**

A total of 66 studies with 1,933 participants were included. Effect size estimates showed that not all BGT interventions were more effective than controls, with treadmill training as the least effective for balance test batteries (SMD = −0.41, 95% CI [−1.09, 0.27]) and proactive balance (SMD = −0.50, 95% CI [−1.14, 0.14]). Body-weight-supported treadmill training with external stimulation was most effective for proactive balance and dynamic steady-state balance (SMD = 1.57, 95% CI [−0.03, 3.16]); SMD = 1.18, 95% CI [0.67, 1.68]. Virtual reality gait training (SMD = 1.37, 95% CI [0.62, 2.11]) had the best effect on improving balance test batteries, while dual-task BGT (SMD = 1.64, 95% CI [0.50, 2.78]) had the best effect on static steady-state balance. After analyses for possible impact covariates, the findings through the outcomes did not change substantially. Confidence in the evidence was generally low or very low.

**Conclusion:**

This NMA suggested that virtual reality gait training was the most effective BGT modality for improving balance test batteries. Body-weight support treadmill training with external stimulation was the most effective for improving active and dynamic balance. In addition, dual-task BGT was the best choice for improving static balance. However, balance is a multidimensional concept, and patients’ different needs should be considered when selecting BGT.

**Systematic review registration:**

https://www.crd.york.ac.uk/prospero/display_record.php?ID=CRD42022316057, ID: CRD42022316057.

## Introduction

Stroke is a common clinical cerebrovascular disease with high morbidity, mortality, and disability, and the second most common cause of death in the world (1–3). In 2019, approximately 100 million people suffered from stroke worldwide (4), with its global incidence increasing by 85% and mortality by 43% from 1990 to 2019, whose mortality rate in low- and middle-income countries is 3.6 times higher than that in high-income countries, placing a heavy burden on society and families (5).

Balance refers to the ability to hold the line of gravity within the point of support with minimal postural sway (6). Approximately 83% of stroke survivors are reported to suffer from balance disorders, one of the most common impairments for patients with stroke, which are associated with more severe physical impairments, disabilities, and a lower quality of life (7). In addition, balance disorders are strongly associated with a high rate of falls, which places a significant burden on patients with stroke and their families (7, 8). Several studies have shown that exercise training positively affects balance improvement (9–11) and that balance and gait training (BGT) is considered an essential aspect of fall prevention (12–14). Therefore, involving BGT in the balance rehabilitation program for patients with stroke has become particularly important.

Recently, more BGT interventions have been introduced to improve the balance of patients with stroke, such as dual-task gait training (15), virtual reality gait training (16), and robot-assisted gait training (17). The wide variety of BGT makes it a dilemma for clinicians to choose from available interventions. Those head-to-head intervention trials can be synthesized through traditional meta-analyses, providing some evidence. However, it is difficult to compare the efficacy of different BGT interventions, resulting in the inclusion of fewer bodies of literature (18), which does not allow for further exploration of the relative effectiveness among the various BGT interventions, while providing a ranking of priorities among different interventions. Moreover, previous meta-analyses had a high level of heterogeneity (17, 19), whose results might change with the inclusion of more kinds of literature.

Although there have been numerous studies demonstrating that BGT can be used to improve the balance of stroke survivors (17, 20, 21), they do not provide a comprehensive overview using network meta-analyses (NMAs) or compare the effect of BGT on various balance abilities. Through NMAs, these limitations are overcome by including a greater number of relevant trials while bringing together direct and indirect comparisons of all BGT interventions available (22, 23). Therefore, this network meta-analysis aimed to evaluate the effect of BGT on the balance of patients with stroke so as to examine the relative effect of various BGT interventions on the balance (balance test batteries, dynamic steady-state balance, static steady-state balance, proactive balance) of patients with stroke while further enhancing knowledge in this area. The pair meta-analyses and meta-regression analyses on control group (CON) data were also applied to examine patients’ gender and age, timing and frequency of interventions, year of publication, and the time to study entry after a stroke to predict the extent of changes in their balance ability as well as to provide referable evidence for clinicians, patients, and caregivers.

## Methods

### Study protocol and registration

The study protocols for this systematic review were registered in the PROSPERO database (CRD42022316057) and meet the Preferred Reporting Items for Systematic Reviews and Meta-Analyses (PRISMA) extended statement criteria (24) [Appendix 2 (p. 6)].

### Search strategy

A total of nine literature databases, namely, Medline, Embase, Cochrane Library, Web of Science, Scopus, SPORTDiscus, ClinicalTrials.gov, CNKI, and Chinese biomedical literature databases, were searched from their inception to August 2023, with no language restrictions. The combined Medical Subject Heading (MeSH) terms and keywords with Boolean operators were applied to search through the search strategy described in detail [Appendix 3 (p. 21)], which mainly includes the following terms: (stroke), (exercise OR training OR gait training), (randomized controlled trial, RCT), and (balance). We also performed a recursive search to identify relevant publications by manually filtering the bibliographic lists of similar reviews and large professional conferences. The results of all studies searched were initially screened by two independent reviewers (M.Z. and Zd. L.) through titles and abstracts based on the inclusion and exclusion criteria, and their full text, which met the initial screening requirements, was extracted. Two reviewers further independently screened studies that met the criteria and resolved differences through discussion with a third reviewer (T.L.), adjudicating when necessary.

### Inclusion and exclusion criteria

The following were the inclusion criteria: (a) participants should be adults affected by stroke with an age of ≥18 years (according to the clinical definition); (b) the trials included at least two types of BGT intervention to be compared, or BGT intervention and control to be compared; see Table 1 for details of intervention and control; (c) according to Shumway-Cook and Woollacott (25), balance is a highly specific task that has to be divided into different categories: static/dynamic steady-state balance (maintaining a stable position while sitting, standing, and walking), proactive balance (anticipating disturbances), and reactive balance (compensation for disturbances). Concerning these findings, our study was focused on different balance categories: (1) balance test batteries (such as the Berg Balance Scale), (2) dynamic steady-state balance (such as the 10-m gait speed test), (3) static steady-state balance (such as the center of pressure (CoP) displacements during single-leg stance), (4) proactive balance (such as the Functional Reach-Test or Timed Up and Go (TUG) Test), and (5) reactive balance (such as the CoP displacements after an unexpected perturbation). At least one of the aforementioned five types of balance should be present in the results; (d) RCTs; (e) if the study data were missing, we emailed the authors to inquire about them, and the study was disqualified if we did not hear back.

**Table 1:** Interventions and abbreviations.

Abbreviation	Intervention
CON	No balance and walking training (usual care, keep daily or wait-list)
BGT	Balance and gait training without technical assistance (such as treadmill-assisted, robot-assisted, and body-weight support-assisted)
TT	Treadmill training without body-weight support
BWS-TT	Treadmill training with body-weight support
RA-GT	Robot-assisted gait training
VR-GT	Virtual reality gait training
AQE-BGT	Aquatic balance and gait training
BGT-ECA	Balance and gait training with external stimulation (includes visual stimulation, auditory stimulation, electrical stimulation)
TT-ECA	Treadmill training with external stimulation
BWS-TT-ECA	Body-weight support treadmill training with external stimulation
RA-GT-ECA	Robot-assisted balance and gait training with external stimulation
EC-BGT	Eye closed balance and gait training (gait training without visual aids, including closed-eye gait training, backward gait training)
DT-BGT	Dual-task balance and gait training (perform both types of training, at least one of the above-mentioned balance gait trainings)

### Data extraction

Two investigators (M.Z. and Zd. L.) independently extracted data from the final studies included and entered them into a standardized data extraction spreadsheet through Excel. The following information was extracted: (1) author and year of publication; (2) relevant data on participants’ characteristics (such as sample size, age, sex, degree of stroke, and time from stroke onset to study entry); and (3) details of interventions in the treatment and CON. The two investigators independently categorized the interventions in each included study, and any discrepancy was resolved through discussion, involving a third investigator if necessary. The total duration, intensity, and frequency of interventions were also extracted; (4) all information on balance outcomes (such as the Berg Balance Scale, 10-m walking speed test, CoP displacement during single-legged stance, and TUG) was analyzed across balance types. In this systematic review, two investigators independently assessed all studies (M.Z. and Zd. L.) based on the information extracted. If there was a disagreement on including a study, a third reviewer (T.L.) was consulted.

### Risk of bias assessment

Two reviewers (M.Z. and Zd. L.) independently assessed the risk of bias in randomized controlled trials using the revised Cochrane Risk of Bias, version 2 (RoB 2) tool (26). Disagreements between the reviewers were settled by discussion, and if no consensus could be reached, a third reviewer (T.L.) made the final decision as an adjudicator (T.L.).

### GRADE assessment

The Grading of Recommendations Assessment, Development, and Evaluation (GRADE) approach was used to assess the quality of evidence for the results of different BGT rankings based on NMA, including study limitations, indirectness and transitivity, statistical heterogeneity and inconsistency, and imprecision and publication biases (27). The GRADE method is used for each pairwise comparison, whose framework has been adapted to NMA (28, 29), in which all the studies included are RCTs, and it is assumed that each study would have the highest initial quality rating and, after assessing the above factors, would be rated as having a moderate, low, or very low quality where appropriate.

### Data analysis

#### Assessment of the transitivity assumption

Transitivity is a critical underlying assumption of NMAs (30). To assess this hypothesis, we examined the distribution of possible effect modifiers by comparing intervention methods for further analyses, including baseline characteristics of participants, intervention duration, and intervention frequency (23, 31, 32).

### Network meta-analysis

Network evidence was plotted using STATA15.1 (Stata Corp LLC, College Station, TX, United States) to represent the geometric structure of different BGT. The dots represent different intervention types, whose size represents the number of studies, and the line among each intervention type represents a direct comparison among interventions. We extracted baseline and endpoint mean differences and standard deviations (SD) for relevant outcomes; if SD was not reported in the study, standard errors (SE), 95% confidence intervals, and interquartile intervals would be used for estimation (33). If a lower value represented a better study result, we would multiply the result by −1, as recommended in the Cochrane Handbook *for Systematic Reviews of Interventions* (33). If the study was a multi-arm RCT, then data would be extracted for all interventions and CONs of the study. Because different measures of outcomes were used for each type of balance, to ensure the comparability of results, the standardized mean difference (SMD) of the results of all continuous variables was used to estimate the effect. We used the “netmeta 1.5–0” package of R software (version 3.6.2, The R Foundation for Statistical Computing, Vienna, Austria) to perform a meta-analysis on the random-effects networks of a frequency-based framework (34). The heterogeneity of each network was assessed by statistics τ^2^ and I^2^, whose consistency (between direct and indirect evidence) was assessed using both global (Q statistics) and local methods (identifying inconsistent “hot spots” using the “node-split” function) (27, 35). We used the R “netmeta” package to separate indirect evidence from direct evidence (SIDE test) (35) to statistically assess global consistency (consistency across sources of evidence) (36). Inconsistencies were statistically tested and reported using z-scores and *p*-values, of which *p*-values < 0.05 were considered statistically significant (37, 38). The effect of different BGT interventions was assessed using a frequency ranking method, and the probability of ranking for each BGT was expressed as a P-score, which is a measure of the degree of certainty that one intervention is better than another, with higher *p* values representing better BGT interventions, together with an upper limit of 1 (39). To represent the results more visually, we created a heat map to summarize the ranking of the effect of all BGT on different balance abilities. A forest diagram was created to visually represent the effect of different BGTs compared to the CON.

### Meta-regression: baseline predictors of changes in balance associated with balance and gait training

After screening analyses, the results suggested that age, gender, duration of illness, year of publication, frequency of interventions, and duration were factors most likely to influence outcomes. The “gemtc” package (1.0–1) in R was applied to investigate the effect of covariates on the balance ability of the subjects. We performed meta-regression analyses using CON group data to investigate the relationship between subjects’ balance ability and their age, gender, duration of illness, year of publication, frequency of interventions, and duration of interventions (40, 41). In this analysis, if a study involved multiple subgroups, their estimates would be combined (33).

## Results

### Literature selection

**Figure 1:**
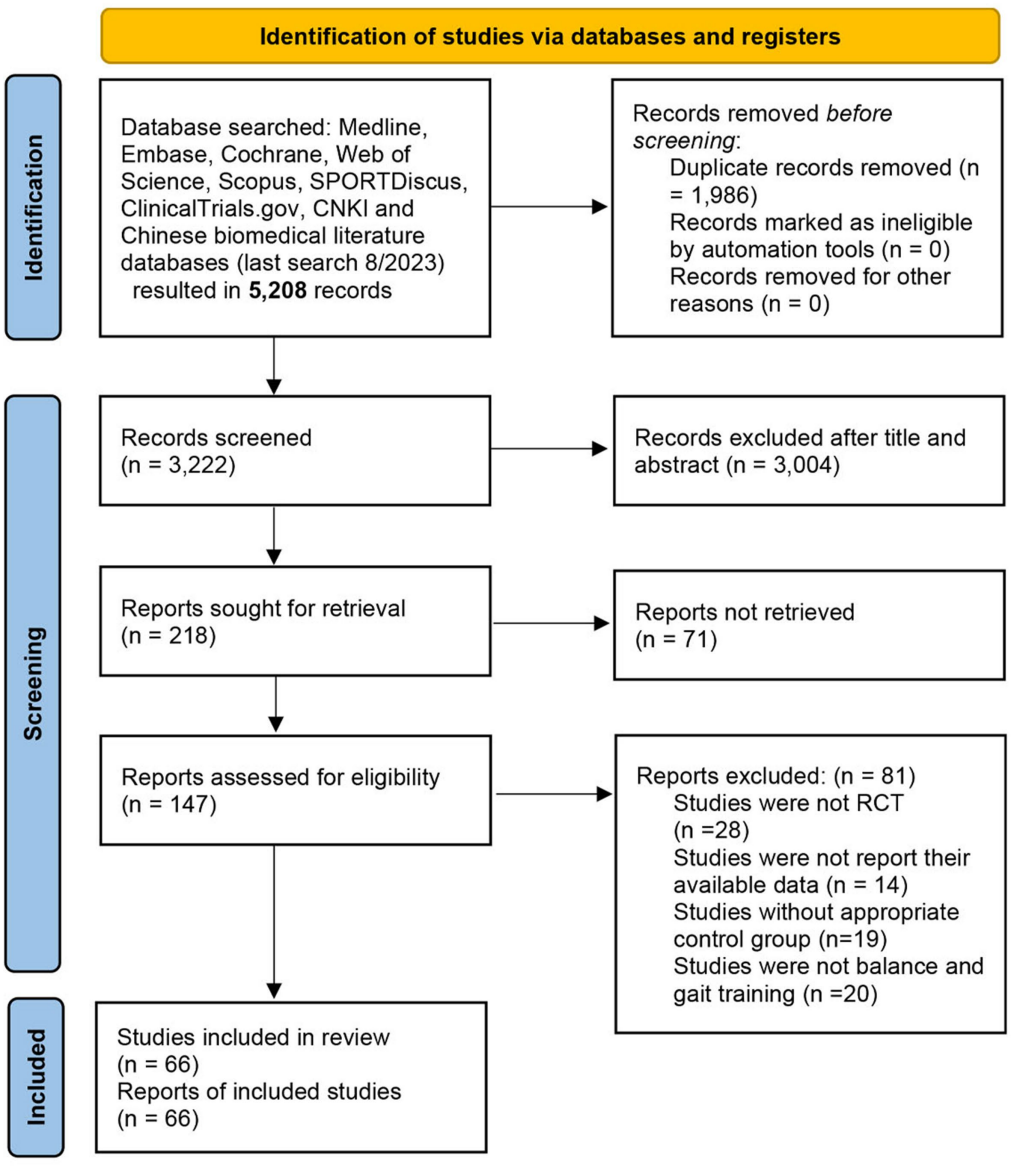
PRISMA flow diagram.

A total of 5,208 articles were obtained by searching and screening the databases, of which 1,986 studies were excluded for the first time due to duplication. We excluded another 3,004 after reviewing titles and abstracts; another 71 were excluded because no report was retrieved; and finally, 147 studies were screened for the full text. We excluded 81 studies after full-text screening for the following reasons: 28 studies were not RCTs, 14 did not have appropriate outcomes or failed to provide analyzable data, 19 did not have an appropriate control group, and 20 had an intervention type other than the BGT defined in this study. Finally, 66 studies were included in our network meta-analysis, and details of exclusions and screening are shown in Figure 1.

### Characteristics of the included studies

A total of 1,975 subjects participated in our study, with an age range of 44–74 years. The proportion of male participants was significantly higher than that of female participants (male participants: 1,236, 62.6% vs. female participants: 739, 37.4%). The study was completed mainly in Korean regions (*N* = 29, 43.9%). Patients’ average time from stroke to entry into interventions was 2 years; the average intervention period was 5.2 weeks, and the frequency of interventions ranged from 1 to 7 times per week.

Of the included studies, six studies were three-armed controlled experiments, and the remaining studies were two-armed. The results on balance test batteries were reported in 47 studies; those on dynamic and static steady-state balance were reported in 64 and 21 studies, respectively; and the results related to proactive balance were reported in the additional 28 studies. Network graphs are shown in Supplementary Figure S1. The demographic characteristics of the included studies were summarized in Appendix 4 (p. 27), and the forest plots and funnel plots of all outcomes would be presented in Appendices 7, 11 (p. 42 and p. 70).

### Results of the risk of biases

RoB 2 results showed that, for balance test batteries, dynamic steady-state balance, static steady-state balance, and proactive balance outcomes, 6.8%, 8.1%, 10%, and 3.7% of studies had a high risk; 27.3%, 30.6%, 30%, and 29.6% had some risk concerns; and 65.9%, 61.3%, 60%, and 66.7% had a low risk. Overall, we judged four balanced outcomes as having a high risk of bias. If raw, unadjusted scores of registered outcomes were reported, we considered the risk of selective reporting low. The risk of bias was not presented due to selective non-reporting or under-reporting, as this type of bias is not covered in RoB 2. The detailed process of risk assessment is shown in Supplementary Figures S2–S9 (justifications in Appendix 12).

### Network meta-analysis

#### Balance test batteries

**Figure 2:**
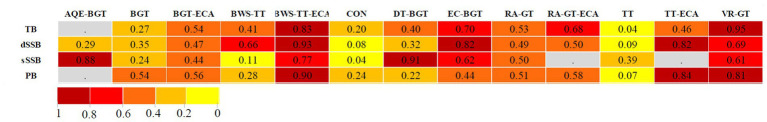
Heat map of balance and gait training interventions. A heat map of balance and gait training interventions ranked according to associated degree of alteration in balance test batteries, dynamic steady-state balance, static steady-state balance, and proactive balance numbers reflect P-scores, which rank interventions on a continuous scale from 0 to 1. A higher P-score indicates a greater increase in the balance parameter. Gray squares indicate that data were not available. AQE-BGT, aquatic balance and gait training; BGT, balance and gait training; BGT-ECA, balance and gait training with external cues; BWS-TT, body weight-supported treadmill training; BWS-TT-ECA, body weight-supported treadmill training with external cues; CON, control group; dSSB, dynamic steady-state balance; DT-BGT, dual-task gait training; EC-BGT, eyes closed gait training; PB, proactive balance; sSSB, static steady-state balance; RA-GT, robotic-assisted gait training; RA-GT-ECA, robotic-assisted gait training with external cues; TB, balance test battery; TT, treadmill gait training; TT-ECA, treadmill gait training with external cues; VR-GT, virtual reality gait training.

A total of 47 studies (1,360 participants) included the results of balance test batteries. The mixed comparisons in the league table showed that the balance test batteries of the VR-GT group improved significantly better than those of the RA-GT, DT-BGT, BWS-TT, BGT, TT, and CON groups, with a BWS-TT-ECA that was significantly better than that of the BWS-TT, BGT, TT, and CON groups. Other comparative details are shown in Table 2A. Following the ranking of the effects of the balance improvement scale, VR-GT had the best effect (P-score: 0.95) while TT had the worst (P-score: 0.04; Figure 2). The overall heterogeneity of the NMA model for balance test batteries was significant (τ^2^ = 0.29, I^2^ = 65.5%, *p* < 0.001). The Q score of global inconsistency was 7.46 (*p* = 0.9632), and no hotspot of inconsistency was found in the models analyzed for inconsistency through the point-split method in Appendix 9 (p. 47).

**Table 2A:** League table of balance test batteries.

VR-GT	.	.	0.71 (−0.66, 2.09)	.	.	.	.	.	.	.	**1.32 (0.32, 2.32)**	**1.85 (0.93, 2.78)**
0.37 (−0.87, 1.61)	EC-BGT	0.49 (−0.91, 1.89)	.	.	.	.	.	.	.	0.74 (−0.77, 2.24)	0.80 (−0.60, 2.20)	.
0.51 (−0.48, 1.50)	0.14 (−0.91, 1.19)	BWS-TT-ECA	.	.	.	.	.	.	**0.79 (0.04, 1.54)**	0.50 (−0.82, 1.82)	0.71 (−0.22, 1.64)	.
0.76 (−0.18, 1.71)	0.39 (−0.84, 1.63)	0.26 (−0.74, 1.25)	RA-GT-ECA	.	0.16 (−1.25, 1.57)	.	.	.	.	.	0.61 (−0.30, 1.53)	.
0.74 (−0.93, 2.41)	0.37 (−1.43, 2.17)	0.23 (−1.41, 1.87)	−0.02 (−1.70, 1.65)	AQE-BGT	.	.	.	.	.	.	0.59 (−0.90, 2.08)	.
**0.92 (0.11, 1.74)**	0.55 (−0.48, 1.59)	0.42 (−0.32, 1.15)	0.16 (−0.64, 0.96)	0.18 (−1.37, 1.73)	RA-GT	.	.	.	−0.36 (−1.84, 1.12)	0.59 (−0.03, 1.21)	0.19 (−0.35, 0.73)	**1.33 (0.11, 2.54)**
0.92 (−0.03, 1.87)	0.55 (−0.57, 1.67)	0.41 (−0.44, 1.26)	0.15 (−0.79, 1.10)	0.18 (−1.43, 1.78)	−0.01 (−0.68, 0.67)	BGT-ECA	.	.	.	0.46 (−0.40, 1.31)	0.32 (−0.41, 1.06)	.
1.03 (−0.17, 2.23)	0.66 (−0.85, 2.18)	0.53 (−0.80, 1.85)	0.27 (−1.07, 1.61)	0.29 (−1.60, 2.19)	0.11 (−1.08, 1.30)	0.12 (−1.18, 1.41)	TT-ECA	.	.	.	.	0.74 (−0.20, 1.68)
**1.14 (0.10, 2.17)**	0.77 (−0.55, 2.09)	0.63 (−0.45, 1.71)	0.38 (−0.77, 1.52)	0.40 (−1.36, 2.16)	0.22 (−0.73, 1.16)	0.22 (−0.85, 1.29)	0.11 (−1.14, 1.35)	DT-BGT	−0.06 (−1.38, 1.26)	.	.	0.66 (−0.28, 1.60)
**1.13 (0.26, 1.99)**	0.76 (−0.28, 1.79)	0.62 (−0.03, 1.26)	0.36 (−0.52, 1.24)	0.39 (−1.19, 1.96)	0.20 (−0.35, 0.75)	0.21 (−0.50, 0.92)	0.09 (−1.13, 1.32)	−0.01 (−0.94, 0.91)	BWS-TT	0.13 (−0.51, 0.77)	0.22 (−0.53, 0.96)	.
**1.26 (0.40, 2.12)**	0.89 (−0.11, 1.88)	**0.75 (0.05, 1.44)**	0.49 (−0.37, 1.35)	0.52 (−1.05, 2.08)	0.33 (−0.14, 0.80)	0.34 (−0.28, 0.96)	0.22 (−1.00, 1.45)	0.12 (−0.85, 1.09)	0.13 (−0.36, 0.63)	BGT	1.32 (−0.09, 2.73)	.
**1.33 (0.58, 2.09)**	0.96 (−0.04, 1.96)	**0.82 (0.15, 1.50)**	0.57 (−0.19, 1.32)	0.59 (−0.90, 2.08)	0.41 (−0.01, 0.83)	0.41 (−0.18, 1.01)	0.30 (−0.88, 1.47)	0.19 (−0.73, 1.12)	0.20 (−0.30, 0.71)	0.07 (−0.41, 0.55)	CON	−0.28 (−1.56, 1.00)
**1.77 (1.03, 2.51)**	**1.40 (0.21, 2.59)**	**1.27 (0.34, 2.19)**	**1.01 (0.05, 1.97)**	1.03 (−0.61, 2.68)	**0.85 (0.13, 1.57)**	0.86 (−0.03, 1.75)	0.74 (−0.20, 1.68)	0.63 (−0.18, 1.45)	0.65 (−0.13, 1.43)	0.52 (−0.26, 1.30)	0.44 (−0.25, 1.14)	TT

The effects of different BGT Interventions were assessed using a frequency ranking method, and the probability of ranking for each BGT was expressed as a P-score. Results of the network meta-analysis are presented in the left lower half and results from pairwise comparisons in the upper right half, if available. Comparisons between Interventions should be read from left to right and the estimate is in the cell in common between the column-defining Intervention and the row-defining Intervention. In the left lower half, standard mean differences (SMDs) higher than 0 favor the column-defining Intervention, in the upper right half SMDs higher than 0 favor the row defining Intervention. Cells in bold print indicate significant results “.” = not available.

#### Dynamic steady-state balance

The results on dynamic steady-state balance were reported in 64 studies (1,861 participants). Through a mixed comparison, we found that the dynamic balance of the BWS-TT-ECA group improved better than that of the RA-GT, BGT-ECA, BGT, DT-BGT, TT, and CON groups, and other comparative details are shown in Table 2B. In terms of the P-score ranking, BWS-TT-ECA had the best intervention effect (P-score: 0.93), while CON had the worst (P-score: 0.08; Figure 2). We found moderate overall heterogeneity in studies on dynamic balance (τ^2^ = 0.13, I^2^ = 45.2%, *p* < 0.001). The Q score of the global inconsistency assessment was 27.11 (*p* = 0.2514). However, after analyzing inconsistency using the point-split method, two hotspots of inconsistency were found and shown in Appendix 9 (p. 49), indicating a disagreement between direct and indirect evidence.

**Table 2B:** League table of dynamic steady-state balance.

BWS-TT-EC**A**	.	.	.	0.43 (−0.07, 0.93)	.	.	.	0.68 (−0.02, 1.38)	.	.	.	0.83 (−0.11, 1.76)
0.20 (−0.53, 0.92)	TT-ECA	.	.	.	.	.	0.95 (−0.24, 2.14)	.	.	.	**0.87 (0.34, 1.40)**	1.12 (−0.04, 2.28)
0.20 (−0.49, 0.90)	0.01 (−0.68, 0.69)	EC-BGT	.	0.58 (−0.54, 1.69)	.	.	.	1.25 (−0.06, 2.56)	.	.	**0.77 (0.07, 1.47)**	**0.94 (0.10, 1.78)**
0.39 (−0.25, 1.03)	0.19 (−0.45, 0.83)	0.19 (−0.45, 0.82)	VR-GT	.	0.49 (−0.58, 1.55)	−0.41 (−1.46, 0.63)	.	0.02 (−0.86, 0.90)	.	.	**1.02 (0.35, 1.69)**	**1.06 (0.28, 1.85)**
0.43 (−0.03, 0.89)	0.23 (−0.38, 0.85)	0.23 (−0.34, 0.80)	0.04 (−0.47, 0.56)	BWS-TT	.	−0.21 (−1.41, 1.00)	.	**0.61 (0.18, 1.04)**	−0.01 (−1.02, 0.99)	.	.	**0.70 (0.23, 1.18)**
0.61 (−0.26, 1.48)	0.42 (−0.48, 1.31)	0.41 (−0.47, 1.29)	0.22 (−0.55, 0.99)	0.18 (−0.60, 0.96)	RA-GT-ECA	0.07 (−1.05, 1.19)	.	.	.	.	.	0.70 (−0.34, 1.74)
**0.61 (0.07, 1.14)**	0.41 (−0.17, 1.00)	0.40 (−0.15, 0.96)	0.22 (−0.24, 0.68)	0.18 (−0.20, 0.55)	−0.00 (−0.73, 0.72)	RA-GT	.	0.11 (−0.34, 0.55)	.	.	**1.23 (0.41, 2.04)**	**0.37 (0.02, 0.72)**
**0.63 (0.06, 1.21)**	0.44 (−0.18, 1.05)	0.43 (−0.18, 1.04)	0.24 (−0.30, 0.78)	0.20 (−0.24, 0.64)	0.02 (−0.78, 0.82)	0.03 (−0.39, 0.44)	BGT-ECA	0.15 (−0.27, 0.57)	.	.	.	**0.59 (0.02, 1.16)**
**0.74 (0.25, 1.23)**	0.54 (−0.04, 1.13)	0.54 (−0.01, 1.09)	0.35 (−0.12, 0.82)	0.31 (−0.02, 0.64)	0.13 (−0.63, 0.89)	0.13 (−0.18, 0.44)	0.11 (−0.24, 0.45)	BGT	.	0.06 (−1.04, 1.16)	.	**1.37 (0.55, 2.20)**
**0.84 (0.07, 1.62)**	0.65 (−0.11, 1.40)	0.64 (−0.12, 1.40)	0.45 (−0.27, 1.18)	0.41 (−0.25, 1.07)	0.23 (−0.72, 1.19)	0.24 (−0.44, 0.91)	0.21 (−0.51, 0.93)	0.10 (−0.57, 0.77)	DT-BGT	.	0.12 (−0.59, 0.83)	.
0.91 (−0.01, 1.83)	0.71 (−0.25, 1.67)	0.70 (−0.24, 1.65)	0.52 (−0.38, 1.42)	0.48 (−0.36, 1.32)	0.29 (−0.78, 1.37)	0.30 (−0.53, 1.12)	0.27 (−0.58, 1.13)	0.17 (−0.63, 0.97)	0.06 (−0.95, 1.08)	AQE-BGT	.	0.16 (−0.96, 1.28)
**1.17 (0.57, 1.78)**	**0.98 (0.49, 1.46)**	**0.97 (0.45, 1.49)**	**0.79 (0.31, 1.26)**	**0.74 (0.28, 1.20)**	0.56 (−0.23, 1.35)	**0.57 (0.15, 0.98)**	**0.54 (0.05, 1.04)**	**0.43 (0.00, 0.87)**	0.33 (−0.27, 0.93)	0.27 (−0.61, 1.14)	TT	0.14 (−0.59, 0.86)
**1.18 (0.67, 1.68)**	**0.98 (0.42, 1.54)**	**0.97 (0.44, 1.50)**	**0.79 (0.34, 1.23)**	**0.74 (0.41, 1.08)**	0.56 (−0.17, 1.29)	**0.57 (0.29, 0.84)**	**0.54 (0.17, 0.92)**	**0.43 (0.13, 0.73)**	0.33 (−0.32, 0.99)	0.27 (−0.53, 1.07)	0.00 (−0.39, 0.40)	CON

The effects of different BGT Interventions were assessed using a frequency ranking method, and the probability of ranking for each BGT was expressed as a P-score. Results of the network meta-analysis are presented in the left lower half and results from pairwise comparisons in the upper right half, if available. Comparisons between Interventions should be read from left to right and the estimate is in the cell in common between the column-defining Intervention and the row-defining Intervention. In the left lower half, standard mean differences (SMDs) higher than 0 favor the column-defining Intervention, in the upper right half SMDs higher than 0 favor the row defining Intervention. Cells in bold print indicate significant results “.” = not available.

#### Static steady-state balance

Studies on static steady-state balance outcomes were the fewest, with a total of 21 studies involving a total of 609 patients with stroke. According to the league table of static balance, we found that the DT-BGT group was significantly better than the TT, BGT, BWS-TT, and CON groups; the AQE-BGT group was significantly better than the RA-GT, BGT, BWS-TT, and CON groups; and the other comparisons are shown in Table 2C. Additionally, no significant improvement effect of TT, BGT, or BWS-TT was found for static balance. In the overall effect ranking, DT-BGT had the best effect with a P-score of 0.91, and CON was the worst with a P-score of 0.04 (Figure 2). In the heterogeneity analysis, the overall heterogeneity was shown to be good (τ^2^ = 0.01, I^2^ = 7.3%, *p* = 0.3735). The global Q score of inconsistency was 11.25 (*p* = 0.1282). In the inconsistency test, we found no hotspots of inconsistency in Appendix 9 (p. 52), indicating a relatively good consistency of the study.

**Table 2C:** League table of static steady-state balance.

DT-BGT	.	.	.	.	.	.	**1.04 (0.24, 1.84)**	.	.	.
0.16 (−1.17, 1.50)	AQE-BGT	.	.	.	.	.	.	0.64 (−0.27, 1.55)	.	**2.27 (1.08, 3.46)**
0.44 (−0.58, 1.47)	0.28 (−0.79, 1.35)	EC-BGT	.	.	.	.	**0.78 (0.08, 1.49)**	0.43 (−0.59, 1.45)	.	.
0.73 (−0.31, 1.78)	0.57 (−0.40, 1.54)	0.29 (−0.52, 1.09)	VR-GT	.	.	0.27 (−0.53, 1.07)	0.10 (−0.65, 0.85)	.	.	**1.36 (0.24, 2.49)**
0.75 (−0.48, 1.98)	0.59 (−0.28, 1.46)	0.31 (−0.61, 1.23)	0.02 (−0.81, 0.85)	BGT-ECA	.	.	.	**0.51 (0.06, 0.96)**	.	.
0.92 (−0.28, 2.13)	0.76 (−0.09, 1.61)	0.48 (−0.43, 1.39)	0.19 (−0.59, 0.97)	0.17 (−0.49, 0.83)	BWS-TT-ECA	.	.	0.15 (−0.60, 0.89)	0.64 (0.14, 1.14)	**1.05 (0.38, 1.72)**
0.99 (−0.15, 2.13)	**0.83 (0.01, 1.65)**	0.55 (−0.30, 1.39)	0.26 (−0.36, 0.88)	0.24 (−0.43, 0.91)	0.07 (−0.52, 0.65)	RA-GT	.	0.58 (−0.27, 1.43)	.	**0.59 (0.20, 0.98)**
**1.04 (0.24, 1.84)**	0.88 (−0.19, 1.95)	0.60 (−0.04, 1.24)	0.31 (−0.36, 0.98)	0.29 (−0.65, 1.22)	0.12 (−0.79, 1.02)	0.05 (−0.76, 0.86)	TT	.	.	.
**1.26 (0.11, 2.41)**	**1.10 (0.36, 1.84)**	**0.82 (0.01, 1.62)**	0.53 (−0.17, 1.23)	**0.51 (0.06, 0.96)**	0.34 (−0.15, 0.82)	0.27 (−0.22, 0.76)	0.22 (−0.60, 1.04)	BGT	0.16 (−0.51, 0.83)	−0.08 (−0.91, 0.74)
**1.50 (0.30, 2.70)**	**1.33 (0.49, 2.17)**	**1.05 (0.16, 1.95)**	**0.76 (0.00, 1.53)**	**0.74 (0.10, 1.39)**	**0.57 (0.12, 1.02)**	0.50 (−0.06, 1.07)	0.45 (−0.44, 1.35)	0.23 (−0.23, 0.70)	BWS-TT	−0.03 (−0.67, 0.61)
**1.64 (0.50, 2.78)**	**1.48 (0.71, 2.25)**	**1.20 (0.36, 2.03)**	**0.91 (0.27, 1.55)**	**0.89 (0.26, 1.52)**	**0.72 (0.21, 1.23)**	**0.65 (0.30, 1.00)**	0.60 (−0.21, 1.41)	0.38 (−0.06, 0.82)	0.15 (−0.34, 0.64)	CON

The effects of different BGT Interventions were assessed using a frequency ranking method, and the probability of ranking for each BGT was expressed as a P-score. Results of the network meta-analysis are presented in the left lower half and results from pairwise comparisons in the upper right half, if available. Comparisons between Interventions should be read from left to right and the estimate is in the cell in common between the column-defining Intervention and the row-defining Intervention. In the left lower half, standard mean differences (SMDs) higher than 0 favor the column-defining Intervention, in the upper right half SMDs higher than 0 favor the row defining Intervention. Cells in bold print indicate significant results “.” = not available. AQE-BGT, Aquatic balance and gait training; BGT, balance and gait training; BGT-ECA, balance and gait training with external cues; BWS-TT, body weight supported treadmill training; BWS-TT-ECA, body weight supported treadmill training with external cues; CON, Control group; DT-BGT, dual-task gait training; EC-BGT, eyes closed gait training; RA-GT, robotic-assisted gait training; RA-GT-ECA, robotic-assisted gait training with external cues; TT, treadmill gait training; VR-GT, virtual reality gait training.

#### Proactive balance

Results on proactive balance were reported in 28 studies, in which a total of 749 patients with stroke participated. A mixed comparison of the league table showed that the active balance of the BWS-TT-ECA group improved significantly better than that of the BWS-TT and TT groups; the active balance of the TT-ECA group was significantly better than that of the DT-BGT, TT and CON; and other comparative details are shown in Table 2D. In the overall effect ranking for improving balance, the best intervention was BWS-TT-ECA (P-score: 0.90), while TT was the worst performer (P-score: 0.07; Figure 2). The heterogeneity was moderate (τ^2^ = 0.15, I^2^ = 46.2%, *p* < 0.05), and the Q score of global inconsistency was 7.06 (*p* = 0.5306). Using the nodal split method for local inconsistency testing, we found no hotspots of inconsistency in Appendix 9 (p. 54), indicating good consistency between direct and indirect evidence.

**Table 2D:** League table of proactive balance.

BWS-TT-ECA	.	.	.	.	.	.	.	**1.61 (0.47, 2.75)**	.	.	.
0.53 (−1.17, 2.23)	TT-ECA	.	.	0.40 (−0.79, 1.59)	.	.	.	.	1.17 (−0.04, 2.38)	.	**1.50 (0.82, 2.17)**
0.63 (−1.03, 2.28)	0.10 (−0.73, 0.93)	VR-GT	0.66 (−0.46, 1.78)	.	−0.04 (−0.98, 0.90)	.	.	.	**1.51 (0.38, 2.64)**	.	**1.59 (0.64, 2.55)**
1.04 (−0.71, 2.78)	0.51 (−0.50, 1.52)	0.41 (−0.46, 1.28)	RA-GT-ECA	.	.	0.03 (−1.14, 1.20)	.	.	0.85 (−0.24, 1.94)	.	.
1.08 (−0.51, 2.67)	0.55 (−0.20, 1.31)	0.45 (−0.28, 1.18)	0.04 (−0.85, 0.94)	BGT-ECA	0.31 (−0.26, 0.89)	.	.	.	−0.00 (−0.71, 0.71)	.	.
1.11 (−0.41, 2.63)	0.58 (−0.19, 1.36)	0.48 (−0.17, 1.14)	0.08 (−0.78, 0.93)	0.03 (−0.44, 0.51)	BGT	0.06 (−0.78, 0.91)	.	0.49 (−0.51, 1.50)	0.59 (−0.21, 1.38)	.	.
1.14 (−0.47, 2.76)	0.62 (−0.16, 1.39)	0.51 (−0.19, 1.22)	0.11 (−0.69, 0.90)	0.06 (−0.55, 0.68)	0.03 (−0.51, 0.57)	RA-GT	.	.	0.37 (−0.23, 0.96)	.	**1.11 (0.00, 2.21)**
1.29 (−0.74, 3.31)	0.76 (−0.69, 2.20)	0.66 (−0.75, 2.07)	0.25 (−1.23, 1.73)	0.21 (−1.14, 1.56)	0.17 (−1.16, 1.51)	0.14 (−1.19, 1.48)	EC-BGT	.	0.28 (−0.97, 1.53)	.	.
**1.61 (0.47, 2.75)**	1.08 (−0.19, 2.34)	0.98 (−0.22, 2.18)	0.57 (−0.75, 1.89)	0.53 (−0.59, 1.64)	0.49 (−0.51, 1.50)	0.46 (−0.68, 1.60)	0.32 (−1.35, 1.99)	BWS-TT	.	.	.
1.57 (−0.03, 3.16)	**1.04 (0.31, 1.76)**	**0.94 (0.28, 1.59)**	0.53 (−0.26, 1.32)	0.48 (−0.02, 0.99)	0.45 (−0.02, 0.93)	0.42 (−0.04, 0.89)	0.28 (−0.97, 1.53)	−0.04 (−1.15, 1.07)	CON	.	−0.23 (−1.40, 0.94)
1.78 (−0.20, 3.76)	**1.26 (0.03, 2.48)**	1.16 (−0.11, 2.43)	0.75 (−0.66, 2.16)	0.70 (−0.58, 1.99)	0.67 (−0.60, 1.94)	0.64 (−0.62, 1.90)	0.50 (−1.26, 2.26)	0.18 (−1.44, 1.80)	0.22 (−1.02, 1.46)	DT-BGT	0.28 (−0.78, 1.34)
**2.07 (0.40, 3.74)**	**1.54 (0.92, 2.16)**	**1.44 (0.74, 2.13)**	**1.03 (0.10, 1.96)**	**0.99 (0.26, 1.71)**	**0.95 (0.26, 1.65)**	**0.92 (0.25, 1.60)**	0.78 (−0.63, 2.19)	0.46 (−0.76, 1.68)	0.50 (−0.14, 1.14)	0.28 (−0.78, 1.34)	TT

The effects of different BGT Interventions were assessed using a frequency ranking method, and the probability of ranking for each BGT was expressed as a P-score. Results of the network meta-analysis are presented in the left lower half and results from pairwise comparisons in the upper right half, if available. Comparisons between Interventions should be read from left to right and the estimate is in the cell in common between the column-defining Intervention and the row-defining Intervention. In the left lower half, standard mean differences (SMDs) higher than 0 favor the column-defining Intervention, in the upper right half SMDs higher than 0 favor the row defining Intervention. Cells in bold print indicate significant results “.”= not available. BGT, balance and gait training; BGT-ECA, balance and gait training with external cues; BWS-TT, body weight supported treadmill training; BWS-TT-ECA, body weight supported treadmill training with external cues; CON, Control group; DT-BGT, dual-task gait training; EC-BGT, eyes closed gait training; RA-GT, robotic-assisted gait training; RA-GT-ECA, robotic-assisted gait training with external cues; TT, treadmill gait training; TT-ECA, treadmill gait training with external cues; VR-GT, virtual reality gait training.

### Meta-regression

After regression analysis, we found no significant effect of all covariates on these four balance outcomes, indicating that the heterogeneity of the study did not stem from the age and sex of participants, the duration and frequency of interventions, the year of publication, or the time of entry into the study after stroke [Appendix 8 (p. 46)].

### GRADE assessment

The GRADE approach was used to assess the quality of evidence from studies on the effect of different BGTs on the different balance abilities of patients with stroke through an NMA. Table 3 presents a summary of the certainties of evidence based on four balance types [all details of the GRADE assessment for all pairwise comparisons are presented in Appendix 10 (p. 56)]. The main reasons for downgrading were imprecision, inconsistency, and the risk of bias. However, two hotspots were found through a local inconsistency check of the dynamic steady-state balance, indicating differences in regional direct and indirect comparisons, contributing to the downgrade. The funnel plot was roughly symmetrical, indicating no significant publication bias (Supplementary Figures S14–S17).

**Table 3:** Summary of certainty of evidence (GRADE approach) for network meta-analysis in a study examining the effect of different gait training on different balance abilities in stroke patients.

Outcome	Certainty of evidence	Reason for downgrade
Balance test batteries	Very low	Imprecision, risk of bias
Dynamic steady-state balance	Very low	Imprecision, risk of bias, inconsistency
Static steady-state balance	Very low	Imprecision, risk of bias
Proactive balance	Very low	Imprecision, risk of bias

GRADE, Grading of Recommendations Assessment, Development and Evaluation.

## Discussion

This NMA is the first network meta-analysis to comprehensively assess the effect of various BGTs on the balance of stroke patients. The results found that the effect of different BGT on the different balance abilities of patients with stroke was apparently different, with specific details as follows: VR-GT was the most effective for the balance test batteries of patients with stroke; BWS-TT-ECA was the most effective for their dynamic steady-state balance and proactive balance; and DT-BGT was the most effective for their static steady-state balance. In addition, we did not find that age, gender, duration of illness, year of publication, frequency of interventions, and duration of interventions have a regulatory effect on the BGT effect. Our study provides more clinical options for balance rehabilitation in stroke patients.

The present study showed that VR-GT (SMD: 1.37, 95% CI: 0.62–2.11; P-score: 0.95) was the most effective for the balance test batteries of stroke survivors compared to the CON, which was significantly more effective than many other types of exercise. We found several virtual reality studies on stroke survivors (19, 42, 43), which suggest that virtual reality (VR) training can be more effective in improving balance or gait in stroke patients, which is consistent with our findings. For stroke survivors, optimizing and strengthening the compensatory mechanisms of their brain is crucial for motor impairments (44), and a virtual environment that promotes the illusion of body movements can be created using VR technology, which can enhance the neural activation of motor brain areas, mobilize plastic changes in the neurology of their brain, aid in the recovery of neurological cell synapses, and enable direct training for the central nervous system, which is essential for the reorganization and recovery of neural structures in stroke survivors (45, 46). It is well known that patients with different levels of stroke can undergo different BGT; only those who can walk can undergo traditional treadmill training; those who can walk some distance can undergo weight-supported BGT; and those who cannot walk are more suitable for electromechanical or robot-assisted training (47–49). The type of VR-GT BGT also has all the advantages of VR training, which is more acceptable to patients with stroke, especially for patients with more severe stroke in the early stages of recovery, where the potential for balance recovery is more pronounced. This type of exercise is a valid reason for the additional improvement of the balance test batteries of patients with stroke, which may have contributed to the study’s findings. This NMA also showed a marked effect of BWS-TT-ECA and RA-GT-BGT compared to the CON, which has been included in the interest. Another interesting observation is that TT is the least effective in restoring the balance test batteries of patients with stroke and in studies on proactive balance. One possible explanation is that patients with stroke have reached a new homeostatic state of balance when performing TT BGT, which only maintains their balance, and that to effectively apply BGT in improving patients’ balance, it is necessary to add challenging exercises without reducing their freedom, such as BWS-TT-ECA or BGT-ECA (50, 51).

Dynamic steady-state balance refers to the ability to maintain a stable position while walking, while proactive balance means an equilibrium ability to predict disturbances (52). Research has shown that a habitual gait speed ≤ 1 m/s (dynamic steady-state balance) and the time to complete a Timed Up and Go Test ≥ 13.5 s (proactive balance) increase the risk of falling by 2–3 times (53, 54), and dynamic and static steady-state balance, as well as proactive balance, may be independent of each other (55). Interestingly, the results of the BGT rankings for dynamic steady-state and proactive balance showed a high degree of similarity, with specific details as follows: first, BWS-TT-ECA was the best for both balances (SMD: 1.18, 95% CI: 0.67–1.68; P-score: 0.93); (SMD: 1.57, 95% CI: −0.03–3.16; P-score: 0.90), while the results of proactive balance were not exceptionally stable, with a 95% CI spanning “0”; second, there was some similarity in the order of the remaining rankings of the BGT effect, with both TT-ECA and VR-GT ranking higher; and finally, compared to the CON, both TT-ECA and VR-GT were more effective for dynamic steady-state and proactive balance, both of which were highly significant, suggesting that if both types of balance needed to be rehabilitated simultaneously, similar BGT interventions could be chosen. Although there are many similarities, dynamic steady-state balance is undeniably very different from proactive balance. For dynamic steady-state balance, there are more BGT intervention types available, and in addition to BWS-TT-ECA, TT-ECA, EC-BGT, VR-GT, BWS-TT, RA-GT, BGT-ECA, and BGT also have meaningful effects. However, for proactive balance, TT-ECA and VR-GT were the only two BGT exercises that had a noteworthy effect, with much fewer BGT options. Although they are a specific task in balance performance, for patients with stroke, various balances need to work together to prevent falls.

Compared to CON, DT-BGT, AQE-BGT, EC-BGT, VR-GT, BGT-ECA, BWS-TT-ECA, and RA-GT, we have had significant efficacy for rehabilitating static steady-state balance, indicating that the above BGT was the most promising. DT-BGT had the best therapeutic effect (SMD: 1.64, 95% CI: 0.50–2.78; P-score: 0.91). Previous studies have shown that DT-BGT is effective in improving stride length, stride frequency, cadence, and 10-m walk tests for patients with stroke (15, 56). However, possible advantages in improving the balance function are uncertain, and our study bridges this gap. Regarding static steady-state balance, DT-BGT has an obvious advantage over the other BGT types we have included, but there is no substantial advantage for other balances. Notably, BGT was much more selective (and more pronounced than CON) for improving dynamic and static steady-state balance than improving balance test batteries and proactive balance. For the latter two types of balance, although most BGT has a positive effect on balance, a wide CI crosses “0,” indicating uncertainties in the treatment effect. Previous studies have shown that, after stopping training for 3 months, the ability of healthy older fallers and non-fallers to stand on one leg is significantly reduced (57), which may be more severe for patients with stroke. Although, through a variety of BGT interventions, the static balance of patients with stroke is effectively improved, long-term adherence to training is necessary for obtaining long-term benefits.

### Strengths and limitations

This NMA has several advantages over previous relevant studies. A systematic and comprehensive search strategy for published and unpublished studies based on many databases was applied. Meanwhile, the search was not restricted by publication date or language, and the studies included were not limited to specific types of interventions or comparators; the NMA allows comparisons on the efficacy of different exercise therapies, takes into account the results of direct and indirect comparisons, improves statistical efficiency, and included all relevant studies, which allow us to include a considerable number of RCTs (66 trials; 1,933 patients) and provide a ranking of priorities among different BGT in terms of the efficacy of various balance rehabilitation.

There are still several limitations. First, although we conducted regression analyses with some possible influence as covariates, we did not obtain meaningful results, indicating, on the one hand, that our statistics were relatively stable, while, on the other hand, we did not find a source of heterogeneity. We found high heterogeneity in the results of balance test batteries (τ^2^ = 0.29, I^2^ = 65.5%, *p* < 0.001), which we attributed through discussion to the variability of the outcome, which was a major limitation. Second, the stroke grade or site of onset was reported in only a few studies. Thus, we could not tell whether patients were homogeneous at the time of the initial intervention, and the initial disease grade or the stroke site might have influenced the outcome (58), which might also be an essential source of heterogeneity for this study. Third, we included only each study’s mean and standard deviation rather than the raw data on each patient. Undoubtedly, more precise estimates of different effects could be made based on the data on individual patients, but this was beyond our ability. Fourth, the studies we included did not involve indicators related to the reactive balance of patients with stroke. The specific contribution of reactive balance to falls was undisputed; interventions to improve the balance response due to an unexpected loss of balance were thought to have a more critical impact on the risk of falls (59), and we hoped that, in the future, some investigators would undertake a study in this area. Fifth, when analyzing dynamic steady-state balance inconsistency, we found two hotspots indicating the ambiguity between direct and indirect evidence, illustrating the instability of the results of the outcomes and the need for further validation through high-quality RCTs. Finally, according to the GRADE assessment, our study evidence was of low quality, and the size and ranking of the treatment effect might change as more evidence becomes available. Therefore, more trials need to be included for further investigation.

## Conclusion

This NMA provides evidence that the effect of various BGTs on the balance of patients with stroke is different. Balance is a multidimensional concept, and patients’ needs should be fully considered when selecting BGT. A more effective BGT should be selected to improve patients’ balance ability and reduce adverse falls for them. BGTs that are not statistically meaningful should be cautiously selected because their effectiveness has a higher degree of uncertainty. All findings may help clinicians, patients, and healthcare providers choose more appropriate BGT while recognizing that the quality of the evidence is shallow and that the findings should be interpreted cautiously.

## Data availability statement

The original contributions presented in the study are included in the article/Supplementary material, further inquiries can be directed to the corresponding authors.

## Author contributions

MZ served as principal author and had full access to all the data in the study, taking responsibility for the accuracy of the data analysis, and the integrity of the data. MZ and ZL contributed to the conception and design. MZ, ZL, YL, XJ, and BX contributed to data acquisition and interpretation and draft of the manuscript. TL contributed to revising the article and final approval. All authors contributed to the article and approved the submitted version.
