# Supplementary material for: The effect of balance and gait training on specific balance abilities of survivors with stroke: a systematic review and network meta-analysis
Source: Front Neurol. 2023 Nov 2;14:1234017. doi: 10.3389/fneur.2023.1234017 (PMC10653323; doi:10.3389/fneur.2023.1234017)
Supplement: Supplementary file 2 [file Data_Sheet_2.docx]

**SUPPLEMENTARY INFORMATION**

**The Effect of Balance and Gait Training on Specific Balance Abilities of Survivors with Stroke: A Systematic Review and Network Meta-analysis**

**CONTENTS**

**Table of Contents:**

[**Appendix 1: PRISMA NMA Checklist of Items to Include When Reporting A Systematic Review Involving a Network Meta-analysis** 1](#_Toc142306353)

[**Appendix 2: PROSPERO registration and protocol** 6](#_Toc142306354)

[**Appendix 3: Search strategies** 21](#_Toc142306355)

[**3.1 PubMed:** 21](#_Toc142306356)

[**3.2 Embase:** 23](#_Toc142306357)

[**3.3 Cochrane library:** 23](#_Toc142306358)

[**3.4 Web of Science:** 24](#_Toc142306359)

[**3.5 Scopus** 24](#_Toc142306360)

[**3.6 CNKI:** 26](#_Toc142306361)

[**3.7 Chinese biomedical literature databases** 26](#_Toc142306362)

[**Appendix 4: Demographic characteristics of included studies** 27](#_Toc142306363)

[**Appendix 5: Network graphs of outcomes** 37](#_Toc142306364)

[**Appendix 6: Risk of bias** 38](#_Toc142306365)

[**6.1 Risk of bias graph** 38](#_Toc142306366)

[**6.2 Risk of bias summary** 40](#_Toc142306367)

[**Appendix 7: Forest plot of outcomes** 42](#_Toc142306368)

[**Appendix 8: Network Meta-Regression** 46](#_Toc142306369)

[**Appendix 9: ‘Hot spots’ of inconsistency** 47](#_Toc142306370)

[**9.1 ‘Hot spots’ of inconsistency: balance test batteries** 47](#_Toc142306371)

[**9.2 ‘Hot spots’ of inconsistency: dynamic steady-state balance** 49](#_Toc142306372)

[**9.3 ‘Hot spots’ of inconsistency: static steady-state balance** 52](#_Toc142306373)

[**9.4 ‘Hot spots’ of inconsistency: Proactive balance** 54](#_Toc142306374)

[**Appendix 10: Grading the evidence of the network meta-analysis using GRADE** 56](#_Toc142306375)

[**10.1 Details of GRADE assessment for all pairwise comparisons within the balance test batteries network** 56](#_Toc142306376)

[**10.2 Details of GRADE assessment for all pairwise comparisons within the dynamic steady-state balance network** 59](#_Toc142306377)

[**10.3 Details of GRADE assessment for all pairwise comparisons within the static steady-state balance network** 63](#_Toc142306378)

[**10.4 Details of GRADE assessment for all pairwise comparisons within the proactive balance network** 66](#_Toc142306379)

[**Appendix 11: Network meta-analysis funnel plots** 70](#_Toc142306380)

[**List of included literature** 74](#_Toc142306381)

**Appendix 1: PRISMA NMA Checklist of Items to Include When Reporting A Systematic Review Involving a Network Meta-analysis**

| **Section/Topic** | **Item #** | **Checklist Item** | **Reported on Page #** |
| --- | --- | --- | --- |
| **TITLE** |  |  |  |
| Title | 1 | Identify the report as a systematic review *incorporating a network meta-analysis (or related form of meta-analysis).* | **Page. 1** |
|  |  |  |  |
| **ABSTRACT** |  |  |  |
| Structured summary | 2 | Provide a structured summary including, as applicable:  **Background:** main objectives  **Methods:** data sources; study eligibility criteria, participants, and interventions; study appraisal; and *synthesis methods, such as network meta-analysis.*  **Results:** number of studies and participants identified; summary estimates with corresponding confidence/credible intervals; *treatment rankings may also be discussed. Authors may choose to summarize pairwise comparisons against a chosen treatment included in their analyses for brevity.*  **Discussion/Conclusions:** limitations; conclusions and implications of findings.  **Other:** primary source of funding; systematic review registration number with registry name. | **Page. 1-2** |
|  |  |  |  |
| **INTRODUCTION** |  |  |  |
| Rationale | 3 | Describe the rationale for the review in the context of what is already known*, including mention of why a network meta-analysis has been conducted.* | **Page. 3-4** |
| Objectives | 4 | Provide an explicit statement of questions being addressed, with reference to participants, interventions, comparisons, outcomes, and study design (PICOS). | **Page. 3-4** |
|  |  |  |  |
| **METHODS** |  |  |  |
| Protocol and registration | 5 | Indicate whether a review protocol exists and if and where it can be accessed (e.g., Web address); and, if available, provide registration information, including registration number. | **PROSPERO**  **CRD42022316057**  **Appendix 2** |
| Eligibility criteria | 6 | Specify study characteristics (e.g., PICOS, length of follow-up) and report characteristics (e.g., years considered, language, publication status) used as criteria for eligibility, giving rationale. *Clearly describe eligible treatments included in the treatment network, and note whether any have been clustered or merged into the same node (with justification).* | **Page. 5-6** |
| Information sources | 7 | Describe all information sources (e.g., databases with dates of coverage, contact with study authors to identify additional studies) in the search and date last searched. | **Page. 5** |
| Search | 8 | Present full electronic search strategy for at least one database, including any limits used, such that it could be repeated. | **Appendix 3** |
| Study selection | 9 | State the process for selecting studies (i.e., screening, eligibility, included in systematic review, and, if applicable, included in the meta-analysis). | **Page. 5-6** |
| Data collection process | 10 | Describe method of data extraction from reports (e.g., piloted forms, independently, in duplicate) and any processes for obtaining and confirming data from investigators. | **Page. 6-7** |
| Data items | 11 | List and define all variables for which data were sought (e.g., PICOS, funding sources) and any assumptions and simplifications made. | **Page. 5-6** |
| **Geometry of the network** | **S1** | Describe methods used to explore the geometry of the treatment network under study and potential biases related to it. This should include how the evidence base has been graphically summarized for presentation, and what characteristics were compiled and used to describe the evidence base to readers. | **Page. 8-9** |
| Risk of bias within individual studies | 12 | Describe methods used for assessing risk of bias of individual studies (including specification of whether this was done at the study or outcome level), and how this information is to be used in any data synthesis. | **Page. 7** |
| Summary measures | 13 | State the principal summary measures (e.g., risk ratio, difference in means). *Also describe the use of additional summary measures assessed, such as treatment rankings and surface under the cumulative ranking curve (SUCRA) values, as well as modified approaches used to present summary findings from meta-analyses.* | **Page. 8-9** |
| Planned methods of analysis | 14 | Describe the methods of handling data and combining results of studies for each network meta-analysis. This should include, but not be limited to:   - *Handling of multi-arm trials;* - *Selection of variance structure;* - *Selection of prior distributions in Bayesian analyses; and* - *Assessment of model fit.* | **Page. 8-10** |
| **Assessment of Inconsistency** | **S2** | Describe the statistical methods used to evaluate the agreement of direct and indirect evidence in the treatment network(s) studied. Describe efforts taken to address its presence when found. | **Page. 8-9** |
| Risk of bias across studies | 15 | Specify any assessment of risk of bias that may affect the cumulative evidence (e.g., publication bias, selective reporting within studies). | **Page. 7** |
| Additional analyses | 16 | Describe methods of additional analyses if done, indicating which were pre-specified. This may include, but not be limited to, the following:   - Sensitivity or subgroup analyses; - Meta-regression analyses; - *Alternative formulations of the treatment network; and* - *Use of alternative prior distributions for Bayesian analyses (if applicable).* | **Page. 9** |
|  |  |  |  |
| **RESULTS†** |  |  |  |
| Study selection | 17 | Give numbers of studies screened, assessed for eligibility, and included in the review, with reasons for exclusions at each stage, ideally with a flow diagram. | **Page. 9-10**  **and Figure 1** |
| **Presentation of network structure** | **S3** | Provide a network graph of the included studies to enable visualization of the geometry of the treatment network. | **Figure. S1**  **Appendix 5** |
| **Summary of network geometry** | **S4** | Provide a brief overview of characteristics of the treatment network. This may include commentary on the abundance of trials and randomized patients for the different interventions and pairwise comparisons in the network, gaps of evidence in the treatment network, and potential biases reflected by the network structure. | **Page. 10**  **Appendix 5** |
| Study characteristics | 18 | For each study, present characteristics for which data were extracted (e.g., study size, PICOS, follow-up period) and provide the citations. | **Page. 10**  **Appendix 4** |
| Risk of bias within studies | 19 | Present data on risk of bias of each study and, if available, any outcome level assessment. | **Page. 10-11 and**  **Appendix 6.**  **Figure. S2-9** |
| Results of individual studies | 20 | For all outcomes considered (benefits or harms), present, for each study: 1) simple summary data for each intervention group, and 2) effect estimates and confidence intervals. *Modified approaches may be needed to deal with information from larger networks.* | **Page. 11-13 and**  **Appendix 7.**  **Figure. S10-13** |
| Synthesis of results | 21 | Present results of each meta-analysis done, including confidence/credible intervals. *In larger networks, authors may focus on comparisons versus a particular comparator (e.g. placebo or standard care), with full findings presented in an appendix. League tables and forest plots may be considered to summarize pairwise comparisons.* If additional summary measures were explored (such as treatment rankings), these should also be presented. | **Page. 11-13 and**  **Appendix7.**  **Figure. S10-13**  **Table 2-3** |
| **Exploration for inconsistency** | **S5** | Describe results from investigations of inconsistency. This may include such information as measures of model fit to compare consistency and inconsistency models, *P* values from statistical tests, or summary of inconsistency estimates from different parts of the treatment network. | **Page. 11-13**  **Appendix 9** |
| Risk of bias across studies | 22 | Present results of any assessment of risk of bias across studies for the evidence base being studied. | **Page. 10-11 and**  **Appendix 6.**  **Figure. S2-9** |
| Results of additional analyses | 23 | Give results of additional analyses, if done (e.g., sensitivity or subgroup analyses, meta-regression analyses*, alternative network geometries studied, alternative choice of prior distributions for Bayesian analyses,* and so forth). | **Page. 13-14**  **Appendix 8, 10** |
|  |  |  |  |
| **DISCUSSION** |  |  |  |
| Summary of evidence | 24 | Summarize the main findings, including the strength of evidence for each main outcome; consider their relevance to key groups (e.g., healthcare providers, users, and policy-makers). | **Page. 14-18** |
| Limitations | 25 | Discuss limitations at study and outcome level (e.g., risk of bias), and at review level (e.g., incomplete retrieval of identified research, reporting bias). *Comment on the validity of the assumptions, such as transitivity and consistency. Comment on any concerns regarding network geometry (e.g., avoidance of certain comparisons).* | **Page. 18-19** |
| Conclusions | 26 | Provide a general interpretation of the results in the context of other evidence, and implications for future research. | **Page. 19-20** |
|  |  |  |  |
| **FUNDING** |  |  |  |
| Funding | 27 | Describe sources of funding for the systematic review and other support (e.g., supply of data); role of funders for the systematic review. This should also include information regarding whether funding has been received from manufacturers of treatments in the network and/or whether some of the authors are content experts with professional conflicts of interest that could affect use of treatments in the network. | **The work was supported by the Natural Science Foundation of Shaanxi Province (2014JM2-3027), the Shaanxi Provincial University Youth Innovation Team (201972), and the Ministry of Education Humanities and Social Sciences Research Special Task Project (17YJA890015).** |

PICOS = population, intervention, comparators, outcomes, study design.

* Text in italics indicates wording specific to reporting of network meta-analyses that has been added to guidance from the PRISMA statement.

† Authors may wish to plan for use of appendices to present all relevant information in full detail for items in this section.

**Appendix 2: PROSPERO registration and protocol**


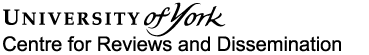


Systematic review

A list of fields that can be edited in an update can be found here

1. * Review title.

Give the title of the review in English

The effect of gait training on balance after stroke: systematic review and network meta-analysis

1. Original language title.

For reviews in languages other than English, give the title in the original language. This will be displayed with the English language title.

1. * Anticipated or actual start date.

Give the date the systematic review started or is expected to start.

30/11/2021

1. [1 change]. * Anticipated completion date.

Give the date by which the review is expected to be completed.

30/04/2023

1. [1 change]. * Stage of review at time of this submission.

**This field uses answers to initial screening questions. It cannot be edited until after registration.**

Tick the boxes to show which review tasks have been started and which have been completed.

Update this field each time any amendments are made to a published record.

The review has not yet started: No

| **Review stage** | **Started** | **Completed** |
| --- | --- | --- |
| Preliminary searches | Yes | No |
| Piloting of the study selection process | Yes | No |
| Formal screening of search results against eligibility criteria | Yes | No |
| Data extraction | Yes | No |
| Risk of bias (quality) assessment | Yes | No |
| Data analysis | Yes | No |

Provide any other relevant information about the stage of the review here.

1. * Named contact.

The named contact is the guarantor for the accuracy of the information in the register record. This may be any member of the review team.

Zhide Liang Email salutation (e.g. "Dr Smith" or "Joanne") for correspondence:

Dr Liang

1. * Named contact email.

Give the electronic email address of the named contact.

869882746@qq.com

1. Named contact address

Give the full institutional/organisational postal address for the named contact.

No.308, Ningxia Road, Shinan District, Qingdao , Shandong Province, China

1. Named contact phone number.

Give the telephone number for the named contact, including international dialling code.

+886-15621471161

1. * Organisational affiliation of the review.

Full title of the organisational affiliations for this review and website address if available. This field may be completed as 'None' if the review is not affiliated to any organisation.

Institute of Physical Education, Qingdao University, Qingdao266000, China

Organisation web address:

https://www.qdu.edu.cn/

1 [1 change]. * Review team members and their organisational affiliations.

Give the personal details and the organisational affiliations of each member of the review team. Affiliation refers to groups or organisations to which review team members belong. **NOTE: email and country now MUST be entered for each person, unless you are amending a published record.**

Mr Meng Zhang. Xi'an Physical Education University, Xi'an710068, China

Mr Zhide Liang. Institute of Physical Education, Qingdao University, Qingdao266000, China

Ms Yali Li. Xi'an Physical Education University, Xi'an710068, China

Mr Xu Jiang. Xi'an Physical Education University, Xi'an710068, China Professor Tao Liu. Xi'an Physical Education University, Xi'an710068, China

1. * Funding sources/sponsors.

Details of the individuals, organizations, groups, companies or other legal entities who have funded or sponsored the review.

None

Grant number(s)

State the funder, grant or award number and the date of award

1. * Conflicts of interest.

List actual or perceived conflicts of interest (financial or academic).

None

1. Collaborators.

Give the name and affiliation of any individuals or organisations who are working on the review but who are not listed as review team members. **NOTE: email and country must be completed for each person, unless you are amending a published record.**

1. * Review question.

State the review question(s) clearly and precisely. It may be appropriate to break very broad questions down into a series of related more specific questions. Questions may be framed or refined using PI(E)COS or similar where relevant.

12?There are many different types of balance and gait training available, and it is still a challenge for cliniciansIs gait training more effec ive than conventional therapy in improving balance in stroke patients?

and patients to choose a better and safe balance and gait training.

1. * Searches.

State the sources that will be searched (e.g. Medline). Give the search dates, and any restrictions (e.g. language or publication date). Do NOT enter the full search strategy (it may be provided as a link or attachment below.)

The systematic review and network meta-analysis were conducted in accordance with the Preferred

Reporting Items for Systematic Reviews and Meta-Analyses (PRISMA) guidelines. We will search the

PubMed, MEDLINE, EMBASE, PsycINFO, Cochrane Library , ClinThere will be no language restrictions. Studies published between inception and the date the searches arecalTrials.gov and Web of Science.

run will be sought. The searches will be re-run just before the final analyses and further studies retrieved for inclusion. We will use a Boolean search strategy with the operators AND, OR, NOT, and the search strategy will include terms describing or relating to intervention, participants, and study design.

1. URL to search strategy.

Upload a file with your search strategy, or an example of a search strategy for a specific database, (including the keywords) in pdf or word format. In doing so you are consenting to the file being made publicly accessible. Or provide a URL or link to the strategy. Do NOT provide links to your search **results**.

https://www.crd.york.ac.uk/PROSPEROFILES/316057_STRATEGY_20220320.pdf

Alternatively, upload your search strategy to CRD in pdf format. Please note that by doing so you are consenting to the file being made publicly accessible.

Yes I give permission for this file to be made publicly available

1. * Condition or domain being studied.

Give a short description of the disease, condition or healthcare domain being studied in your systematic review.

Stroke's disease and exercise

1. * Participants/population.

Specify the participants or populations being studied in the review. The preferred format includes details of both inclusion and exclusion criteria.

Age: 'middle aged and older people' ('45+', no upper age limit, no restriction in setting, gender, ethnicity) with Stroke survivors. unilateral stroke, ranging from at least 30 days from event to a maximum of 90 days.

Functional Ambulatory Category score ?2?able to maintain upright standing posture with moderate assistance.

1. * Intervention(s), exposure(s).

Give full and clear descriptions or definitions of the interventions or the exposures to be reviewed. The preferred format includes details of both inclusion and exclusion criteria.

The intervention to be reviewed is: I. Exercise based gait training and II. Types of exercise intervention in

1multitreatment comparison, including?Conventional physical therapy?

2?Balance and gait training?

3?Robotic-assisted gait training?

4?Body weight support treadmill training?

5?Robotic-assisted gait training?

6?Aquatic exercise?

7?Nordic Walking?

8?Dual task balance and gait training;

9?Balance and gait training with external cue or attention; 10?Treadmill training?

11?Treadmill training with external cue or attention;

12?Body weight support treadmill training with external cue or attention?

13?Robotic-assisted gait training with external cue or attention?

14?Eye closed gait training.

21. * Comparator(s)/control.

Where relevant, give details of the alternatives against which the intervention/exposure will be compared (e.g. another intervention or a non-exposed control group). The preferred format includes details of both inclusion and exclusion criteria.

1. ‘conventional physical therapy’ control was determined based on the report. In ‘conventional physicaltherapy’, participants were expected to continue the routine standard of care provided by their general practitioners.
2. Control groups that were not given any specific intervention such as ‘waiting list’ or usual physical activityor where the authors did not specify the nature of the control were also classified as ‘usual care’. ‘Waitinglist’ controls were given active intervention after a period of observation, with no new intervention being delivered during the trial period.
3. * Types of study to be included.

Give details of the study designs (e.g. RCT) that are eligible for inclusion in the review. The preferred format includes both inclusion and exclusion criteria. If there are no restrictions on the types of study, this should be stated.

We will included open and blinded randomized controlled trials (RCTs) comparing one exercise with another exercise type or control(usual care without exercise intervention). In the case of randomized crossover studies we will use only the first cross-over phase to avoid the problem of carry-over effects which are very likely in exercise. In addition, we will exclude studies on the acute effects of a single exercise on stroke’s patients.

1. Context.

Give summary details of the setting or other relevant characteristics, which help define the inclusion or exclusion criteria.

1. * Main outcome(s).

Give the pre-specified main (most important) outcomes of the review, including details of how the outcome is defined and measured and when these measurement are made, if these are part of the review inclusion criteria.

BalanceSuch as Berg banlance scale, which is a scale that assesses 14 sitting and standing activities, each on atest battery. 5-point scale, with a maximum score of 56.

Measures of effect

Please specify the effect measure(s) for you main outcome(s) e.g. relative risks, odds ratios, risk difference, and/or 'number needed to treat.

Timing: The specified end point of the trial will be used asEffect measure: Standardised mean difference at the end of the study the end-point in the assessment.

1. * Additional outcome(s).

List the pre-specified additional outcomes of the review, with a similar level of detail to that required for main outcomes. Where there are no additional outcomes please state ‘None’ or ‘Not applicable’ as appropriate to the review

Other balance test battery?

Such as Tinetti test, Activities-specific balance confidence scale, and Trunk impairment scale(total).

Dynamic steady-state balance

Such as 10m walk test, Motricity Index, Walking speed, Mini-BESTest, 5m walk test, Trunk impairment scale (dynamic), 8m walk test, and Functional ambulation category.

Static steady-state balance

Such as Sensory organization test, Static gait balance, Single limb stance, Postural assessment scale for stroke, Postural sway velocity moment, Postural sway path length, Biodex Medical Systems overall, Romberg, eyes open(closed), Trunk Impairment Scale (static), and Single support center of pressure symmetry

Proactive balance

Such as Time up go, Community walk test

Measures of effect

Please specify the effect measure(s) for you additional outcome(s) e.g. relative risks, odds ratios, risk difference, and/or 'number needed to treat.

Timing: The specified end point of the trial will be used as the end-point in the assessment.

Effect measure: Standardized mean difference in patient balance at the end of the study. Balance test battery

(such as Berg balance scale, Tinetti test, Activities-specific balance confidence scale, and Trunk impairment scale[total]),dynamic steady-state balance (such as 10m walk test, Motricity Index, Walking speed, MiniBESTest, 5m walk test, Trunk impairment scale [dynamic], 8m walk test, and Functional ambulation category),Static steady-state balance (such as Sensory organization test, Static gait balance, Single limb stance, Postural assessment scale for stroke, Postural sway velocity moment, Postural sway path length, Biodex Medical Systems overall, Romberg, eyes open(closed), Trunk Impairment Scale [static], and Single support center of pressure symmetry, Proactive balance(such as Time up go, Community walk test).

1. * Data extraction (selection and coding).

Describe how studies will be selected for inclusion. State what data will be extracted or obtained. State how this will be done and recorded.

All search results will be exported into EndNote and duplicates will be removed. Titles and abstracts from the initial literature search will be independently assessed by two reviewers (Z.D.L. and M.Z.). Full texts for articles deemed eligible for inclusion from the title and abstract search by either reviewer, in addition to those where no decision could be reached by the reviewers from this initial screen, will be screened independently by two researchers (Z.D.L. and M.Z.). Any discrepancies will be resolved by discussion with all researchers in the review team. Two reviewers (Z.D.L. and M.Z.) will independently extract data from the final inclusion list of articles into a standardised data extraction spreadsheet in Excel. At this stage, two authors extracted information on (1) relevant data regarding participant characteristics (e.g., the sample size, age, and sex); (2) training pattern; (3) training variable (e.g., duration, frequency, sets, repetitions, and intensity); (4) Years of diagnosis; (5) Functional ambulation category and berg balance scale and (6) the main result of the study. In case of incomplete raw data availability, we contacted the corresponding author of the manuscript. We excluded the studies of which the authors could not be reached. All studies were assessed independently in this systematic review by two researchers (Z.D.L. and M.Z.) based on the extracted information. If there were any disagreements about the inclusion of a study, a third reviewer (Y.Y.) was consulted.

1. * Risk of bias (quality) assessment.

State which characteristics of the studies will be assessed and/or any formal risk of bias/quality assessment tools that will be used.

Study quality in terms of sequence generation, allocation concealment, blinding, the completeness of outcome data, selective reporting and other biases will be assessed with the Cochrane Collaboration risk of bias tool.Two reviewers (Z.D.L. and M.Z.) scored the studies according to the proposed scale. In case of disagreements, a consensus was adopted or, if necessary, a third reviewer evaluated the article (Y.Y.).

1. * Strategy for data synthesis.

Describe the methods you plan to use to synthesise data. This **must not be generic text** but should be **specific to your review** and describe how the proposed approach will be applied to your data. If metaanalysis is planned, describe the models to be used, methods to explore statistical heterogeneity, and software package to be used.

We will use R software gemtc and rjags packages to perform Bayesian network meta-analysis. We will use arm-level data and import into the r software in CSV format. We estimated the summary the standardized mean difference of continuous outcomes (SMD, Cohen’ s d) and odds ratio (OR) of dichotomous outcomes. The binomial likelihood was used for dichotomous outcomes and the normal likelihood for continuous outcomes. The study effect sizes were then synthesized using a random-effects network meta-analysis model. In addition, we will present the summary SMD, 95% credible intervals (CrIs) or ORs, 95% CrIs for all pairwise comparisons in the league table, and we show the results of comparing the outcomes of each exercise intervention group and the control group in the form of a forest plot. To rank the various treatments for each outcome, we will use the surface under the cumulative ranking curve (SUCRA). The effect size measure for continuous outcomes chooses the SMD because the studies use different rating scales, such as balance test battery?dynamic steady-state balance?static steady state balance?proactive balance.If the original study reported a standard error in the experimental and control groups, the standard deviation was calculated by the formula: standard deviation (SD) = standard error (SE) × ?n. If both are missing, we will estimate SD based on the confidence interval, t-value, quartile, range, or p-values as described in Section

7.7.3 of the Cochrane Handbook for Systematic Reviews. When only figures were data were extracted using GetData (http://getdata-graph-digitizer.com) to measure the length (in pixels) of the axes to calibrate and then the length in pixels from the relevant axis to the data points of interest. If the data needed for the study cannot be extracted from the above methods, we will ask the authors about the data at least 4 times within 6 weeks.

1. * Analysis of subgroups or subsets.

State any planned investigation of ‘subgroups’. Be clear and specific about which type of study or participant will be included in each group or covariate investigated. State the planned analytic approach. The following potential effect moderators of the primary outcome will be explored by subgroup or metaregression analysis: sample size, mean age, percentage male, years of diagnosis, disease grade, exercise duration, exercise intensity, supervision, and exercise class form. In addition, a sensitivity analysis will be conducted which will exclude studies deemed to be high risk of bias.

1. * Type and method of review.

Select the type of review, review method and health area from the lists below.

Type of review

Cost effectiveness

No

Diagnostic

No

Epidemiologic

No

Individual patient data (IPD) meta-analysis

No

Intervention

No

Living systematic review

No

Meta-analysis

No

Methodology

No

Narrative synthesis

No

Network meta-analysis

Yes

Pre-clinical

No

Prevention

No

Prognostic

No

Prospective meta-analysis (PMA)

No

Review of reviews

No

Service delivery

No

Synthesis of qualitative studies

No

Systematic review

Yes

Other

No

Health area of the review

Alcohol/substance misuse/abuse

No

Blood and immune system

No

Cancer

No

Cardiovascular

No

Care of the elderly

No

Child health

No

Complementary therapies

No

COVID-19

No

Crime and justice

No

Dental

No

Digestive system

No

Ear, nose and throat

No

Education

No

Endocrine and metabolic disorders

No

Eye disorders

No

General interest

No

Genetics

No

Health inequalities/health equity

No

Infections and infestations

No

International development

No

Mental health and behavioural conditions

No

Musculoskeletal

No

Neurological

No

Nursing

No

Obstetrics and gynaecology

No

Oral health

No

Palliative care

No

Perioperative care

No

Physiotherapy

No

Pregnancy and childbirth

No

Public health (including social determinants of health)

No

Rehabilitation

Yes

Respiratory disorders

No

Service delivery

No

Skin disorders

No

Social care

No

Surgery

No

Tropical Medicine

No

Urological

No

Wounds, injuries and accidents

No

Violence and abuse

No

1. Language.

Select each language individually to add it to the list below, use the bin icon to remove any added in error.

English

There is an English language summary.

1. * Country.

Select the country in which the review is being carried out. For multi-national collaborations select all the countries involved.

China

1. Other registration details.

Name any other organisation where the systematic review title or protocol is registered (e.g. Campbell, or The Joanna Briggs Institute) together with any unique identification number assigned by them. If extracted data will be stored and made available through a repository such as the Systematic Review Data Repository (SRDR), details and a link should be included here. If none, leave blank.

1. Reference and/or URL for published protocol.

If the protocol for this review is published provide details (authors, title and journal details, preferably in Vancouver format)

Add web link to the published protocol.

Or, upload your published protocol here in pdf format. Note that the upload will be publicly accessible.

No I do not make this file publicly available until the review is complete

Please note that the information required in the PROSPERO registration form must be completed in full even if access to a protocol is given.

1. Dissemination plans.

Do you intend to publish the review on completion?

Yes

Give brief details of plans for communicating review findings.?

1. Keywords.

Give words or phrases that best describe the review. Separate keywords with a semicolon or new line. Keywords help PROSPERO users find your review (keywords do not appear in the public record but are included in searches). Be as specific and precise as possible. Avoid acronyms and abbreviations unless these are in wide use.

stroke; gait training; intervention; meta-analysis

1. Details of any existing review of the same topic by the same authors.

If you are registering an update of an existing review give details of the earlier versions and include a full bibliographic reference, if available.

38 [1 change]. * Current review status.

Update review status when the review is completed and when it is published.New registrations must be ongoing so this field is not editable for initial submission.

Please provide anticipated publication date

Review_Ongoing

1. Any additional information.

Provide any other information relevant to the registration of this review.

This study is only part of a randomized controlled trial. This study was used to find the best type of gait training to improve daily living skills for stroke survivors.

1. Details of final report/publication(s) or preprints if available.

Leave empty until publication details are available OR you have a link to a preprint (NOTE: this field is not editable for initial submission). List authors, title and journal details preferably in Vancouver format.

Give the link to the published review or preprint.

**Appendix 3: Search strategies**

**3.1 PubMed:**

#1 "Stroke"[Mesh]

#2 (((((((((((((((((((((((((((Strokes[Title/Abstract]) OR (Cerebrovascular Accident[Title/Abstract])) OR (Cerebrovascular Accidents[Title/Abstract])) OR (CVA (Cerebrovascular Accident[Title/Abstract]))) OR (CVAs (Cerebrovascular Accident[Title/Abstract]))) OR (Cerebrovascular Apoplexy[Title/Abstract])) OR (Apoplexy, Cerebrovascular[Title/Abstract])) OR (Vascular Accident, Brain[Title/Abstract])) OR (Brain Vascular Accident[Title/Abstract])) OR (Brain Vascular Accidents[Title/Abstract])) OR (Vascular Accidents, Brain[Title/Abstract])) OR (Cerebrovascular Stroke[Title/Abstract])) OR (Cerebrovascular Strokes[Title/Abstract])) OR (Stroke, Cerebrovascular[Title/Abstract])) OR (Strokes, Cerebrovascular[Title/Abstract])) OR (Apoplexy[Title/Abstract])) OR (Cerebral Stroke[Title/Abstract])) OR (Cerebral Strokes[Title/Abstract])) OR (Stroke, Cerebral[Title/Abstract])) OR (Strokes, Cerebral[Title/Abstract])) OR (Stroke, Acute[Title/Abstract])) OR (Acute Stroke[Title/Abstract])) OR (Acute Strokes[Title/Abstract])) OR (Strokes, Acute[Title/Abstract])) OR (Cerebrovascular Accident, Acute[Title/Abstract])) OR (Acute Cerebrovascular Accident[Title/Abstract])) OR (Acute Cerebrovascular Accidents[Title/Abstract])) OR (Cerebrovascular Accidents, Acute[Title/Abstract])

#3 ("Stroke"[Mesh]) OR ((((((((((((((((((((((((((((Strokes[Title/Abstract]) OR (Cerebrovascular Accident[Title/Abstract])) OR (Cerebrovascular Accidents[Title/Abstract])) OR (CVA (Cerebrovascular Accident[Title/Abstract]))) OR (CVAs (Cerebrovascular Accident[Title/Abstract]))) OR (Cerebrovascular Apoplexy[Title/Abstract])) OR (Apoplexy, Cerebrovascular[Title/Abstract])) OR (Vascular Accident, Brain[Title/Abstract])) OR (Brain Vascular Accident[Title/Abstract])) OR (Brain Vascular Accidents[Title/Abstract])) OR (Vascular Accidents, Brain[Title/Abstract])) OR (Cerebrovascular Stroke[Title/Abstract])) OR (Cerebrovascular Strokes[Title/Abstract])) OR (Stroke, Cerebrovascular[Title/Abstract])) OR (Strokes, Cerebrovascular[Title/Abstract])) OR (Apoplexy[Title/Abstract])) OR (Cerebral Stroke[Title/Abstract])) OR (Cerebral Strokes[Title/Abstract])) OR (Stroke, Cerebral[Title/Abstract])) OR (Strokes, Cerebral[Title/Abstract])) OR (Stroke, Acute[Title/Abstract])) OR (Acute Stroke[Title/Abstract])) OR (Acute Strokes[Title/Abstract])) OR (Strokes, Acute[Title/Abstract])) OR (Cerebrovascular Accident, Acute[Title/Abstract])) OR (Acute Cerebrovascular Accident[Title/Abstract])) OR (Acute Cerebrovascular Accidents[Title/Abstract])) OR (Cerebrovascular Accidents, Acute[Title/Abstract]))

#4 "Exercise"[Mesh]

#5 (((((((((((((((((((((((((Exercises[Title/Abstract]) OR (Physical Activity[Title/Abstract])) OR (Activities, Physical[Title/Abstract])) OR (Activity, Physical[Title/Abstract])) OR (Physical Activities[Title/Abstract])) OR (Exercise, Physical[Title/Abstract])) OR (Exercises, Physical[Title/Abstract])) OR (Physical Exercise[Title/Abstract])) OR (Physical Exercises[Title/Abstract])) OR (Acute Exercise[Title/Abstract])) OR (Acute Exercises[Title/Abstract])) OR (Exercise, Acute[Title/Abstract])) OR (Exercises, Acute[Title/Abstract])) OR (Exercise, Isometric[Title/Abstract])) OR (Exercises, Isometric[Title/Abstract])) OR (Isometric Exercises[Title/Abstract])) OR (Isometric Exercise[Title/Abstract])) OR (Exercise, Aerobic[Title/Abstract])) OR (Aerobic Exercise[Title/Abstract])) OR (Aerobic Exercises[Title/Abstract])) OR (Exercises, Aerobic[Title/Abstract])) OR (Exercise Training[Title/Abstract])) OR (Exercise Trainings[Title/Abstract])) OR (Training, Exercise[Title/Abstract])) OR (Trainings, Exercise[Title/Abstract])) OR (training[Title/Abstract])

#6 ((((((((((((((((((((((((((Exercises[Title/Abstract]) OR (Physical Activity[Title/Abstract])) OR (Activities, Physical[Title/Abstract])) OR (Activity, Physical[Title/Abstract])) OR (Physical Activities[Title/Abstract])) OR (Exercise, Physical[Title/Abstract])) OR (Exercises, Physical[Title/Abstract])) OR (Physical Exercise[Title/Abstract])) OR (Physical Exercises[Title/Abstract])) OR (Acute Exercise[Title/Abstract])) OR (Acute Exercises[Title/Abstract])) OR (Exercise, Acute[Title/Abstract])) OR (Exercises, Acute[Title/Abstract])) OR (Exercise, Isometric[Title/Abstract])) OR (Exercises, Isometric[Title/Abstract])) OR (Isometric Exercises[Title/Abstract])) OR (Isometric Exercise[Title/Abstract])) OR (Exercise, Aerobic[Title/Abstract])) OR (Aerobic Exercise[Title/Abstract])) OR (Aerobic Exercises[Title/Abstract])) OR (Exercises, Aerobic[Title/Abstract])) OR (Exercise Training[Title/Abstract])) OR (Exercise Trainings[Title/Abstract])) OR (Training, Exercise[Title/Abstract])) OR (Trainings, Exercise[Title/Abstract])) OR (training[Title/Abstract])) OR ("Exercise"[Mesh])

#7 balance[Title/Abstract]

#8 (randomized controlled trial[Publication Type] OR randomized[Title/Abstract] OR placebo[Title/Abstract])

#9 (((("Stroke"[Mesh]) OR ((((((((((((((((((((((((((((Strokes[Title/Abstract]) OR (Cerebrovascular Accident[Title/Abstract])) OR (Cerebrovascular Accidents[Title/Abstract])) OR (CVA (Cerebrovascular Accident[Title/Abstract]))) OR (CVAs (Cerebrovascular Accident[Title/Abstract]))) OR (Cerebrovascular Apoplexy[Title/Abstract])) OR (Apoplexy, Cerebrovascular[Title/Abstract])) OR (Vascular Accident, Brain[Title/Abstract])) OR (Brain Vascular Accident[Title/Abstract])) OR (Brain Vascular Accidents[Title/Abstract])) OR (Vascular Accidents, Brain[Title/Abstract])) OR (Cerebrovascular Stroke[Title/Abstract])) OR (Cerebrovascular Strokes[Title/Abstract])) OR (Stroke, Cerebrovascular[Title/Abstract])) OR (Strokes, Cerebrovascular[Title/Abstract])) OR (Apoplexy[Title/Abstract])) OR (Cerebral Stroke[Title/Abstract])) OR (Cerebral Strokes[Title/Abstract])) OR (Stroke, Cerebral[Title/Abstract])) OR (Strokes, Cerebral[Title/Abstract])) OR (Stroke, Acute[Title/Abstract])) OR (Acute Stroke[Title/Abstract])) OR (Acute Strokes[Title/Abstract])) OR (Strokes, Acute[Title/Abstract])) OR (Cerebrovascular Accident, Acute[Title/Abstract])) OR (Acute Cerebrovascular Accident[Title/Abstract])) OR (Acute Cerebrovascular Accidents[Title/Abstract])) OR (Cerebrovascular Accidents, Acute[Title/Abstract]))) AND (((((((((((((((((((((((((((Exercises[Title/Abstract]) OR (Physical Activity[Title/Abstract])) OR (Activities, Physical[Title/Abstract])) OR (Activity, Physical[Title/Abstract])) OR (Physical Activities[Title/Abstract])) OR (Exercise, Physical[Title/Abstract])) OR (Exercises, Physical[Title/Abstract])) OR (Physical Exercise[Title/Abstract])) OR (Physical Exercises[Title/Abstract])) OR (Acute Exercise[Title/Abstract])) OR (Acute Exercises[Title/Abstract])) OR (Exercise, Acute[Title/Abstract])) OR (Exercises, Acute[Title/Abstract])) OR (Exercise, Isometric[Title/Abstract])) OR (Exercises, Isometric[Title/Abstract])) OR (Isometric Exercises[Title/Abstract])) OR (Isometric Exercise[Title/Abstract])) OR (Exercise, Aerobic[Title/Abstract])) OR (Aerobic Exercise[Title/Abstract])) OR (Aerobic Exercises[Title/Abstract])) OR (Exercises, Aerobic[Title/Abstract])) OR (Exercise Training[Title/Abstract])) OR (Exercise Trainings[Title/Abstract])) OR (Training, Exercise[Title/Abstract])) OR (Trainings, Exercise[Title/Abstract])) OR (training[Title/Abstract])) OR ("Exercise"[Mesh]))) AND (balance[Title/Abstract])) AND ((randomized controlled trial[Publication Type] OR randomized[Title/Abstract] OR placebo[Title/Abstract]))

**3.2 Embase:**

#1 'stroke'/exp

#2 'strokes':ab,ti OR 'cerebrovascular accident':ab,ti OR 'cerebrovascular accidents':ab,ti OR 'cva (cerebrovascular accident)':ab,ti OR 'cvas (cerebrovascular accident)':ab,ti OR 'cerebrovascular apoplexy':ab,ti OR 'apoplexy, cerebrovascular':ab,ti OR 'vascular accident, brain':ab,ti OR 'brain vascular accident':ab,ti OR 'brain vascular accidents':ab,ti OR 'vascular accidents, brain':ab,ti OR 'cerebrovascular stroke':ab,ti OR 'cerebrovascular strokes':ab,ti OR 'stroke, cerebrovascular':ab,ti OR 'strokes, cerebrovascular':ab,ti OR 'apoplexy':ab,ti OR 'cerebral stroke':ab,ti OR 'cerebral strokes':ab,ti OR 'stroke, cerebral':ab,ti OR 'strokes, cerebral':ab,ti OR 'stroke, acute':ab,ti OR 'acute stroke':ab,ti OR 'acute strokes':ab,ti OR 'strokes, acute':ab,ti OR 'cerebrovascular accident, acute':ab,ti OR 'acute cerebrovascular accident':ab,ti OR 'acute cerebrovascular accidents':ab,ti OR 'cerebrovascular accidents, acute':ab,ti

#3 #1 OR #2

#4 'exercise'/exp

#5 'exercises':ab,ti OR 'physical activity':ab,ti OR 'activities, physical':ab,ti OR 'activity, physical':ab,ti OR 'physical activities':ab,ti OR 'exercise, physical':ab,ti OR 'exercises, physical':ab,ti OR 'physical exercise':ab,ti OR 'physical exercises':ab,ti OR 'acute exercise':ab,ti OR 'acute exercises':ab,ti OR 'exercise, acute':ab,ti OR 'exercises, acute':ab,ti OR 'exercise, isometric':ab,ti OR 'exercises, isometric':ab,ti OR 'isometric exercises':ab,ti OR 'isometric exercise':ab,ti OR 'exercise, aerobic':ab,ti OR 'aerobic exercise':ab,ti OR 'aerobic exercises':ab,ti OR 'exercises, aerobic':ab,ti OR 'exercise training':ab,ti OR 'exercise trainings':ab,ti OR 'training, exercise':ab,ti OR 'trainings, exercise':ab,ti OR 'training':ab,ti

#6 #4 OR #5

#7 'randomized controlled trial':ab,ti OR 'randomized':ab,ti OR 'placebo':ab,ti

#8 'balance':ab,ti

#9 #3 AND #6 AND #7 AND #8

**3.3 Cochrane library:**

#1 MeSH descriptor: [Stroke] explode all trees

#2 (Strokes):ti,ab,kw OR (Cerebrovascular Accident):ti,ab,kw OR (Cerebrovascular Accidents):ti,ab,kw OR (CVA (Cerebrovascular Accident)):ti,ab,kw OR (CVAs (Cerebrovascular Accident)):ti,ab,kw OR (Cerebrovascular Apoplexy):ti,ab,kw OR (Apoplexy, Cerebrovascular):ti,ab,kw OR (Vascular Accident, Brain):ti,ab,kw OR (Brain Vascular Accident):ti,ab,kw OR (Brain Vascular Accidents):ti,ab,kw OR (Vascular Accidents, Brain):ti,ab,kw OR (Cerebrovascular Stroke):ti,ab,kw OR (Cerebrovascular Strokes):ti,ab,kw OR (Stroke, Cerebrovascular):ti,ab,kw OR (Strokes, Cerebrovascular):ti,ab,kw OR (Apoplexy):ti,ab,kw OR (Cerebral Stroke):ti,ab,kw OR (Cerebral Strokes):ti,ab,kw OR (Stroke, Cerebral):ti,ab,kw OR (Strokes, Cerebral):ti,ab,kw OR (Stroke, Acute):ti,ab,kw OR (Acute Stroke):ti,ab,kw OR (Acute Strokes):ti,ab,kw OR (Strokes, Acute):ti,ab,kw OR (Cerebrovascular Accident, Acute):ti,ab,kw OR (Acute Cerebrovascular Accident):ti,ab,kw OR (Acute Cerebrovascular Accidents):ti,ab,kw OR (Cerebrovascular Accidents, Acute):ti,ab,kw

#3 #1 or #2

#4 MeSH descriptor: [Exercise] explode all trees

#5 (Exercises):ti,ab,kw OR (Physical Activity):ti,ab,kw OR (Activities, Physical):ti,ab,kw OR (Activity, Physical):ti,ab,kw OR (Physical Activities):ti,ab,kw OR (Exercise, Physical):ti,ab,kw OR (Exercises, Physical):ti,ab,kw OR (Physical Exercise):ti,ab,kw OR (Physical Exercises):ti,ab,kw OR (Acute Exercise):ti,ab,kw OR (Acute Exercises):ti,ab,kw OR (Exercise, Acute):ti,ab,kw OR (Exercises, Acute):ti,ab,kw OR (Exercise, Isometric):ti,ab,kw OR (Exercises, Isometric):ti,ab,kw OR (Isometric Exercises):ti,ab,kw OR (Isometric Exercise):ti,ab,kw OR (Exercise, Aerobic):ti,ab,kw OR (Aerobic Exercise):ti,ab,kw OR (Aerobic Exercises):ti,ab,kw OR (Exercises, Aerobic):ti,ab,kw OR (Exercise Training):ti,ab,kw OR (Exercise Trainings):ti,ab,kw OR (Training, Exercise):ti,ab,kw OR (Trainings, Exercise):ti,ab,kw OR (Training):ti,ab,kw

#6 #4 or #5

#7 (balance):ti,ab,kw

#8 #3 and #6 and #7

**3.4 Web of Science:**

#1 TS=(Stroke or Strokes or Cerebrovascular Accident or Cerebrovascular Accidents or CVA (Cerebrovascular Accident) or CVAs (Cerebrovascular Accident) or Cerebrovascular Apoplexy or Apoplexy, Cerebrovascular or Vascular Accident, Brain or Brain Vascular Accident or Brain Vascular Accidents or Vascular Accidents, Brain or Cerebrovascular Stroke or Cerebrovascular Strokes or Stroke, Cerebrovascular or Strokes, Cerebrovascular or Apoplexy or Cerebral Stroke or Cerebral Strokes or Stroke, Cerebral or Strokes, Cerebral or Stroke, Acute or Acute Stroke or Acute Strokes or Strokes, Acute or Cerebrovascular Accident, Acute or Acute Cerebrovascular Accident or Acute Cerebrovascular Accidents or Cerebrovascular Accidents, Acute)

#2 TS=(Exercise or Exercises or Physical Activity or Activities, Physical or Activity, Physical or Physical Activities or Exercise, Physical or Exercises, Physical or Physical Exercise or Physical Exercises or Acute Exercise or Acute Exercises or Exercise, Acute or Exercises, Acute or Exercise, Isometric or Exercises, Isometric or Isometric Exercises or Isometric Exercise or Exercise, Aerobic or Aerobic Exercise or Aerobic Exercises or Exercises, Aerobic or Exercise Training or Exercise Trainings or Training, Exercise or Trainings, Exercise or Training)

#3 TS=(randomized controlled trial or randomized or placebo)

#4 TS=(balance)

#5 #1 AND #2 AND #3 AND #4

**3.5 Scopus**

#1 ( TITLE-ABS-KEY ( stroke ) OR TITLE-ABS-KEY ( strokes ) OR TITLE-ABS-KEY ( cerebrovascular AND accidents ) OR TITLE-ABS-KEY ( cerebrovascular AND accident ) OR TITLE-ABS-KEY ( cerebrovascular AND apoplexy ) OR TITLE-ABS-KEY ( apoplexy, AND cerebrovascular ) OR TITLE-ABS-KEY ( vascular AND accident, AND brain ) OR TITLE-ABS-KEY ( brain AND vascular AND accident ) OR TITLE-ABS-KEY ( brain AND vascular AND accidents ) OR TITLE-ABS-KEY ( vascular AND accidents, AND brain ) OR TITLE-ABS-KEY ( cerebrovascular stroke ) OR TITLE-ABS-KEY ( cerebrovascular AND strokes ) OR TITLE-ABS-KEY ( stroke, AND cerebrovascular ) OR TITLE-ABS-KEY ( apoplexy ) OR TITLE-ABS-KEY ( cerebral stroke ) OR TITLE-ABS-KEY ( cerebral AND strokes ) OR TITLE-ABS-KEY ( stroke, AND cerebral ) OR TITLE-ABS-KEY ( acute stroke ) OR TITLE-ABS-KEY ( acute AND cerebrovascular AND accident ) )

#2 ( TITLE-ABS-KEY ( exercise ) OR TITLE-ABS-KEY ( exercises ) OR TITLE-ABS-KEY ( physical AND activity ) OR TITLE-ABS-KEY ( activities, AND physical ) OR TITLE-ABS-KEY ( activity, AND physical ) OR TITLE-ABS-KEY ( physical AND activities ) OR TITLE-ABS-KEY ( exercise, AND physical ) OR TITLE-ABS-KEY ( exercises, AND physical ) OR TITLE-ABS-KEY ( physical exercise ) OR TITLE-ABS-KEY ( physical AND exercises ) OR TITLE-ABS-KEY ( exercise, AND acute ) OR TITLE-ABS-KEY ( training, exercise ) OR TITLE-ABS-KEY ( exercise, AND isometric ) OR TITLE-ABS-KEY ( exercise training ) OR TITLE-ABS-KEY ( isometric AND exercises ) OR TITLE-ABS-KEY ( isometric exercise ) OR TITLE-ABS-KEY ( stroke, AND cerebral ) OR TITLE-ABS-KEY ( exercise, AND aerobic ) OR TITLE-ABS-KEY ( aerobic exercise ) )

#3 ( TITLE-ABS-KEY ( randomized AND controlled AND trial ) OR TITLE-ABS-KEY ( randomized ) OR TITLE-ABS-KEY ( placebo ) )

#4 TITLE-ABS-KEY ( balance )

#5 ( ( TITLE-ABS-KEY ( stroke ) OR TITLE-ABS-KEY ( strokes ) OR TITLE-ABS-KEY ( cerebrovascular AND accidents ) OR TITLE-ABS-KEY ( cerebrovascular AND accident ) OR TITLE-ABS-KEY ( cerebrovascular AND apoplexy ) OR TITLE-ABS-KEY ( apoplexy, AND cerebrovascular ) OR TITLE-ABS-KEY ( vascular AND accident, AND brain ) OR TITLE-ABS-KEY ( brain AND vascular AND accident ) OR TITLE-ABS-KEY ( brain AND vascular AND accidents ) OR TITLE-ABS-KEY ( vascular AND accidents, AND brain ) OR TITLE-ABS-KEY ( cerebrovascular stroke ) OR TITLE-ABS-KEY ( cerebrovascular AND strokes ) OR TITLE-ABS-KEY ( stroke, AND cerebrovascular ) OR TITLE-ABS-KEY ( apoplexy ) OR TITLE-ABS-KEY ( cerebral stroke ) OR TITLE-ABS-KEY ( cerebral AND strokes ) OR TITLE-ABS-KEY ( stroke, AND cerebral ) OR TITLE-ABS-KEY ( acute stroke ) OR TITLE-ABS-KEY ( acute AND cerebrovascular AND accident ) ) ) AND ( ( TITLE-ABS-KEY ( exercise ) OR TITLE-ABS-KEY ( exercises ) OR TITLE-ABS-KEY ( physical AND activity ) OR TITLE-ABS-KEY ( activities, AND physical ) OR TITLE-ABS-KEY ( activity, AND physical ) OR TITLE-ABS-KEY ( physical AND activities ) OR TITLE-ABS-KEY ( exercise, AND physical ) OR TITLE-ABS-KEY ( exercises, AND physical ) OR TITLE-ABS-KEY ( physical exercise ) OR TITLE-ABS-KEY ( physical AND exercises ) OR TITLE-ABS-KEY ( exercise, AND acute ) OR TITLE-ABS-KEY ( training, exercise ) OR TITLE-ABS-KEY ( exercise, AND isometric ) OR TITLE-ABS-KEY ( exercise training ) OR TITLE-ABS-KEY ( isometric AND exercises ) OR TITLE-ABS-KEY ( isometric exercise ) OR TITLE-ABS-KEY ( stroke, AND cerebral ) OR TITLE-ABS-KEY ( exercise, AND aerobic ) OR TITLE-ABS-KEY ( aerobic exercise ) ) ) AND ( ( TITLE-ABS-KEY ( randomized AND controlled AND trial ) OR TITLE-ABS-KEY ( randomized ) OR TITLE-ABS-KEY ( placebo ) ) ) AND ( TITLE-ABS-KEY ( balance ) )

**3.6 CNKI:**

#1 SU=Stroke OR SU= Cerebrovascular event OR SU= Cerebrovascular accident OR SU= Cerebrovascular incident OR SU=Brain attack OR SU=Stroke

#2 SU= randomized controlled trial OR SU= randomized

#3 SU=Exercise OR SU=Training

#4 TKA=balance

#5 #1 AND #2 AND #3 AND #4

**3.7 Chinese biomedical literature databases**

#1 "Stroke" [title: Intelligence] OR "Stroke" [title: Intelligence] OR "Cerebrovascular event" [title: Intelligence] OR "Cerebrovascular accident" [title: Intelligence] OR "Cerebrovascular incident " [title: Intelligence] OR " Brain attack " [title: Intelligence]

#2 "Randomised controlled experiment" [title: Intelligent] OR "Randomised" [title: Intelligent]

#3 "Exercise" [title: Intelligence] OR "Training" [title: Intelligence]

#4 "Balance" [Abstract: Intelligence]

#5 ("Balance" [abstract: Intelligent]) AND ("Exercise" [title: Intelligent] OR "Training" [title: Intelligent]) AND ("Randomised Controlled Experiment" [title: Intelligent] OR "Randomised" [title: Intelligent]) AND ("Stroke" [title: Intelligent] OR "Stroke" [title: Intelligent] OR "Cerebrovascular Event" [title: Intelligent] OR "Cerebrovascular Accident" [ Title: Intelligent] OR "Cerebrovascular incident "[Title: Intelligent] OR " Brain attack "[Title: Intelligent])

**Appendix 4: Demographic characteristics of included studies**

| **study** | **Country** | **Interventions, sample sizes(male), mean age** | **stroke grade or site; categories of stroke** | **Months after stroke** | **Duration** | **Outcomes** | **Parameter** |
| --- | --- | --- | --- | --- | --- | --- | --- |
| **Dorian et al, 2018** | America | EC-BGT, 8(4), 53.80 BGT, 8(2), 66.60 | BBS score lower than 45/56; NR | 0.27 | 2 weeks | BBS, 5MWT, Sensory Organization Test | TB, dSSB, sSSB |
| **Loris et al, 2020** | Italy | BWS-TT--ECA, 21(15), 66.20  BWS-TT, 21(16), 66.90 CON, 21(14), 65.60 | Unilateral stroke;  Ischemic | 0.55 | 6 weeks | Tinetti test, Motricity Index, Static gait balance | TB, dSSB, sSSB |
| **Marcia et al, 2018** | Brazil | RA-GT, 5(2), 44.40 BGT, 8(6), 56.40 | Stroke survivors with ataxia; NR | 9.18 | 5 months | BBS, TUG | TB, PB |
| **Addie et al, 2014** | South Carolina | BWS-TT, 23(14), 61.40 BGT, 20(16), 60.40 | Unilateral hemiplegia; NR | 37.20 | 2 weeks | BBS, DGI, TUG | TB, dSSB, sSSB, PB |
| **Christoph et al, 2012** | Germany | TT, 18(14), 68.60 CON, 18(15), 68.70 | At least 1 clinical sign for paresis, spasticity or circumduction of the affected leg while walking, and the ability to walk on the treadmill at ≥0.3 km/h for 3 minutes with handrail support; Ischemic | 65.10 | 3 months | BBS, 10MWT | TB, dSSB |
| **Jin et al, 2015** | Korea | TT-ECA, 9(4), 51.80 BGT-ECA, 10(6), 55.00 | Walk for 10 minutes or longer on a treadmill; NR | 11.40 | 3 weeks | WS, TUG | dSSB, PB |
| **Nan-Hyang et al, 2020** | Korea | AQE-BGT, 10(5), 65.20 BGT, 11(9), 61.40 | Severe hemiplegic stroke, in levels 1 or 2 of the FAC test; NR | 23.75 | 12 weeks | WS, PASS | dSSB, sSSB |
| **Marilyn et al, 2013** | Canada | BWS-TT, 24(15), 61.50 CON, 26(14), 59.00 | Able to walk 5 m; Ischemic | 0.78 | 6 weeks | BBS, WS | TB, dSSB |
| **Amy et al, 2020** | UK | RA-GT, 16(14), 59.60 CON, 18(14), 65.10 | FAC score between 2 and 5; NR | 31.50 | 10 weeks | BBS,  DGI, TUG | TB, dSSB, PB |
| **Ki et al, 2014** | Korea | VR-GT, 15(7), 65.90 TT, 15(8), 63.50 | Ability to walk 10 m, Korean version of MMSE >24; Ischemic | 30.08 | 6 weeks | BBS, WS, postural sway velocity moment, TUG | TB, dSSB, sSSB, PB |
| **Ilona et al, 2021** | Netherlands | VR-GT, 28(18), 65.00 BGT, 24(18), 61.00 | FAC score ≥ 3; NR | 2.50 | 6 weeks | Mini-BESTest, TUG | dSSB, PB |
| **Hyun-Ju et al, 2011** | Korea | BGT, 13(7), 59.40 CON, 12(5), 56.90 | A walking speed of <0.7 m/s, MMSE score >25; NR | 28.38 | 4 weeks | ABC Scale,  10MWT, Community walk test | TB, dSSB, PB |
| **Raymond et al, 2006** | Hong Kong | BWS-TT-ECA, 15(10), 61.80 BWS-TT, 15(9), 66.10 BGT, 20(12), 71.40 | MMSE score >21, FAC score <3; Ischemic or hemorrhagic | 1.36 | 4 weeks | BBS, 5MWT | TB, dSSB |
| **Nara et al, 2015** | Korea | VR-GT, 10(NR), NR CON, 7(NR), NR | NR; Ischemic | 6.00 | 4 weeks | Postural sway path length | sSSB |
| **Yeon-Gyo et al, 2018** | Korea | RA-GT, 18(11), 48.30 BGT, 16(6), 68.60 | MMSE score ≥10; Ischemic or hemorrhagic | 13.58 | 4 weeks | BBS, 10MWT | TB, dSSB |
| **Anna et al, 2020** | Poland | RA-GT, 23(21), 69.00 CON, 13(12), 70.00 | Spasticity of muscles of the lower limbs ≤3 according to the Modified Ashworth Scale，Disproportion in the length of lower limbs ≤2 cm; Ischemic | 8.25 | 4 weeks | COP average velocity | sSSB |
| **Tomoyuki et al, 2020** | Japan | RA-GT, 8(6), 66.10 TT, 11(9), 65.00 | Modified Ashworth scale ≤ 2, MMSE score ≥ 23; Ischemic | 181.20 | 4 weeks | WS, TUG | dSSB, PB |
| **Yong-Jun et al, 2018** | Korea | BGT-ECA1, 11(8), 64.60 BGT-ECA2, 10(7), 63.00 BGT, 10(6), 61.80 | Between 3 and 5 on the Brunnström scale; NR | 73.05 | 6 weeks | 10MWT, COL path velocity (eyes closed), TUG | dSSB, sSSB, PB |
| **Akiyoshi et al, 2010** | Japan | EC-BGT, 10(4), 66.10 BWS-TT, 11(8), 71.10 CON, 12(5), 66.90 | Functional Independence Measure Locomotion score of 5 or lower; Hemorrhagic | 0.46 | 3 weeks | BBS, WS | TB, dSSB |
| **Kyung et al, 2017** | Korea | EC-BGT, 15(11), 48.30 TT, 15(7), 50.70 | FAC scores exceeding four and five points, MMSE score > 24; Hemorrhagic | 11.10 | 4 weeks | DGI | dSSB |
| **JaYoung et al, 2018** | Korea | RA-GT, 25(20), 57.70 CON, 23(13), 60.40 | FAC score ≥ 2; Ischemic or hemorrhagic | 2.30 | 3 weeks | BBS, 10MWT | TB, dSSB |
| **Young-Hyeon et al, 2014** | Korea | RA-GT-ECA, 10(6), 45.40 RA-GT, 10(7), 52.00 | Korean version of MMSE score > 21; Ischemic or hemorrhagic | 10.65 | 5 weeks | BBS, WS, TUG | TB, dSSB, PB |
| **Dae-Hyouk et al, 2016** | Korea | RA-GT, 9(5), 53.56 TT, 9(4), 53.70 | A gait speed over 0.4 m/s, possible independent gait over 10 m, MMSE score of 24 or higher; NR | 12.06 | 4 weeks | BBS, WS | TB, dSSB |
| **Hyung-Kyu et al, 2011** | Korea | TT-ECA, 10(6), 55.90 TT, 10(4), 56.30 CON, 10(6), 56.10 | MMSE score ≥ 21, Brunnstrum stage > 4; Ischemic | 13.80 | 4 weeks | 10MWT, TUG | dSSB, PB |
| **Yong-Wook et al, 2015** | Korea | EC-BGT, 18(13), 53.40 TT, 19(12), 51.80 | Korean version of MMSE score ≥ 24; NR | 31.90 | 4 weeks | WS, Biodex Medical Systems overall | dSSB, sSSB |
| **Wan- Yun et al, 2021** | Taiwan | BGT-ECA, 15(12), 57.00 CON, 15(11), 66.10 | Walk independently for more than 20 m; NR | 71.95 | 4 weeks | BBS, WS, TUG | TB, dSSB, PB |
| **Wan-Yun et al, 2021** | Taiwan | BGT, 12(9), 53.70 CON, 12(9), 63.30 | MMSE ≥ 24，ability to walk 15 m independently; Hemorrhagic | 63.08 | 12 weeks | WS, PASS, TUG | dSSB, sSSB, PB |
| **Maijke et al, 2020** | The Netherlands | BGT-ECA, 19(15), 57.00 BGT, 18(14), 58.70 | Unilateral stroke; NR | 0.60 | 10 weeks | 10MWT | dSSB |
| **Yeon-Gyo et al, 2020** | Korea | RA-GT, 18(8), 57.30 BGT, 20(14), 60.00 | Hemiplegia or hemiparesis after stroke; NR | 19.11 | 4 weeks | BBS, 10MWT | dSSB |
| **Chu-Ling et al, 2007** | Taiwan | BWS-TT, 7(3), 57.30 CON, 7(6), 56.10 | Unilateral stroke，ability to walk at least 10 m; NR | 23.58 | 4 weeks | WS | dSSB |
| **Aleksandra et al, 2014** | Serbia | BWS-TT, 11(9), 57.30 CON, 11(9), 58.10 | No severe cognitive or communication impairment; NR | 1.24 | 4 weeks | BBS, 10MWT | TB, dSSB |
| **Stein et al, 2014** | America | RA-GT, 12(10), 57.60 BGT, 12(7), 56.60 | Required to be independent in household ambulation; Ischemic or hemorrhagic | 68.80 | 6 weeks | BBS,  10MWT, Romberg (eyes closed), TUG | TB, dSSB, sSSB, PB |
| **Kyoung-Sim et al, 2019** | Korea | BGT-ECA, 10(7), 57.10 BGT, 10(7), 56.30 | FAC score 2–3; NR | 6.65 | 4 weeks | Trunk Impairment Scale , TUG | TB, dSSB, sSSB, PB |
| **Soonhyun et al, 2018** | Korea | BGT-ECA, 23(13), 56.00 CON, 21(11), 54.90 | Korean version of MMSE score ＞ 21; NR | 14.26 | 6 weeks | BBS, WS, TUG | TB, dSSB, PB |
| **Roberto et al, 2014** | Spain | VR-GT, 10(4), 55.00 CON, 10(5), 58.30 | MMSE score > 23; NR | 16.59 | 4 weeks | BBS, 10MWT | TB, dSSB |
| **Yuri et al, 2014** | Korea | BGT-ECA, 10(6), 59.80 BGT, 10(6), 63.00 | MMSE score ≥ 20, Brunnstrom stage III or IV in the proximal and distal parts of the lower extremit; Ischemic or hemorrhagic | 14.6 | 6 weeks | BBS, WS | TB, dSSB |
| **Broderick et al, 2018** | Ireland | TT-ECA, 16(11), 61.20 TT, 15(14), 67.06 | MMSE score ≥ 25; Ischemic | 54.70 | 4 weeks | 10MWT | dSSB |
| **Nataša et al, 2016** | Slovenia | RA-GT, 9(4), 52.00 CON, 10(7), 60.00 | FAC score 0-2, Postural Assessment Scale for Stroke of at least 4; Ischemic or hemorrhagic | 3.48 | 3 weeks | BBS, WS | TB, dSSB |
| **Kelly et al, 2009** | America | RA-GT, 8(6), 58.60 BWS-TT, 8(7), 55.10 | At least unlimited household ambulators; NR | 40.30 | 4 weeks | BBS, WS | TB, dSSB |
| **Mania et al, 2015** | Iran | BGT-ECA, 14(9), 56.90 CON, 14(10), 53.40 | Stance asymmetry toward the non-paretic side (patients born > 53%, body weight on the non-paretic side); NR | 37.32 | 6 weeks | WS | dSSB |
| **Ling-Fung et al, 2018** | Hong Kong | RA-GT, 9(6), 54.20 BGT, 10(7), 61.20 | FAC ≥ 4, BBS ≥ 40; Ischemic | 62.40 | 6 months | BBS, WS | TB, dSSB |
| **NaRi et al, 2018** | Korea | RA-GT, 18(10), 63.60 CON, 18(9), 64.30 | Burke Lateropulsion Scale score over 2 points; NR | 1.00 | 3 weeks | BBS, PASS | TB, sSSB |
| **Stanley et al, 2011** | America | RA-GT, 10(7), 60.00 CON, 10(7), 60.00 | Severe dementia or other cognitive impairment preventing meaningful communication; NR | 2.30 | 6-8 weeks | Tinetti balance assessment, 8MWT | TB, dSSB |
| **Eun et al, 2016** | Korea | RA-GT, 30(17), 67.90 CON, 26(15), 63.20 | FAC score < 2; Ischemic or hemorrhagic | 0.66 | 4 weeks | BBS, FAC | TB, dSSB |
| **Arun et al, 2018** | America | RA-GT, 25(17), 61.60 BGT, 25(16), 59.50 | MMSE score > 17, initial walking speed 0.4–0.8 m/s, able to sit unsupported for 30 seconds, able to walk at least 10 meters with maximum 1-person assist; NR | 75.00 | 6-8 weeks | BBS, 10MWT | TB, dSSB |
| **Yu-Rong et al, 2015** | China | BWS-TT, 12(2), 60.00 BGT, 12(9), 60.80 | MMSE score ≥ 27, average modified Ashworth scale score at hip, knee, and ankle ≤ 2; NR | 1.62 | 3 weeks | Brunel Balance Assessment, WS | TB, dSSB |
| **Kyeong et al, 2021** | Korea | RA-GT, 18(12), 61.20 CON, 18(9), 60.40 | FAC score ≥ 2; NR | 3.50 | 3 weeks | BBS, WS | TB, dSSB |
| **Seok et al, 2014** | Korea | AQE-BGT, 10(6), 61.80 CON, 10(5), 60.60 | Move at least 10 m; NR | 15.00 | 4 weeks | DB ability | dSSB |
| **Ken-Wei et al, 2021** | Taiwan | EC-BGT, 8(5), 54.40 CON, 8(6), 52.40 | Brunnstrom motor stage of lower extremity equal to or greater than IV, ability to walk at least 11 m; Ischemic or hemorrhagic | 33.29 | 4 weeks | BBS, 10MWT, TUG | TB, dSSB, PB |
| **Robert et al, 2019** | Australia | RA-GT, 16(8), 63.20 CON, 20(14), 68.10 | NR; NR | 0.69 | 3 weeks | 10MWT, TUG | dSSB, PB |
| **Sinikka et al, 2005** | Finland | BWS-TT-ECA, 15(13), 53.30 BWS-TT, 15(13), 51.20 BGT, 15(11), 52.30 | NR; NR | 36.00 | 3 weeks | DB time, anteroposterior speed of COP | dSSB, sSSB |
| **Lee et al, 2013** | Korea | BWS-TT-ECA, 15(12), 52.50 BWS-TT, 15(10), 56.70 | Brunnstrom stage between 1 and 4 for the lower extremity; Ischemic or hemorrhagic | 4.04 | 4 weeks | BBS, WS, TUG | TB, dSSB, PB |
| **Jaeho et al, 2018** | Korea | VR-GT, 12(7), 55.60 RA-GT-ECA, 12(7), 56.70 CON, 16(9), 57.50 | maximum BBS score 45, minimum MMSE score 24; NR | 11.21 | 6 weeks | BBS, 10MWT, TUG | TB, dSSB, PB |
| **Yeon-Gyu et al, 2016** | Korea | DT-BGT, 15(10), 73.70 TT, 14(6), 71.40 | 4 or 5 on the FAC; NR | 9.59 | 4 weeks | BBS, 10MWT, TUG | TB, dSSB, PB |
| **Taesung et al, 2016** | Korea | TT-ECA, 15(8), 53.20 TT, 15(9), 53.50 | Minimum score of 3 on the FAC; NR | 6.47 | 4 weeks | Performance-oriented mobility assessment, 10MWT, TUG | TB, dSSB, PB |
| **Dal-Yeon et al, 2015** | Korea | TT-ECA, 15(9), 50.00 TT, 15(8), 49.50 | Walk more than 15 meters independently, scored at least 24 in the MMSE, 2^nd^ grade or lower on ankle plantar flexor response on the Modified Ashworth Scale, between the 2^nd^ and 4^th^ stages on the Brunnstrom Stages; NR | 6.45 | 4 weeks | BBS, 10MWT, TUG | TB, dSSB, PB |
| **I-Hsuan et al, 2013** | Taiwan | DT-BGT, 15(14), 53.70 TT, 15(13), 54.80 | Brunnstrom stage of affected lower extremity greater than 3; Ischemic | 30.60 | 4 weeks | BBS, WS, Sensory Organization Test | TB, dSSB, sSSB |
| **Cho et al, 2013** | Korea | VR-GT, 7(3), 64.60 TT, 7(4), 65.14 | Brunnstrom score between 1 and 4; NR | 10.01 | 6 weeks | BBS, WS, TUG | TB, dSSB, PB |
| **Yoon-Hee et al, 2017** | Korea | BGT-ECA, 12(8), 62.80 BGT, 12(8), 59.70 | Lower limb motricity between 3 and 5 on the Brunnstrom scale; NR | 70.00 | 6 weeks | 10MWT, COP path length, TUG | dSSB, sSSB, PB |
| **Sarah et al, 2018** | America | DT-BGT, 14(8), 48.90 BWS-TT, 15(7), 60.30 | Able to ambulate ≥ 14 m; Ischemic or hemorrhagic | 49.85 | 6 weeks | BBS, WS | TB, dSSB |
| **Lena et al, 2001** | Sweden | BWS-TT, 36(20), 54.00 BGT, 37(20), 56.00 | More than 14 seconds to walk 10 metres were included; NR | 0.65 | 3~19 weeks | BBS, WS | TB, dSSB |
| **Bernhard et al, 2019** | Germany | BGT-ECA, 6(1), 68.70 BGT, 6(2), 65.30 | FAC score > 3; Ischemic | 66.95 | 4 weeks | BBS, WS | TB, dSSB |
| **Yang et al, 2008** | Taiwan | VR-GT, 11(5), 55.50 TT, 9(5), 60.90 | NR; NR | 72.18 | 4 weeks | ABC scale, WS | TB, dSSB |
| **Forrester et al, 2016** | America | RA-GT, 14(9), 59.50 VR-GT, 12(7), 56.80 | Paretic ankle dorsi-flexor manual muscle test score ≥ 2 (full ROM gravity eliminated) and ≤ 4 (full ROM against gravity, moderate resistance) in dorsiflexion and/or plantarflexion, and capacity to treadmill walk ≥ 0.12 m/sec for 3 min with handrail support; Ischemic | 35.70 | 6 weeks | WS, Paretic single support center of pressure length | dSSB, sSSB |
| **Inoue et al, 2022** | Japan | RA-GT, 18(9), 61.60 CON, 20(12), 69.70 | FAC score ≥ 2; NR | 1.80 | 2 weeks | Mini-BESTest, Maximum COP movement, TUG | dSSB, sSSB, PB |
| **Choi, 2022** | Korea | RA-GT, 6(4), 52.70 TT, 6(3), 61.40 | MMSE score ≥ 24, able to walk >10 m using orthosis or mobility aids; NR | 16.55 | 6 weeks | BBS, 10MWT, TUG | TB, dSSB, PB |

10WMT, 10-Metre Walk Test; 5MWT, 5-Meter Walk Test; ABC Scale, Activities-specific Balance Confidence Scale ; AQE-BGT, Aquatic balance and gait training; BBS, Berg Balance Scale; BGT, balance and gait training; BGT-ECA, balance and gait training with external cues; BWS-TT, body weight supported treadmill training ; BWS-TT-ECA, body weight supported treadmill training with external cues; COL, center of loading; CON, Control group; COP, center of pressure; DB ability, Dynamic balance ability; DB time, Dynamic balance time; DGI, Dynamic Gait Index; dSSB, dynamic steady-state balance; DT-BGT, dual-task gait training; EC-BGT, eyes closed gait training ; FAC, Functional ambulatory category scale; FAC , Functional Ambulation Category; MMSE, Mini-Mental State Examination; NR, Not reported; PASS, Postural Assessment Scale for Stroke; PB, proactive balance; RA-GT, robotic-assisted gait training; RA-GT-ECA, robotic-assisted gait training with external cues; RB, reactive balance; sSSB, static steady state balance; TB, balance test battery; TCT, Trunk Control Test; TT, treadmill gait training ; TT-ECA, treadmill gait training with external cues; TUG, Timed Up and Go Test; VR-GT, virtual reality gait training ; WS, Walking Speed


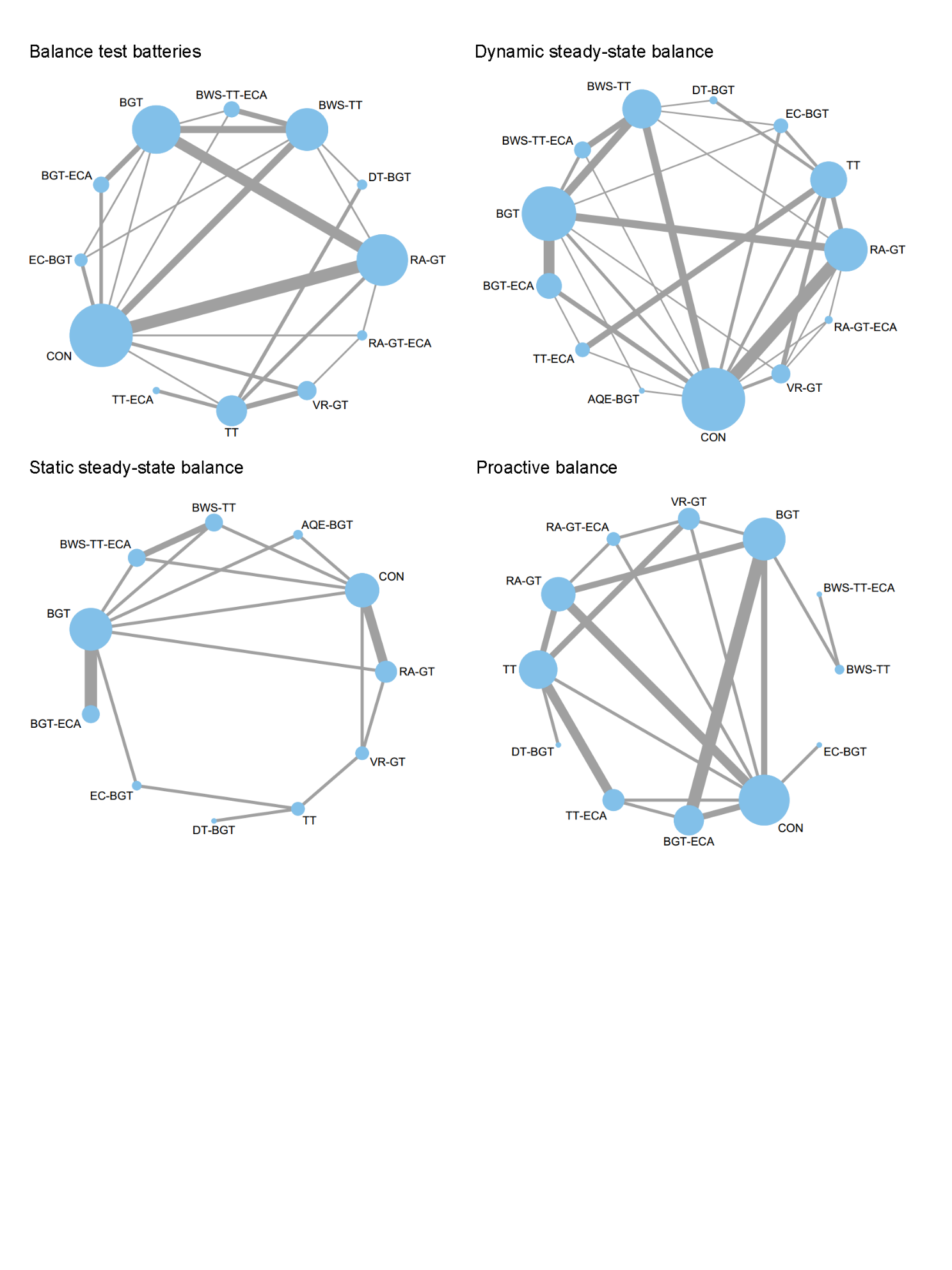
**Appendix 5: Network graphs of outcomes**

**Figure. S1 Network graphs for balance test batteries, dynamic steady-state balance, static steady-state balance, and proactive balance.** The size of the nodes corresponds to the number of participants assigned to each treatment. Treatments with direct comparisons are linked with a line; its thickness corresponds to the number of trials evaluating the comparison. AQE-BGT, Aquatic balance and gait training; BGT, balance and gait training; BGT-ECA, balance and gait training with external cues; BWS-TT, body weight supported treadmill training; BWS-TT-ECA, body weight supported treadmill training with external cues; CON, Control group; DT-BGT, dual-task gait training; EC-BGT, eyes closed gait training; RA-GT, robotic-assisted gait training; RA-GT-ECA, robotic-assisted gait training with external cues; TT, treadmill gait training; TT-ECA, treadmill gait training with external cues; VR-GT, virtual reality gait training.

**Appendix 6: Risk of bias**

**6.1 Risk of bias graph**

**Figure. S2** Risk of bias graph for balance test batteries

**Figure. S3** Risk of bias graph for dynamic steady-state balance

**Figure. S5** Risk of bias graph for proactive balance

**Figure. S4** Risk of bias graph for static steady-state balance

**6.2 Risk of bias summary**

**Figure. S7** Risk of bias summary for dynamic steady-state balance

**Figure. S6** Risk of bias summary for balance test batteries

**Figure. S8** Risk of bias summary for proactive balance

**Figure. S9** Risk of bias summary for static steady-state balance

**Appendix 7: Forest plot of outcomes**

Significant difference

Non-significant difference

Favors CON Favors Treatments

**Figure. S10 Forest plot based on the balance test battery outcome, and the efficacy of varied exercises compared with the named control group.** BGT, balance and gait training; BGT-ECA, balance and gait training with external cues; BWS-TT, body weight supported treadmill training; BWS-TT-ECA, body weight supported treadmill training with external cues; CI, Confidence interval; CON, Control group; DT-BGT, dual-task gait training; EC-BGT, eyes closed gait training; RA-GT, robotic-assisted gait training; RA-GT-ECA, robotic-assisted gait training with external cues; TT, treadmill gait training; TT-ECA, treadmill gait training with external cues; VR-GT, virtual reality gait training.

Significant difference

Non-significant difference

Favors CON Favors Treatments

**Figure. S11 Forest plot based on the dynamic steady-state balance outcome, and the efficacy of varied exercises compared with the named control group.** AQE-BGT, Aquatic balance and gait training; BGT, balance and gait training; BGT-ECA, balance and gait training with external cues; BWS-TT, body weight supported treadmill training; BWS-TT-ECA, body weight supported treadmill training with external cues; CI, Confidence interval; CON, Control group; DT-BGT, dual-task gait training; EC-BGT, eyes closed gait training; RA-GT, robotic-assisted gait training; RA-GT-ECA, robotic-assisted gait training with external cues; TT, treadmill gait training; TT-ECA, treadmill gait training with external cues; VR-GT, virtual reality gait training.

Favors CON Favors Treatments

Significant difference

Non-significant difference

**Figure. S12 Forest plot based on the static steady state balance, and the efficacy of varied exercises compared with the named control group.** AQE-BGT, Aquatic balance and gait training; BGT, balance and gait training; BGT-ECA, balance and gait training with external cues; BWS-TT, body weight supported treadmill training ; BWS-TT-ECA, body weight supported treadmill training with external cues; CI, Confidence interval; CON, Control group; DT-BGT, dual-task gait training; EC-BGT, eyes closed gait training; RA-GT, robotic-assisted gait training; RA-GT-ECA, robotic-assisted gait training with external cues; TT, treadmill gait training; VR-GT, virtual reality gait training.

Significant difference

Non-significant difference

Favors CON Favors Treatments

**Figure. S13 Forest plot based on the proactive balance outcome, and the efficacy of varied exercises compared with the named control group.** BGT, balance and gait training; BGT-ECA, balance and gait training with external cues; BWS-TT, body weight supported treadmill training; BWS-TT-ECA, body weight supported treadmill training with external cues; CI, Confidence interval; CON, Control group; DT-BGT, dual-task gait training; EC-BGT, eyes closed gait training; RA-GT, robotic-assisted gait training; RA-GT-ECA, robotic-assisted gait training with external cues; TT, treadmill gait training; TT-ECA, treadmill gait training with external cues; VR-GT, virtual reality gait training.

**Appendix 8: Network Meta-Regression**

***Changes in heterogeneity***

Below we present the results from the changes in heterogeneity in each meta-regression model.

| Covariate | Shared beta  (median and 95% CI) | Heterogeneity standard deviation (median and 95% CI) | % of variance explained |
| --- | --- | --- | --- |
| **Balance test batteries** | | | |
| None | **-** | 0.565 (0.37; 0.80) | - |
| Publish Year | -0.29 (-1.07; 0.45) | 0.576 (0.41; 0.87) | 4.2% |
| Mean Age | -0.00 (-0.81; 0.78) | 0.579 (0.39; 0.85) | 5.3% |
| Years of Diagnosis | 0.36 (-0.40; 1.18) | 0.568 (0.38; 0.81) | 1.1% |
| Percentage Male | 0.08 (-0.63; 0.84) | 0.577 (0.39; 0.83) | 4.4% |
| Exercise Period | 0.46 (-0.43; 1.37) | 0.566 (0.38; 0.83) | 0.4% |
| Exercise Frequency | -0.31 (-0.98; 0.32) | 0.568 (0.38; 0.81) | 1.3% |
| **Dynamic steady-state balance** | | | |
| None | **-** | 0.380 (0.26; 0.53) | - |
| Publish Year | -0.13 (-0.63; 0.34) | 0.384 (0.25; 0.55) | 2.0% |
| Mean Age | 0.03 (-0.49; 0.54) | 0.387 (0.26; 0.55) | 3.9% |
| Years of Diagnosis | 0.52 (-0.19; 1.22) | 0.385 (0.26; 0.54) | 2.7% |
| Percentage Male | -0.08 (-0.59; 0.46) | 0.388 (0.26; 0.55) | 4.4% |
| Exercise Period | 0.25 (-0.17; 0.69) | 0.384(0.26; 0.54) | 2.4% |
| Exercise Frequency | -0.36 (-0.78; 0.07) | 0.385 (0.27; 0.54) | 2.6% |
| **Static steady-state balance** | | | |
| None | **-** | 0.197 (0.01; 0.59) | - |
| Publish Year | -0.90 (-2.53; 0.46) | 0.211 (0.02; 0.58) | 15.6% |
| Mean Age | 0.28 (-0.79; 1.28) | 0.198 (0.01; 0.61) | 1.3% |
| Years of Diagnosis | -0.45 (-1.31; 0.44) | 0.195 (0.01; 0.60) | -1.6% |
| Percentage Male | 0.06 (-0.63; 0.74) | 0.213 (0.00; 0.64) | 17.9% |
| Exercise Period | -0.33 (-1.11; 0.43) | 0.224 (0.02; 0.63) | 29.7% |
| Exercise Frequency | 0.26 (-0.42; 0.87) | 0.208 (0.01; 0.63) | 12.4% |
| **Proactive balance** | | | |
| None | **-** | 0.369 (0.09; 0.75) | - |
| Publish Year | -0.45 (-1.12; 0.22) | 0.343 (0.06; 0.72) | -13.6% |
| Mean Age | -0.19(-1.54; 0.97) | 0.375 (0.04; 0.79) | 3.6% |
| Years of Diagnosis | -0.81 (-2.03; 0.43) | 0.339 (0.06; 0.70) | -15.2% |
| Percentage Male | -0.49 (-1.08; 0.08) | 0.319 (0.04; 0.68) | -25.7% |
| Exercise Period | -0.41 (-1.28; 0.46) | 0.368 (0.08; 0.74) | -0.4% |
| Exercise Frequency | -0.13 (-0.82; 0.55) | 0.384 (0.06; 0.79) | 8.2% |

CI: Confidence Interval; *: Significant influence factors, 95% CI does not contain zero

**Appendix 9: ‘Hot spots’ of inconsistency**

**9.1 ‘Hot spots’ of inconsistency: balance test batteries**

| Random effects model | |
| --- | --- |
| k | - Number of studies providing direct evidence |
| prop | - Direct evidence proportion |
| nma | - Estimated treatment effect (SMD) in network meta- analysis |
| direct | - Estimated treatment effect (SMD) derived from direct evidence |
| indir. | - Estimated treatment effect (SMD) derived from indirect evidence |
| Diff | - Difference between direct and indirect treatment estimates |
| z | - z-value of test for disagreement (direct versus indirect) |
| P | - p-value of test for disagreement (direct versus indirect) |

| Comparison | k | prop | nma | direct | indir. | Diff | z | p-value |
| --- | --- | --- | --- | --- | --- | --- | --- | --- |
| BGT vs BGT-ECA | 3 | 0.59 | -0.36 | -0.46 | -0.20 | -0.25 | -0.38 | 0.7040 |
| BGT vs BWS-TT | 4 | 0.57 | -0.16 | -0.13 | -0.20 | 0.07 | 0.14 | 0.8876 |
| BGT vs BWS-TT-ECA | 1 | 0.34 | -0.90 | -0.50 | -1.11 | 0.61 | 0.76 | 0.4466 |
| BGT vs CON | 1 | 0.11 | 0.1 | 1.32 | -0.05 | 1.37 | 1.84 | 0.0655 |
| BGT vs DT-BGT | 0 | 0 | -0.13 | . | -0.13 |  |  | . |
| BGT vs EC-BGT | 1 | 0.32 | -0.64 | -0.74 | -0.59 | -0.15 | -0.16 | 0.8728 |
| BGT vs RA-GT | 6 | 0.62 | -0.32 | -0.54 | 0.04 | -0.57 | -1.27 | 0.2037 |
| BGT vs RA-GT-ECA | 0 | 0 | -0.62 | . | -0.62 |  |  | . |
| BGT vs TT | 0 | 0 | 0.51 | . | 0.51 |  |  | . |
| BGT vs TT-ECA | 0 | 0 | -0.23 | . | -0.23 |  |  | . |
| BGT vs VR-GT | 0 | 0 | -1.27 | . | -1.27 |  |  | . |
| BGT-ECA vs BWS-TT | 0 | 0 | 0.2 | . | 0.2 |  |  | . |
| BGT-ECA vs BWS-TT-ECA | 0 | 0 | -0.55 | . | -0.55 |  |  | . |
| BGT-ECA vs CON | 2 | 0.54 | 0.46 | 0.34 | 0.59 | -0.25 | -0.38 | 0.7040 |
| BGT-ECA vs DT-BGT | 0 | 0 | 0.22 | . | 0.22 |  |  | . |
| BGT-ECA vs EC-BGT | 0 | 0 | -0.29 | . | -0.29 |  |  | . |
| BGT-ECA vs RA-GT | 0 | 0 | 0.04 | . | 0.04 |  |  | . |
| BGT-ECA vs RA-GT-ECA | 0 | 0 | -0.27 | . | -0.27 |  |  | . |
| BGT-ECA vs TT | 0 | 0 | 0.86 | . | 0.86 |  |  | . |
| BGT-ECA vs TT-ECA | 0 | 0 | 0.13 | . | 0.13 |  |  | . |
| BGT-ECA vs VR-GT | 0 | 0 | -0.91 | . | -0.91 |  |  | . |
| BWS-TT vs BWS-TT-ECA | 3 | 0.86 | -0.74 | -0.79 | -0.46 | -0.33 | -0.32 | 0.7495 |
| BWS-TT vs CON | 4 | 0.53 | 0.26 | 0.24 | 0.29 | -0.05 | -0.1 | 0.9200 |
| BWS-TT vs DT-BGT | 1 | 0.49 | 0.03 | 0.06 | -0.00 | 0.06 | 0.07 | 0.9463 |
| BWS-TT vs EC-BGT | 1 | 0.38 | -0.48 | -0.49 | -0.47 | -0.02 | -0.02 | 0.9841 |
| BWS-TT vs RA-GT | 1 | 0.12 | -0.16 | 0.36 | -0.23 | 0.59 | 0.75 | 0.4541 |
| BWS-TT vs RA-GT-ECA | 0 | 0 | -0.46 | . | -0.46 |  |  | . |
| BWS-TT vs TT | 0 | 0 | 0.67 | . | 0.67 |  |  | . |
| BWS-TT vs TT-ECA | 0 | 0 | -0.07 | . | -0.07 |  |  | . |
| BWS-TT vs VR-GT | 0 | 0 | -1.11 | . | -1.11 |  |  | . |
| BWS-TT-ECA vs CON | 1 | 0.37 | 1 | 1.05 | 0.97 | 0.08 | 0.1 | 0.9190 |
| BWS-TT-ECA vs DT-BGT | 0 | 0 | 0.77 | . | 0.77 |  |  | . |
| BWS-TT-ECA vs EC-BGT | 0 | 0 | 0.26 | . | 0.26 |  |  | . |
| BWS-TT-ECA vs RA-GT | 0 | 0 | 0.58 | . | 0.58 |  |  | . |
| BWS-TT-ECA vs RA-GT-ECA | 0 | 0 | 0.28 | . | 0.28 |  |  | . |
| BWS-TT-ECA vs TT | 0 | 0 | 1.41 | . | 1.41 |  |  | . |
| BWS-TT-ECA vs TT-ECA | 0 | 0 | 0.67 | . | 0.67 |  |  | . |
| BWS-TT-ECA vs VR-GT | 0 | 0 | -0.36 | . | -0.36 |  |  | . |
| DT-BGT vs CON | 0 | 0 | 0.23 | . | 0.23 |  |  | . |
| EC-BGT vs CON | 2 | 0.65 | 0.74 | 0.7 | 0.81 | -0.11 | -0.13 | 0.8991 |
| RA-GT vs CON | 7 | 0.64 | 0.42 | 0.21 | 0.79 | -0.58 | -1.42 | 0.1553 |
| RA-GT-ECA vs CON | 1 | 0.49 | 0.72 | 0.98 | 0.47 | 0.51 | 0.54 | 0.5867 |
| TT vs CON | 1 | 0.3 | -0.41 | 0.28 | -0.70 | 0.98 | 1.29 | 0.1955 |
| TT-ECA vs CON | 0 | 0 | 0.33 | . | 0.33 |  |  | . |
| VR-GT vs CON | 2 | 0.58 | 1.37 | 1.32 | 1.43 | -0.11 | -0.14 | 0.8866 |
| DT-BGT vs EC-BGT | 0 | 0 | -0.51 | . | -0.51 |  |  | . |
| DT-BGT vs RA-GT | 0 | 0 | -0.19 | . | -0.19 |  |  | . |
| DT-BGT vs RA-GT-ECA | 0 | 0 | -0.49 | . | -0.49 |  |  | . |
| DT-BGT vs TT | 2 | 0.74 | 0.64 | 0.66 | 0.6 | 0.06 | 0.07 | 0.9463 |
| DT-BGT vs TT-ECA | 0 | 0 | -0.10 | . | -0.10 |  |  | . |
| DT-BGT vs VR-GT | 0 | 0 | -1.13 | . | -1.13 |  |  | . |
| EC-BGT vs RA-GT | 0 | 0 | 0.32 | . | 0.32 |  |  | . |
| EC-BGT vs RA-GT-ECA | 0 | 0 | 0.02 | . | 0.02 |  |  | . |
| EC-BGT vs TT | 0 | 0 | 1.15 | . | 1.15 |  |  | . |
| EC-BGT vs TT-ECA | 0 | 0 | 0.41 | . | 0.41 |  |  | . |
| EC-BGT vs VR-GT | 0 | 0 | -0.63 | . | -0.63 |  |  | . |
| RA-GT vs RA-GT-ECA | 1 | 0.45 | -0.30 | -0.16 | -0.42 | 0.27 | 0.28 | 0.7788 |
| RA-GT vs TT | 2 | 0.34 | 0.83 | 1.31 | 0.57 | 0.74 | 0.98 | 0.3258 |
| RA-GT vs TT-ECA | 0 | 0 | 0.09 | . | 0.09 |  |  | . |
| RA-GT vs VR-GT | 0 | 0 | -0.95 | . | -0.95 |  |  | . |
| RA-GT-ECA vs TT | 0 | 0 | 1.13 | . | 1.13 |  |  | . |
| RA-GT-ECA vs TT-ECA | 0 | 0 | 0.39 | . | 0.39 |  |  | . |
| RA-GT-ECA vs VR-GT | 1 | 0.59 | -0.64 | -0.71 | -0.54 | -0.17 | -0.16 | 0.8733 |
| TT vs TT-ECA | 2 | 1 | -0.74 | -0.74 | . |  |  | . |
| TT vs VR-GT | 3 | 0.65 | -1.78 | -1.85 | -1.65 | -0.2 | -0.25 | 0.7987 |
| TT-ECA vs VR-GT | 0 | 0 | -1.04 | . | -1.04 |  |  | . |

BGT, balance and gait training; BGT-ECA, balance and gait training with external cues; BWS-TT, body weight supported treadmill training; BWS-TT-ECA, body weight supported treadmill training with external cues; CON, Control group; DT-BGT, dual-task gait training; EC-BGT, eyes closed gait training; RA-GT, robotic-assisted gait training; RA-GT-ECA, robotic-assisted gait training with external cues; TT, treadmill gait training; TT-ECA, treadmill gait training with external cues; VR-GT, virtual reality gait training

**9.2 ‘Hot spots’ of inconsistency: dynamic steady-state balance**

| Random effects model | |
| --- | --- |
| k | - Number of studies providing direct evidence |
| prop | - Direct evidence proportion |
| nma | - Estimated treatment effect (SMD) in network meta- analysis |
| direct | - Estimated treatment effect (SMD) derived from direct evidence |
| indir. | - Estimated treatment effect (SMD) derived from indirect evidence |
| Diff | - Difference between direct and indirect treatment estimates |
| z | - z-value of test for disagreement (direct versus indirect) |
| P | - p-value of test for disagreement (direct versus indirect) |

| Comparison | k | prop | nma | direct | indir. | Diff | z | p-value |
| --- | --- | --- | --- | --- | --- | --- | --- | --- |
| AQE-BGT vs BGT | 1 | 0.53 | 0.41 | -0.06 | -0.28 | 0.22 | 0.27 | 0.7854 |
| AQE-BGT vs BGT-ECA | 0 | 0 | -0.35 | . | -0.35 | . | . | . |
| AQE-BGT vs BWS-TT | 0 | 0 | -0.17 | . | -0.17 | . | . | . |
| AQE-BGT vs BWS-TT-ECA | 0 | 0 | 0 | . | 0 | . | . | . |
| AQE-BGT vs CON | 1 | 0.51 | -0.13 | 0.16 | 0.38 | -0.22 | -0.27 | 0.7854 |
| AQE-BGT vs DT-BGT | 0 | 0 | 0.33 | . | 0.33 | . | . | . |
| AQE-BGT vs EC-BGT | 0 | 0 | 0 | . | 0 | . | . | . |
| AQE-BGT vs RA-GT | 0 | 0 | -0.11 | . | -0.11 | . | . | . |
| AQE-BGT vs RA-GT-ECA | 0 | 0 | -0.15 | . | -0.15 | . | . | . |
| AQE-BGT vs TT | 0 | 0 | 0.18 | . | 0.18 | . | . | . |
| AQE-BGT vs TT-ECA | 0 | 0 | 0.57 | . | 0.57 | . | . | . |
| AQE-BGT vs VR-GT | 0 | 0 | -0.22 | . | -0.22 | . | . | . |
| BGT vs BGT-ECA | 7 | 0.68 | -0.43 | -0.15 | -0.02 | -0.13 | -0.34 | 0.7373 |
| BGT vs BWS-TT | 5 | 0.58 | -0.22 | -0.61 | 0.1 | -0.71 | -2.09 | 0.0365 |
| BGT vs BWS-TT-ECA | 2 | 0.49 | -0.23 | -0.68 | -0.8 | 0.12 | 0.24 | 0.8073 |
| BGT vs CON | 2 | 0.13 | 0.54 | 1.37 | 0.29 | 1.08 | 2.39 | 0.0166 |
| BGT vs DT-BGT | 0 | 0 | -0.38 | . | -0.38 | . | . | . |
| BGT vs EC-BGT | 1 | 0.18 | -0.74 | -1.25 | -0.38 | -0.86 | -1.17 | 0.2401 |
| BGT vs RA-GT | 5 | 0.49 | 0.74 | -0.11 | -0.16 | 0.05 | 0.17 | 0.8671 |
| BGT vs RA-GT-ECA | 0 | 0 | 0.56 | . | 0.56 | . | . | . |
| BGT vs TT | 0 | 0 | 0.97 | . | 0.97 | . | . | . |
| BGT vs TT-ECA | 0 | 0 | 1.18 | . | 1.18 | . | . | . |
| BGT vs VR-GT | 1 | 0.28 | -0.98 | -0.02 | -0.48 | 0.46 | 0.87 | 0.3861 |
| BGT-ECA vs BWS-TT | 0 | 0 | 0.97 | . | 0.97 | . | . | . |
| BGT-ECA vs BWS-TT-ECA | 0 | 0 | -0.44 | . | -0.44 | . | . | . |
| BGT-ECA vs CON | 3 | 0.44 | -0.79 | 0.59 | 0.51 | 0.08 | 0.2 | 0.8413 |
| BGT-ECA vs DT-BGT | 0 | 0 | 0.79 | . | 0.79 | . | . | . |
| BGT-ECA vs EC-BGT | 0 | 0 | 0.98 | . | 0.98 | . | . | . |
| BGT-ECA vs RA-GT | 0 | 0 | 0.57 | . | 0.57 | . | . | . |
| BGT-ECA vs RA-GT-ECA | 0 | 0 | -0.54 | . | -0.54 | . | . | . |
| BGT-ECA vs TT | 0 | 0 | 0.43 | . | 0.43 | . | . | . |
| BGT-ECA vs TT-ECA | 1 | 0.27 | -0.27 | -0.95 | -0.25 | -0.7 | -0.99 | 0.3213 |
| BGT-ECA vs VR-GT | 0 | 0 | -0.48 | . | -0.48 | . | . | . |
| BWS-TT vs BWS-TT-ECA | 4 | 0.85 | -0.29 | -0.43 | -0.42 | -0.02 | 0.03 | 0.9773 |
| BWS-TT vs CON | 5 | 0.5 | -0.06 | 0.7 | 0.78 | -0.08 | -0.23 | 0.8192 |
| BWS-TT vs DT-BGT | 1 | 0.43 | -0.70 | -0.01 | 0.73 | 0.73 | -1.09 | 0.2746 |
| BWS-TT vs EC-BGT | 1 | 0.26 | -0.30 | -0.58 | -0.1 | -0.47 | -0.72 | 0.4743 |
| BWS-TT vs RA-GT | 1 | 0.1 | -0.29 | -0.21 | 0.22 | -0.42 | -0.66 | 0.5119 |
| BWS-TT vs RA-GT-ECA | 0 | 0 | 0.27 | . | 0.27 | . | . | . |
| BWS-TT vs TT | 0 | 0 | -0.71 | . | -0.71 | . | . | . |
| BWS-TT vs TT-ECA | 0 | 0 | -0.52 | . | -0.52 | . | . | . |
| BWS-TT vs VR-GT | 0 | 0 | 0.1 | . | 0.1 | . | . | . |
| BWS-TT-ECA vs CON | 1 | 0.29 | -0.13 | 0.83 | 1.32 | -0.5 | 0.87 | 0.383 |
| BWS-TT-ECA vs DT-BGT | 0 | 0 | 0.43 | . | 0.43 | . | . | . |
| BWS-TT-ECA vs EC-BGT | 0 | 0 | -0.54 | . | -0.54 | . | . | . |
| BWS-TT-ECA vs RA-GT | 0 | 0 | -0.20 | . | -0.20 | . | . | . |
| BWS-TT-ECA vs RA-GT-ECA | 0 | 0 | -0.63 | . | -0.63 | . | . | . |
| BWS-TT-ECA vs TT | 0 | 0 | 0.21 | . | 0.21 | . | . | . |
| BWS-TT-ECA vs TT-ECA | 0 | 0 | 0.43 | . | 0.43 | . | . | . |
| BWS-TT-ECA vs VR-GT | 0 | 0 | -0.03 | . | -0.03 | . | . | . |
| DT-BGT vs CON | 0 | 0 | -0.02 | . | -0.02 | . | . | . |
| DT-BGT vs EC-BGT | 0 | 0 | 0.54 | . | 0.54 | . | . | . |
| DT-BGT vs RA-GT | 0 | 0 | -0.24 | . | -0.24 | . | . | . |
| DT-BGT vs RA-GT-ECA | 0 | 0 | 0.18 | . | 0.18 | . | . | . |
| DT-BGT vs TT | 2 | 0.71 | 0.74 | 0.12 | 0.86 | -0.74 | -1.09 | 0.2746 |
| DT-BGT vs TT-ECA | 0 | 0 | -0.23 | . | -0.23 | . | . | . |
| DT-BGT vs VR-GT | 0 | 0 | -0.04 | . | -0.04 | . | . | . |
| EC-BGT vs CON | 2 | 0.4 | 0.84 | 0.94 | 0.99 | -0.05 | -0.1 | 0.9233 |
| EC-BGT vs RA-GT | 0 | 0 | 0.2 | . | 0.2 | . | . | . |
| EC-BGT vs RA-GT-ECA | 0 | 0 | 0.61 | . | 0.61 | . | . | . |
| EC-BGT vs TT | 2 | 0.55 | 0.61 | 0.77 | 1.22 | -0.45 | -0.84 | 0.4026 |
| EC-BGT vs TT-ECA | 0 | 0 | 1.17 | . | 1.17 | . | . | . |
| EC-BGT vs VR-GT | 0 | 0 | 0.2 | . | 0.2 | . | . | . |
| RA-GT vs CON | 8 | 0.62 | 0.39 | 0.37 | 0.89 | -0.52 | -1.8 | 0.0713 |
| RA-GT vs RA-GT-ECA | 1 | 0.42 | 0.33 | -0.07 | 0.06 | -0.13 | -0.18 | 0.8575 |
| RA-GT vs TT | 3 | 0.26 | 0.64 | 1.23 | 0.33 | 0.89 | 1.85 | 0.0644 |
| RA-GT vs TT-ECA | 0 | 0 | -0.24 | . | -0.24 | . | . | . |
| RA-GT vs VR-GT | 1 | 0.19 | -0.23 | 0.41 | -0.37 | 0.78 | 1.32 | 0.1873 |
| RA-GT-ECA vs CON | 1 | 0.5 | -0.65 | 0.7 | 0.43 | 0.27 | 0.37 | 0.7149 |
| RA-GT-ECA vs TT | 0 | 0 | -0.45 | . | -0.45 | . | . | . |
| RA-GT-ECA vs TT-ECA | 0 | 0 | 0.4 | . | 0.4 | . | . | . |
| RA-GT-ECA vs VR-GT | 1 | 0.52 | 0.41 | -0.49 | 0.06 | -0.55 | -0.7 | 0.4851 |
| TT vs CON | 2 | 0.29 | -0.01 | 0.14 | -0.06 | 0.19 | 0.44 | 0.6614 |
| TT vs TT-ECA | 4 | 0.84 | 0.19 | -0.87 | -1.55 | 0.68 | 1.02 | 0.3095 |
| TT vs VR-GT | 3 | 0.49 | -0.41 | -1.02 | -0.56 | -0.46 | -0.96 | 0.3361 |
| TT-ECA vs CON | 1 | 0.23 | 0.56 | 1.12 | 0.94 | . | . | . |
| TT-ECA vs VR-GT | 0 | 0 | -0.42 | . | -0.42 | . | . | . |
| VR-GT vs CON | 2 | 0.33 | 0.19 | 1.06 | 0.65 | 0.41 | 0.84 | 0.3991 |

AQE-BGT, Aquatic balance and gait training; BGT, balance and gait training; BGT-ECA, balance and gait training with external cues; BWS-TT, body weight supported treadmill training; BWS-TT-ECA, body weight supported treadmill training with external cues; CON, Control group; DT-BGT, dual-task gait training; EC-BGT, eyes closed gait training; RA-GT, robotic-assisted gait training; RA-GT-ECA, robotic-assisted gait training with external cues; TT, treadmill gait training; TT-ECA, treadmill gait training with external cues; VR-GT, virtual reality gait training

**9.3 ‘Hot spots’ of inconsistency: static steady-state balance**

| Random effects model | |
| --- | --- |
| k | - Number of studies providing direct evidence |
| prop | - Direct evidence proportion |
| nma | - Estimated treatment effect (SMD) in network meta- analysis |
| direct | - Estimated treatment effect (SMD) derived from direct evidence |
| indir. | - Estimated treatment effect (SMD) derived from indirect evidence |
| Diff | - Difference between direct and indirect treatment estimates |
| z | - z-value of test for disagreement (direct versus indirect) |
| P | - p-value of test for disagreement (direct versus indirect) |

| Comparison | k | prop | nma | direct | indir. | Diff | z | p-value |
| --- | --- | --- | --- | --- | --- | --- | --- | --- |
| AQE-BGT vs BGT | 1 | 0.66 | 1.1 | 0.64 | 2.01 | -1.36 | -1.71 | 0.0882 |
| AQE-BGT vs BGT-ECA | 0 | 0 | 0.59 | . | 0.59 | . | . | . |
| AQE-BGT vs BWS-TT | 0 | 0 | 1.33 | . | 1.33 | . | . | . |
| AQE-BGT vs BWS-TT-ECA | 0 | 0 | 0.76 | . | 0.76 | . | . | . |
| AQE-BGT vs CON | 1 | 0.42 | 1.48 | 2.27 | 0.9 | 1.36 | 1.71 | 0.0882 |
| AQE-BGT vs DT-BGT | 0 | 0 | -0.16 | . | -0.16 | . | . | . |
| AQE-BGT vs EC-BGT | 0 | 0 | 0.28 | . | 0.28 | . | . | . |
| AQE-BGT vs RA-GT | 0 | 0 | 0.83 | . | 0.83 | . | . | . |
| AQE-BGT vs TT | 0 | 0 | 0.88 | . | 0.88 | . | . | . |
| AQE-BGT vs VR-GT | 0 | 0 | 0.57 | . | 0.57 | . | . | . |
| BGT vs BGT-ECA | 4 | 1 | -0.51 | . | . | . | . | . |
| BGT vs BWS-TT | 1 | 0.48 | 0.26 | 0.16 | 0.3 | -0.14 | -0.29 | 0.7701 |
| BGT vs BWS-TT-ECA | 1 | 0.43 | -0.34 | -0.15 | -0.48 | -0.48 | 0.66 | 0.5089 |
| BGT vs CON | 1 | 0.28 | 0.38 | -0.08 | 0.57 | -0.65 | -1.3 | 0.1935 |
| BGT vs DT-BGT | 0 | 0 | -1.26 | . | -1.26 | . | . | . |
| BGT vs EC-BGT | 1 | 0.62 | -0.82 | -0.43 | -1.45 | 1.02 | 1.21 | 0.2253 |
| BGT vs RA-GT | 1 | 0.34 | -0.27 | -0.58 | -0.11 | -0.47 | -0.88 | 0.3766 |
| BGT vs TT | 0 | 0 | -0.22 | . | -0.22 | . | . | . |
| BGT vs VR-GT | 0 | 0 | -0.53 | . | -0.53 | . | . | . |
| BGT-ECA vs BWS-TT | 0 | 0 | 0.74 | . | 0.74 | . | . | . |
| BGT-ECA vs BWS-TT-ECA | 0 | 0 | 0.17 | . | 0.17 | . | . | . |
| BGT-ECA vs CON | 0 | 0 | 0.89 | . | 0.89 | . | . | . |
| BGT-ECA vs DT-BGT | 0 | 0 | -0.75 | . | -0.75 | . | . | . |
| BGT-ECA vs EC-BGT | 0 | 0 | -0.31 | . | -0.31 | . | . | . |
| BGT-ECA vs RA-GT | 0 | 0 | 0.24 | . | 0.24 | . | . | . |
| BGT-ECA vs TT | 0 | 0 | 0.29 | . | 0.29 | . | . | . |
| BGT-ECA vs VR-GT | 0 | 0 | -0.02 | . | -0.02 | . | . | . |
| BWS-TT vs BWS-TT-ECA | 2 | 0.8 | -0.57 | -0.64 | -0.31 | -0.31 | -0.57 | 0.5702 |
| BWS-TT vs CON | 1 | 0.59 | 0.15 | -0.03 | 0.4 | -0.43 | -0.85 | 0.3973 |
| BWS-TT vs DT-BGT | 0 | 0 | -1.50 | . | -1.50 | . | . | . |
| BWS-TT vs EC-BGT | 0 | 0 | -1.05 | . | -1.05 | . | . | . |
| BWS-TT vs RA-GT | 0 | 0 | -0.50 | . | -0.50 | . | . | . |
| BWS-TT vs TT | 0 | 0 | -0.45 | . | -0.45 | . | . | . |
| BWS-TT vs VR-GT | 0 | 0 | -0.76 | . | -0.76 | . | . | . |
| BWS-TT-ECA vs CON | 1 | 0.58 | 0.72 | 1.05 | 0.26 | 0.8 | 1.51 | 0.1299 |
| BWS-TT-ECA vs DT-BGT | 0 | 0 | -0.92 | . | -0.92 | . | . | . |
| BWS-TT-ECA vs EC-BGT | 0 | 0 | -0.48 | . | -0.48 | . | . | . |
| BWS-TT-ECA vs RA-GT | 0 | 0 | 0.07 | . | 0.07 | . | . | . |
| BWS-TT-ECA vs TT | 0 | 0 | 0.12 | . | 0.12 | . | . | . |
| BWS-TT-ECA vs VR-GT | 0 | 0 | -0.19 | . | -0.19 | . | . | . |
| DT-BGT vs CON | 0 | 0 | 1.64 | . | 1.64 | . | . | . |
| DT-BGT vs EC-BGT | 0 | 0 | 0.44 | . | 0.44 | . | . | . |
| DT-BGT vs RA-GT | 0 | 0 | 0.99 | . | 0.99 | . | . | . |
| DT-BGT vs TT | 1 | 1 | 1.04 | . |  | . | . | . |
| DT-BGT vs VR-GT | 0 | 0 | 0.73 | . | 0.73 | . | . | . |
| EC-BGT vs CON | 0 | 0 | 1.64 | . | 1.64 | . | . | . |
| EC-BGT vs RA-GT | 0 | 0 | 0.55 | . | 0.55 | . | . | . |
| EC-BGT vs TT | 1 | 0.82 | 0.6 | 0.78 | -0.24 | -0.33 | -0.72 | 0.2253 |
| EC-BGT vs VR-GT | 0 | 0 | 0.29 | . | 0.29 | . | . | . |
| RA-GT vs CON | 3 | 0.81 | 0.65 | 0.59 | 0.91 | 0.66 | 0.95 | 0.471 |
| RA-GT vs TT | 0 | 0 | 0.05 | . | 0.05 | . | . | . |
| RA-GT vs VR-GT | 1 | 0.59 | -0.26 | -0.27 | -0.24 | 1.02 | 1.21 | 0.966 |
| TT vs CON | 0 | 0 | 0.6 | . | 0.6 | . | . | . |
| TT vs VR-GT | 1 | 0.8 | -0.31 | -0.1 | -1.12 | -0.24 | -0.04 | 0.2253 |
| VR-GT vs CON | 1 | 0.32 | 0.91 | 1.36 | 0.7 | -1.12 | 1.21 | 0.3401 |

AQE-BGT, Aquatic balance and gait training; BGT, balance and gait training; BGT-ECA, balance and gait training with external cues; BWS-TT, body weight supported treadmill training ; BWS-TT-ECA, body weight supported treadmill training with external cues; CON, Control group; DT-BGT, dual-task gait training; EC-BGT, eyes closed gait training; RA-GT, robotic-assisted gait training; RA-GT-ECA, robotic-assisted gait training with external cues; TT, treadmill gait training; VR-GT, virtual reality gait training

**9.4 ‘Hot spots’ of inconsistency: Proactive balance**

| Random effects model | |
| --- | --- |
| k | - Number of studies providing direct evidence |
| prop | - Direct evidence proportion |
| nma | - Estimated treatment effect (SMD) in network meta- analysis |
| direct | - Estimated treatment effect (SMD) derived from direct evidence |
| indir. | - Estimated treatment effect (SMD) derived from indirect evidence |
| Diff | - Difference between direct and indirect treatment estimates |
| z | - z-value of test for disagreement (direct versus indirect) |
| P | - p-value of test for disagreement (direct versus indirect) |

| Comparison | k | prop | nma | direct | indir. | Diff | z | p-value |
| --- | --- | --- | --- | --- | --- | --- | --- | --- |
| BGT vs BGT-ECA | 4 | 0.68 | -0.03 | -0.31 | 0.56 | -0.88 | -1.69 | 0.0905 |
| BGT vs BWS-TT | 1 | 1 | 0.49 | 0.49 | . | . | . | . |
| BGT vs BWS-TT-ECA | 0 | 0 | -1.11 | . | -1.11 | . | . | . |
| BGT vs CON | 2 | 0.36 | 0.45 | 0.59 | 0.38 | 0.21 | 0.41 | 0.6801 |
| BGT vs DT-BGT | 0 | 0 | 0.67 | . | 0.67 | . | . | . |
| BGT vs EC-BGT | 0 | 0 | 0.17 | . | 0.17 | . | . | . |
| BGT vs RA-GT | 2 | 0.41 | 0.03 | 0.06 | 0.01 | 0.06 | 0.1 | 0.921 |
| BGT vs RA-GT-ECA | 0 | 0 | -0.08 | . | -0.08 | . | . | . |
| BGT vs TT | 0 | 0 | 0.95 | . | 0.95 | . | . | . |
| BGT vs TT-ECA | 0 | 0 | -0.58 | . | -0.58 | . | . | . |
| BGT vs VR-GT | 1 | 0.48 | -0.48 | 0.04 | -0.98 | 1.02 | 1.52 | 0.1274 |
| BGT-ECA vs BWS-TT | 0 | 0 | 0.53 | . | 0.53 | . | . | . |
| BGT-ECA vs BWS-TT-ECA | 0 | 0 | -1.08 | . | -1.08 | . | . | . |
| BGT-ECA vs CON | 2 | 0.51 | 0.48 | -0.00 | 0.99 | -0.99 | -1.91 | 0.0563 |
| BGT-ECA vs DT-BGT | 0 | 0 | 0.7 | . | 0.7 | . | . | . |
| BGT-ECA vs EC-BGT | 0 | 0 | 0.21 | . | 0.21 | . | . | . |
| BGT-ECA vs RA-GT | 0 | 0 | 0.06 | . | 0.06 | . | . | . |
| BGT-ECA vs RA-GT-ECA | 0 | 0 | -0.04 | . | -0.04 | . | . | . |
| BGT-ECA vs TT | 0 | 0 | 0.99 | . | 0.99 | . | . | . |
| BGT-ECA vs TT-ECA | 1 | 0.4 | -0.55 | -0.40 | -0.65 | 0.26 | 0.33 | 0.745 |
| BGT-ECA vs VR-GT | 0 | 0 | -0.45 | . | -0.45 | . | . | . |
| BWS-TT vs BWS-TT-ECA | 1 | 1 | -1.61 | -1.61 | . | . | . | . |
| BWS-TT vs CON | 0 | 0 | -0.04 | . | -0.04 | . | . | . |
| BWS-TT vs DT-BGT | 0 | 0 | 0.18 | . | 0.18 | . | . | . |
| BWS-TT vs EC-BGT | 0 | 0 | -0.32 | . | -0.32 | . | . | . |
| BWS-TT vs RA-GT | 0 | 0 | -0.46 | . | -0.46 | . | . | . |
| BWS-TT vs RA-GT-ECA | 0 | 0 | -0.57 | . | -0.57 | . | . | . |
| BWS-TT vs TT | 0 | 0 | 0.46 | . | 0.46 | . | . | . |
| BWS-TT vs TT-ECA | 0 | 0 | -1.08 | . | -1.08 | . | . | . |
| BWS-TT vs VR-GT | 0 | 0 | -0.98 | . | -0.98 | . | . | . |
| BWS-TT-ECA vs CON | 0 | 0 | 1.57 | . | 1.57 | . | . | . |
| BWS-TT-ECA vs DT-BGT | 0 | 0 | 1.78 | . | 1.78 | . | . | . |
| BWS-TT-ECA vs EC-BGT | 0 | 0 | 1.29 | . | 1.29 | . | . | . |
| BWS-TT-ECA vs RA-GT | 0 | 0 | 1.14 | . | 1.14 | . | . | . |
| BWS-TT-ECA vs RA-GT-ECA | 0 | 0 | 1.04 | . | 1.04 | . | . | . |
| BWS-TT-ECA vs TT | 0 | 0 | 2.07 | . | 2.07 | . | . | . |
| BWS-TT-ECA vs TT-ECA | 0 | 0 | 0.53 | . | 0.53 | . | . | . |
| BWS-TT-ECA vs VR-GT | 0 | 0 | 0.63 | . | 0.63 | . | . | . |
| DT-BGT vs CON | 0 | 0 | -0.22 | . | -0.22 | . | . | . |
| EC-BGT vs CON | 1 | 1 | 0.28 | 0.28 | . | . | . | . |
| RA-GT vs CON | 3 | 0.62 | 0.42 | 0.37 | 0.51 | -0.15 | -0.3 | 0.762 |
| RA-GT-ECA vs CON | 1 | 0.53 | 0.53 | 0.85 | 0.17 | 0.68 | 0.85 | 0.398 |
| TT vs CON | 1 | 0.3 | -0.50 | 0.23 | -0.82 | 1.05 | 1.47 | 0.1416 |
| TT-ECA vs CON | 1 | 0.36 | 1.04 | 1.17 | 0.96 | 0.21 | 0.27 | 0.7875 |
| VR-GT vs CON | 1 | 0.34 | 0.94 | 1.51 | 0.64 | 0.87 | 1.22 | 0.2215 |
| DT-BGT vs EC-BGT | 0 | 0 | -0.50 | . | -0.50 | . | . | . |
| DT-BGT vs RA-GT | 0 | 0 | -0.64 | . | -0.64 | . | . | . |
| DT-BGT vs RA-GT-ECA | 0 | 0 | -0.75 | . | -0.75 | . | . | . |
| DT-BGT vs TT | 1 | 1 | 0.28 | 0.28 | . | . | . | . |
| DT-BGT vs TT-ECA | 0 | 0 | -1.26 | . | -1.26 | . | . | . |
| DT-BGT vs VR-GT | 0 | 0 | -1.16 | . | -1.16 | . | . | . |
| EC-BGT vs RA-GT | 0 | 0 | -0.14 | . | -0.14 | . | . | . |
| EC-BGT vs RA-GT-ECA | 0 | 0 | -0.25 | . | -0.25 | . | . | . |
| EC-BGT vs TT | 0 | 0 | 0.78 | . | 0.78 | . | . | . |
| EC-BGT vs TT-ECA | 0 | 0 | -0.76 | . | -0.76 | . | . | . |
| EC-BGT vs VR-GT | 0 | 0 | -0.66 | . | -0.66 | . | . | . |
| RA-GT vs RA-GT-ECA | 1 | 0.46 | -0.11 | -0.03 | -0.17 | 0.14 | 0.18 | 0.8601 |
| RA-GT vs TT | 2 | 0.37 | 0.92 | 1.11 | 0.81 | 0.29 | 0.41 | 0.6796 |
| RA-GT vs TT-ECA | 0 | 0 | -0.62 | . | -0.62 | . | . | . |
| RA-GT vs VR-GT | 0 | 0 | -0.51 | . | -0.51 | . | . | . |
| RA-GT-ECA vs TT | 0 | 0 | 1.03 | . | 1.03 | . | . | . |
| RA-GT-ECA vs TT-ECA | 0 | 0 | -0.51 | . | -0.51 | . | . | . |
| RA-GT-ECA vs VR-GT | 1 | 0.6 | -0.41 | -0.66 | -0.03 | -0.63 | -0.69 | 0.4875 |
| TT vs TT-ECA | 3 | 0.83 | -1.54 | -1.50 | 1.74 | 0.25 | 0.3 | 0.7676 |
| TT vs VR-GT | 2 | 0.53 | -1.44 | -1.59 | -1.26 | -0.33 | -0.47 | 0.6399 |
| TT-ECA vs VR-GT | 0 | 0 | 0.1 | . | 0.1 | . | . | . |

BGT, balance and gait training; BGT-ECA, balance and gait training with external cues; BWS-TT, body weight supported treadmill training; BWS-TT-ECA, body weight supported treadmill training with external cues; CON, Control group; DT-BGT, dual-task gait training; EC-BGT, eyes closed gait training; RA-GT, robotic-assisted gait training; RA-GT-ECA, robotic-assisted gait training with external cues; TT, treadmill gait training; TT-ECA, treadmill gait training with external cues; VR-GT, virtual reality gait training

**Appendix 10: Grading the evidence of the network meta-analysis using GRADE**

**10.1 Details of GRADE assessment for all pairwise comparisons within the balance test batteries network**

| **Comparison** | **k** | **prop** | **Direct SMD [95% CI]** | **Quality of evidence** | **Indirect SMD [95% CI]** | **Quality of evidence** | **NMA SMD [95% CI]** | **Quality of evidence** | **P-value** |
| --- | --- | --- | --- | --- | --- | --- | --- | --- | --- |
| BGT vs BGT-ECA | 3 | 0.59 | -0.46(-1.29, 0.37) | Very low*‡ | -0.20(-1.21, 0.80) | Very low*‡ | -0.36(-1.00, 0.29) | Very low*‡ | 0.7040 |
| BGT vs BWS-TT | 4 | 0.57 | -0.13(-0.75, 0.49) | Very low*‡ | -0.20(-0.91, 0.51) | Very low*‡ | -0.16(-0.63, 0.31) | Very low*‡ | 0.8876 |
| BGT vs BWS-TT-ECA | 1 | 0.34 | -0.50(-1.78, 0.78) | Very low*‡ | -1.11(-2.04, -0.19) | Very low*‡ | -0.90(-1.66, -0.15) | Very low*‡ | 0.4466 |
| BGT vs CON | 1 | 0.11 | 1.32(-0.06, 2.70) | Very low*‡ | -0.05(-0.53, 0.43) | Very low*‡ | 0.10(-0.35, 0.55) | Very low*‡ | 0.0655 |
| BGT vs EC-BGT | 1 | 0.32 | -0.74(-2.21, 0.73) | Very low*‡ | -0.59(-1.61, 0.42) | Very low*‡ | -0.64(-1.48, 0.19) | Very low*‡ | 0.8728 |
| BGT vs RA-GT | 6 | 0.62 | -0.54(-1.08, 0.01) | Very low*‡ | 0.04(-0.66, 0.73) | Very low*‡ | -0.32(-0.75, 0.11) | Very low*‡ | 0.2037 |
| BGT-ECA vs CON | 2 | 0.54 | 0.34(-0.54, 1.22) | Very low*‡ | 0.59(-0.37, 1.56) | Very low*‡ | 0.46(-0.20, 1.11) | Very low*‡ | 0.7040 |
| BWS-TT vs BWS-TT-ECA | 3 | 0.86 | -0.79(-1.52, -0.05) | Very low*‡ | -0.46(-2.32, 1.39) | Very low*‡ | -0.74(-1.43, -0.06) | Very low*‡ | 0.7495 |
| BWS-TT vs CON | 4 | 0.53 | 0.24(-0.40, 0.88) | Very low*‡ | 0.29(-0.39, 0.97) | Very low*‡ | 0.26(-0.21, 0.73) | Very low*‡ | 0.9200 |
| BWS-TT vs DT-BGT | 1 | 0.49 | 0.06(-1.23, 1.35) | Very low*‡ | -0.00(-1.26, 1.25) | Very low*‡ | 0.03(-0.87, 0.93) | Very low*‡ | 0.9463 |
| BWS-TT vs EC-BGT | 1 | 0.38 | -0.49(-1.86, 0.88) | Very low*‡ | -0.47(-1.55, 0.61) | Very low*‡ | -0.48(-1.33, 0.37) | Very low*‡ | 0.9841 |
| BWS-TT vs RA-GT | 1 | 0.12 | 0.36(-1.09, 1.81) | Low‡ | -0.23(-0.78, 0.31) | Very low*‡ | -0.16(-0.67, 0.35) | Low‡ | 0.4541 |
| BWS-TT-ECA vs CON | 1 | 0.37 | 1.05(-0.18, 2.29) | Low‡ | 0.97(0.04, 1.91) | Very low*‡ | 1.00(0.26, 1.75) | Low‡ | 0.9190 |
| DT-BGT vs TT | 2 | 0.74 | 0.66(-0.26, 1.58) | Low‡ | 0.60(-0.95, 2.14) | Very low*‡ | 0.64(-0.15, 1.43) | Low‡ | 0.9463 |
| EC-BGT vs CON | 2 | 0.65 | 0.70(-0.30, 1.70) | Very low*‡ | 0.81(-0.56, 2.18) | Very low*‡ | 0.74(-0.07, 1.55) | Very low*‡ | 0.8991 |
| RA-GT vs CON | 7 | 0.64 | 0.21(-0.28, 0.69) | Very low*‡ | 0.79(0.15, 1.43) | Very low*‡ | 0.42(0.03, 0.80) | Very low*‡ | 0.1553 |
| RA-GT vs RA-GT-ECA | 1 | 0.45 | -0.16(-1.54, 1.22) | Low‡ | -0.42(-1.68, 0.83) | Very low*‡ | -0.30(-1.23, 0.62) | Low‡ | 0.7788 |
| RA-GT vs TT | 2 | 0.34 | 1.31(0.12, 2.51) | Low*† | 0.57(-0.29, 1.44) | Very low*‡ | 0.83(0.13, 1.53) | Low*† | 0.3258 |
| RA-GT-ECA vs CON | 1 | 0.49 | 0.98(-0.34, 2.30) | Very low*‡ | 0.47(-0.82, 1.77) | Very low*‡ | 0.72(-0.20, 1.65) | Very low*‡ | 0.5867 |
| RA-GT-ECA vs VR-GT | 1 | 0.59 | -0.71(-2.05, 0.63) | Very low*‡ | -0.54(-2.15, 1.06) | Very low*‡ | -0.64(-1.67, 0.38) | Very low*‡ | 0.8733 |
| TT vs CON | 1 | 0.3 | 0.28(-0.97, 1.53) | Low‡ | -0.70(-1.51, 0.11) | Very low*‡ | -0.41(-1.09, 0.27) | Low‡ | 0.1955 |
| TT vs TT-ECA | 2 | 1 | -0.74(-1.66, 0.18) | Low‡ |  |  | -0.74(-1.66, 0.18) | Low‡ |  |
| TT vs VR-GT | 3 | 0.65 | -1.85(-2.75, -0.94) | Low*† | -1.65(-2.87, -0.42) | Low‡ | -1.78(-2.50, -1.05) | Low‡ | 0.7987 |
| VR-GT vs CON | 2 | 0.58 | 1.32(0.34, 2.30) | Low*† | 1.43(0.28, 2.59) | Low‡ | 1.37(0.62, 2.11) | Low‡ | 0.8866 |
| BGT vs DT-BGT | 0 | 0 |  |  | -0.13(-1.08, 0.81) | Very low*‡ | -0.13(-1.08, 0.81) | Very low*‡ |  |
| BGT vs RA-GT-ECA | 0 | 0 |  |  | -0.62(-1.61, 0.36) | Very low*‡ | -0.62(-1.61, 0.36) | Very low*‡ |  |
| BGT vs TT | 0 | 0 |  |  | 0.51(-0.25, 1.27) | Very low*‡ | 0.51(-0.25, 1.27) | Very low*‡ |  |
| BGT vs TT-ECA | 0 | 0 |  |  | -0.23(-1.42, 0.96) | Very low*‡ | -0.23(-1.42, 0.96) | Very low*‡ |  |
| BGT vs VR-GT | 0 | 0 |  |  | -1.27(-2.11, -0.43) | Very low*‡ | -1.27(-2.11, -0.43) | Very low*‡ |  |
| BGT-ECA vs BWS-TT | 0 | 0 |  |  | 0.20(-0.54, 0.93) | Very low*‡ | 0.20(-0.54, 0.93) | Very low*‡ |  |
| BGT-ECA vs BWS-TT-ECA | 0 | 0 |  |  | -0.55(-1.48, 0.39) | Very low*‡ | -0.55(-1.48, 0.39) | Very low*‡ |  |
| BGT-ECA vs DT-BGT | 0 | 0 |  |  | 0.22(-0.86, 1.30) | Very low*‡ | 0.22(-0.86, 1.30) | Very low*‡ |  |
| BGT-ECA vs EC-BGT | 0 | 0 |  |  | -0.29(-1.28, 0.71) | Very low*‡ | -0.29(-1.28, 0.71) | Very low*‡ |  |
| BGT-ECA vs RA-GT | 0 | 0 |  |  | 0.04(-0.66, 0.73) | Very low*‡ | 0.04(-0.66, 0.73) | Very low*‡ |  |
| BGT-ECA vs RA-GT-ECA | 0 | 0 |  |  | -0.27(-1.38, 0.84) | Very low*‡ | -0.27(-1.38, 0.84) | Very low*‡ |  |
| BGT-ECA vs TT | 0 | 0 |  |  | 0.86(-0.05, 1.78) | Very low*‡ | 0.86(-0.05, 1.78) | Very low*‡ |  |
| BGT-ECA vs TT-ECA | 0 | 0 |  |  | 0.13(-1.17, 1.42) | Very low*‡ | 0.13(-1.17, 1.42) | Very low*‡ |  |
| BGT-ECA vs VR-GT | 0 | 0 |  |  | -0.91(-1.89, 0.06) | Very low*‡ | -0.91(-1.89, 0.06) | Very low*‡ |  |
| BWS-TT vs RA-GT-ECA | 0 | 0 |  |  | -0.46(-1.47, 0.54) | Low‡ | -0.46(-1.47, 0.54) | Low‡ |  |
| BWS-TT vs TT | 0 | 0 |  |  | 0.67(-0.08, 1.42) | Very low*‡ | 0.67(-0.08, 1.42) | Very low*‡ |  |
| BWS-TT vs TT-ECA | 0 | 0 |  |  | -0.07(-1.26, 1.12)) | Low‡ | -0.07(-1.26, 1.12)) | Low‡ |  |
| BWS-TT vs VR-GT | 0 | 0 |  |  | -1.11(-1.95, 0.26) | Very low*‡ | -1.11(-1.95, 0.26) | Very low*‡ |  |
| BWS-TT-ECA vs DT-BGT | 0 | 0 |  |  | 0.77(-0.33, 1.87) | Very low*‡ | 0.77(-0.33, 1.87) | Very low*‡ |  |
| BWS-TT-ECA vs EC-BGT | 0 | 0 |  |  | 0.26(-0.78, 1.31) | Very low*‡ | 0.26(-0.78, 1.31) | Very low*‡ |  |
| BWS-TT-ECA vs RA-GT | 0 | 0 |  |  | 0.58(-0.20, 1.37) | Very low*‡ | 0.58(-0.20, 1.37) | Very low*‡ |  |
| BWS-TT-ECA vs RA-GT-ECA | 0 | 0 |  |  | 0.28(-0.88, 1.45) | Very low*‡ | 0.28(-0.88, 1.45) | Very low*‡ |  |
| BWS-TT-ECA vs TT | 0 | 0 |  |  | 1.41(0.45, 2.38) | Low‡ | 1.41(0.45, 2.38) | Low‡ |  |
| BWS-TT-ECA vs TT-ECA | 0 | 0 |  |  | 0.67(-0.66, 2.01) | Low‡ | 0.67(-0.66, 2.01) | Low‡ |  |
| BWS-TT-ECA vs VR-GT | 0 | 0 |  |  | -0.36(-1.40, 0.67) | Low‡ | -0.36(-1.40, 0.67) | Low‡ |  |
| DT-BGT vs CON | 0 | 0 |  |  | 0.23(-0.67, 1.13) | Very low*‡ | 0.23(-0.67, 1.13) | Very low*‡ |  |
| DT-BGT vs EC-BGT | 0 | 0 |  |  | -0.51(-1.68, 0.67) | Very low*‡ | -0.51(-1.68, 0.67) | Very low*‡ |  |
| DT-BGT vs RA-GT | 0 | 0 |  |  | -0.19(-1.11, 0.73) | Very low*‡ | -0.19(-1.11, 0.73) | Very low*‡ |  |
| DT-BGT vs RA-GT-ECA | 0 | 0 |  |  | -0.49(-1.72, 0.74) | Very low*‡ | -0.49(-1.72, 0.74) | Very low*‡ |  |
| DT-BGT vs TT-ECA | 0 | 0 |  |  | -0.10(-1.31, 1.12) | Low‡ | -0.10(-1.31, 1.12) | Low‡ |  |
| DT-BGT vs VR-GT | 0 | 0 |  |  | -1.13(-2.15, -0.12) | Low‡ | -1.13(-2.15, -0.12) | Low‡ |  |
| EC-BGT vs RA-GT | 0 | 0 |  |  | 0.32(-0.53, 1.18) | Very low*‡ | 0.32(-0.53, 1.18) | Very low*‡ |  |
| EC-BGT vs RA-GT-ECA | 0 | 0 |  |  | 0.02(-1.19, 1.23) | Very low*‡ | 0.02(-1.19, 1.23) | Very low*‡ |  |
| EC-BGT vs TT | 0 | 0 |  |  | 1.15(0.12, 2.18) | Very low*‡ | 1.15(0.12, 2.18) | Very low*‡ |  |
| EC-BGT vs TT-ECA | 0 | 0 |  |  | 0.41(-0.97, 1.79) | Very low*‡ | 0.41(-0.97, 1.79) | Very low*‡ |  |
| EC-BGT vs VR-GT | 0 | 0 |  |  | -0.63(-1.71, 0.46) | Very low*‡ | -0.63(-1.71, 0.46) | Very low*‡ |  |
| RA-GT vs TT-ECA | 0 | 0 |  |  | 0.09(-1.07, 1.25) | Very low*‡ | 0.09(-1.07, 1.25) | Very low*‡ |  |
| RA-GT vs VR-GT | 0 | 0 |  |  | -0.95(-1.74, -0.16) | Very low*‡ | -0.95(-1.74, -0.16) | Very low*‡ |  |
| RA-GT-ECA vs TT | 0 | 0 |  |  | 1.13(0.07, 2.19) | Very low*‡ | 1.13(0.07, 2.19) | Very low*‡ |  |
| RA-GT-ECA vs TT-ECA | 0 | 0 |  |  | 0.39(-1.01, 1.80) | Very low*‡ | 0.39(-1.01, 1.80) | Very low*‡ |  |
| TT-ECA vs CON | 0 | 0 |  |  | 0.33(-0.81, 1.47) | Low‡ | 0.33(-0.81, 1.47) | Low‡ |  |
| TT-ECA vs VR-GT | 0 | 0 |  |  | -1.04(-2.21, 0.14) | Low‡ | -1.04(-2.21, 0.14) | Low‡ |  |

k = Number of studies providing direct evidence, prop = proportion of direct evidence, NMA (SMD) = estimated treatment effect (SMD) in network meta-analysis, direct (SMD) = estimated treatment effect (SMD) derived from direct evidence, indirect (SMD) = estimated treatment effect (SMD) derived from indirect evidence, p = p-value of test for disagreement (difference between direct and indirect evidence)

Reasons for downgrading: †Imprecision (1 downgrade), ‡Severe imprecision (2 downgrades), *risk of bias (1 downgrade)

The p-value indicated that the findings between direct and indirect comparisons was not statistically significant and therefore no comparisons have been downgraded based on inconsistency

**10.2 Details of GRADE assessment for all pairwise comparisons within the dynamic steady-state balance network**

| **Comparison** | **k** | **prop** | **Direct SMD [95% CI]** | **Quality of evidence** | **Indirect SMD [95% CI]** | **Quality of evidence** | **NMA SMD [95% CI]** | **Quality of evidence** | **P-value** |
| --- | --- | --- | --- | --- | --- | --- | --- | --- | --- |
| AQE-BGT vs BGT | 1 | 0.53 | -0.06(-1.16, 1.04) | Low‡ | -0.28(-1.44, 0.88) | Low‡ | 0.41(-0.25, 1.07) | Low‡ | 0.7854 |
| AQE-BGT vs CON | 1 | 0.51 | 0.16(-0.96, 1.28) | Low‡ | 0.38(-0.76, 1.53) | Low†§ | -0.13(-0.44, 0.18) | Low†§ | 0.7854 |
| BGT vs BGT-ECA | 7 | 0.68 | -0.15(-0.57, 0.27) | Low‡ | -0.02(-0.64, 0.59) | Low†§ | -0.43(-0.89, 0.03) | Low‡ | 0.7373 |
| BGT vs BWS-TT | 5 | 0.58 | -0.61(-1.04, -0.18) | Low†§ | 0.10(-0.41, 0.61) | Low†§ | -0.22(-0.99, 0.55) | Low†§ | 0.0365 |
| BGT vs BWS-TT-ECA | 2 | 0.49 | -0.68(-1.38, 0.02) | Very low*‡ | -0.80(-1.5, -0.11) | Low‡ | -0.23(-0.80, 0.34) | Very low*‡ | 0.8073 |
| BGT vs CON | 2 | 0.13 | 1.37(0.55, 2.20) | Low†§ | 0.29(-0.03, 0.61) | Very low*‡ | 0.54(0.17, 0.92) | Low†§ | 0.0166 |
| BGT vs EC-BGT | 1 | 0.18 | -1.25(-2.56, 0.06) | Very low*‡ | -0.38(-0.99, 0.23) | Low†§ | -0.74(-1.23, -0.25) | Low†§ | 0.2401 |
| BGT vs RA-GT | 5 | 0.49 | -0.11(-0.55, 0.34) | Very low*‡ | -0.16(-0.60, 0.28) | Very low*‡ | 0.74(0.41, 1.08) | Very low*‡ | 0.8671 |
| BGT vs VR-GT | 1 | 0.28 | -0.02(-0.9, 0.86) | Low‡ | -0.48(-1.03, 0.07) | Very low*‡ | -0.98(-1.46, -0.49) | Low‡ | 0.3861 |
| BGT-ECA vs CON | 3 | 0.44 | 0.59(0.02, 1.16) | Moderate† | 0.51(0.01, 1.01) | Very low*‡ | -0.79(-1.26, -0.31) | Moderate† | 0.8413 |
| BGT-ECA vs TT-ECA | 1 | 0.27 | -0.95(-2.14, 0.24) | Very low*‡ | -0.25(-0.96, 0.47) | Very low*‡ | -0.27(-1.13, 0.58) | Very low*‡ | 0.3213 |
| BWS-TT vs BWS-TT-ECA | 4 | 0.85 | -0.43(-0.93, 0.07) | Low‡ | -0.42(-1.62, 0.79) | Very low*‡ | -0.29(-1.37, 0.78) | Low‡ | 0.9773 |
| BWS-TT vs CON | 5 | 0.5 | 0.70(0.23, 1.18) | Moderate† | 0.78(0.31, 1.26) | Very low*‡ | -0.06(-1.08, 0.95) | Moderate†† | 0.8192 |
| BWS-TT vs DT-BGT | 1 | 0.43 | -0.01(-1.02, 0.99) | Low‡ | 0.73(-0.14, 1.59) | Very low*‡ | -0.70(-1.65, 0.24) | Low‡ | 0.2746 |
| BWS-TT vs EC-BGT | 1 | 0.26 | -0.58(-1.69, 0.54) | Very low*‡ | -0.10(-0.77, 0.56) | Very low*‡ | -0.30(-1.15, 0.53) | Very low*‡ | 0.4743 |
| BWS-TT vs RA-GT | 1 | 0.1 | -0.21(-1.41, 1.00) | Very low*‡ | 0.22(-0.18, 0.61) | Very low*‡ | -0.29(-1.37, 0.78) | Very low*‡ | 0.5119 |
| BWS-TT-ECA vs CON | 1 | 0.29 | 0.83(-0.11, 1.76) | Low‡ | 1.32(0.72, 1.93) | Very low*‡ | -0.13(-0.89, 0.63) | Low‡ | 0.383 |
| DT-BGT vs TT | 2 | 0.71 | 0.12(-0.59, 0.83) | Low‡ | 0.86(-0.26, 1.98) | Low‡ | 0.74(0.28, 1.20) | Low‡ | 0.2746 |
| EC-BGT vs CON | 2 | 0.4 | 0.94(0.10, 1.78) | Moderate† | 0.99(0.31, 1.67) | Very low*‡ | 0.84(0.07, 1.62) | Moderate† | 0.9233 |
| EC-BGT vs TT | 2 | 0.55 | 0.77(0.07, 1.47) | Moderate† | 1.22(0.44, 2.00) | Low‡ | 0.61(-0.26, 1.48) | Moderate† | 0.4026 |
| RA-GT vs CON | 8 | 0.62 | 0.37(0.02, 0.72) | Moderate† | 0.89(0.45, 1.33) | Very low*‡ | 0.39(-0.25, 1.03) | Moderate† | 0.0713 |
| RA-GT vs RA-GT-ECA | 1 | 0.42 | -0.07(-1.19, 1.05) | Low‡ | 0.06(-0.90, 1.02) | Very low*‡ | 0.33(-0.32, 0.99) | Low‡ | 0.8575 |
| RA-GT vs TT | 3 | 0.26 | 1.23(0.41, 2.04) | Moderate† | 0.33(-0.15, 0.82) | Low‡ | 0.64(-0.12, 1.40) | Moderate† | 0.0644 |
| RA-GT vs VR-GT | 1 | 0.19 | 0.41(-0.63, 1.46) | Very low*‡ | -0.37(-0.89, 0.14) | Very low*‡ | -0.23(-1.19, 1.19) | Very low*‡ | 0.1873 |
| RA-GT-ECA vs CON | 1 | 0.5 | 0.70(-0.34, 1.74) | Very low*‡ | 0.43(-0.60, 1.46) | Very low*‡ | -0.65(-1.40, 0.11) | Very low*‡ | 0.7149 |
| RA-GT-ECA vs VR-GT | 1 | 0.52 | -0.49(-1.55, 0.58) | Very low*‡ | 0.06(-1.05, 1.18) | Very low*‡ | 0.41(-0.47, 1.29) | Very low*‡ | 0.4851 |
| TT vs CON | 2 | 0.29 | 0.14(-0.59, 0.86) | Low‡ | -0.06(-0.52, 0.41) | Very low*‡ | -0.01(-0.69, 0.68) | Low‡ | 0.6614 |
| TT vs TT-ECA | 4 | 0.84 | -0.87(-1.40, -0.34) | Moderate† | -1.55(-2.74, -0.35) | Very low*‡ | 0.19(-0.45, 0.82) | Moderate† | 0.3095 |
| TT vs VR-GT | 3 | 0.49 | -1.02(-1.69, -0.35) | Moderate† | -0.56(-1.22, 0.10) | Low‡ | -0.41(-1.00, 0.17) | Moderate† | 0.3361 |
| TT-ECA vs CON | 1 | 0.23 | 1.12(-0.04, 2.28) | Very low*‡ | 0.94(0.29, 1.58) | Low‡ | 0.56(-0.23, 1.35) | Very low*‡ |  |
| VR-GT vs CON | 2 | 0.33 | 1.06(0.28, 1.85) | Low*† | 0.65(0.11, 1.20) | Low‡ | 0.19(-0.45, 0.83) | Low‡ | 0.3991 |
| AQE-BGT vs BGT-ECA | 0 | 0 |  |  | -0.35(-0.82, 0.12) | Low‡ | -0.35(-0.82, 0.12) | Low‡ |  |
| AQE-BGT vs BWS-TT | 0 | 0 |  |  | -0.17(-0.97, 0.63) | Low‡ | -0.17(-0.97, 0.63) | Low‡ |  |
| AQE-BGT vs BWS-TT-ECA | 0 | 0 |  |  | 0.00(-0.72, 0.73) | Low‡ | 0.00(-0.72, 0.73) | Low‡ |  |
| AQE-BGT vs DT-BGT | 0 | 0 |  |  | 0.33(-0.27, 0.93) | Low‡ | 0.33(-0.27, 0.93) | Low‡ |  |
| AQE-BGT vs EC-BGT | 0 | 0 |  |  | 0.00(-0.39, 0.40) | Low‡ | 0.00(-0.39, 0.40) | Low‡ |  |
| AQE-BGT vs RA-GT | 0 | 0 |  |  | -0.11(-0.45, 0.24) | Low‡ | -0.11(-0.45, 0.24) | Low‡ |  |
| AQE-BGT vs RA-GT-ECA | 0 | 0 |  |  | -0.15(-0.57, 0.27) | Very low*‡ | -0.15(-0.57, 0.27) | Very low*‡ |  |
| AQE-BGT vs TT | 0 | 0 |  |  | 0.18(-0.20, 0.55) | Low‡ | 0.18(-0.20, 0.55) | Low‡ |  |
| AQE-BGT vs TT-ECA | 0 | 0 |  |  | 0.57(0.29, 0.84) | Very low*‡ | 0.57(0.29, 0.84) | Very low*‡ |  |
| AQE-BGT vs VR-GT | 0 | 0 |  |  | -0.22(-0.68, 0.24) | Low‡ | -0.22(-0.68, 0.24) | Low‡ |  |
| BGT vs DT-BGT | 0 | 0 |  |  | 0.10(-0.57, 0.77) | Low‡ | 0.10(-0.57, 0.77) | Low‡ |  |
| BGT vs RA-GT-ECA | 0 | 0 |  |  | 0.56(-0.17, 1.29) | Very low*‡ | 0.56(-0.17, 1.29) | Very low*‡ |  |
| BGT vs TT | 0 | 0 |  |  | 0.97(0.45, 1.49) | Very low*‡ | 0.97(0.45, 1.49) | Very low*‡ |  |
| BGT vs TT-ECA | 0 | 0 |  |  | 1.18(0.67, 1.68) | Very low*‡ | 1.18(0.67, 1.68) | Very low*‡ |  |
| BGT-ECA vs BWS-TT | 0 | 0 |  |  | 0.97(0.44, 1.50) | Low‡ | 0.97(0.44, 1.50) | Low‡ |  |
| BGT-ECA vs BWS-TT-ECA | 0 | 0 |  |  | -0.44(-1.05, 0.18) | Very low*‡ | -0.44(-1.05, 0.18) | Very low*‡ |  |
| BGT-ECA vs DT-BGT | 0 | 0 |  |  | 0.79(0.34, 1.23) | Very low*‡ | 0.79(0.34, 1.23) | Very low*‡ |  |
| BGT-ECA vs EC-BGT | 0 | 0 |  |  | 0.98(0.42, 1.54) | Very low*‡ | 0.98(0.42, 1.54) | Very low*‡ |  |
| BGT-ECA vs RA-GT | 0 | 0 |  |  | 0.57(0.15, 0.98) | Very low*‡ | 0.57(0.15, 0.98) | Very low*‡ |  |
| BGT-ECA vs RA-GT-ECA | 0 | 0 |  |  | -0.54(-1.09, 0.01) | Very low*‡ | -0.54(-1.09, 0.01) | Very low*‡ |  |
| BGT-ECA vs TT | 0 | 0 |  |  | 0.43(0.13, 0.73) | Very low*‡ | 0.43(0.13, 0.73) | Very low*‡ |  |
| BGT-ECA vs VR-GT | 0 | 0 |  |  | -0.48(-1.32, 0.36) | Low‡ | -0.48(-1.32, 0.36) | Low‡ |  |
| BWS-TT vs RA-GT-ECA | 0 | 0 |  |  | 0.27(-0.61, 1.14) | Very low*‡ | 0.27(-0.61, 1.14) | Very low*‡ |  |
| BWS-TT vs TT | 0 | 0 |  |  | -0.71(-1.67, 0.25) | Very low*‡ | -0.71(-1.67, 0.25) | Very low*‡ |  |
| BWS-TT vs TT-ECA | 0 | 0 |  |  | -0.52(-1.42, 0.38) | Very low*‡ | -0.52(-1.42, 0.38) | Very low*‡ |  |
| BWS-TT vs VR-GT | 0 | 0 |  |  | 0.10(-0.57, 0.77) | Very low*‡ | 0.10(-0.57, 0.77) | Very low*‡ |  |
| BWS-TT-ECA vs DT-BGT | 0 | 0 |  |  | 0.43(0.00, 0.87) | Low‡ | 0.43(0.00, 0.87) | Low‡ |  |
| BWS-TT-ECA vs EC-BGT | 0 | 0 |  |  | -0.54(-1.13, 0.04) | Very low*‡ | -0.54(-1.13, 0.04) | Very low*‡ |  |
| BWS-TT-ECA vs RA-GT | 0 | 0 |  |  | -0.20(-0.64, 0.24) | Very low*‡ | -0.20(-0.64, 0.24) | Very low*‡ |  |
| BWS-TT-ECA vs RA-GT-ECA | 0 | 0 |  |  | -0.63(-1.21, -0.06) | Very low*‡ | -0.63(-1.21, -0.06) | Very low*‡ |  |
| BWS-TT-ECA vs TT | 0 | 0 |  |  | 0.21(-0.51, 0.93) | Very low*‡ | 0.21(-0.51, 0.93) | Very low*‡ |  |
| BWS-TT-ECA vs TT-ECA | 0 | 0 |  |  | 0.43(-0.18, 1.04) | Very low*‡ | 0.43(-0.18, 1.04) | Very low*‡ |  |
| BWS-TT-ECA vs VR-GT | 0 | 0 |  |  | -0.03(-0.44, 0.39) | Very low*‡ | -0.03(-0.44, 0.39) | Very low*‡ |  |
| DT-BGT vs CON | 0 | 0 |  |  | -0.02(-0.82, 0.78) | Low‡ | -0.02(-0.82, 0.78) | Low‡ |  |
| DT-BGT vs EC-BGT | 0 | 0 |  |  | 0.54(0.05, 1.04) | Low‡ | 0.54(0.05, 1.04) | Low‡ |  |
| DT-BGT vs RA-GT | 0 | 0 |  |  | -0.24(-0.78, 0.30) | Low‡ | -0.24(-0.78, 0.30) | Low‡ |  |
| DT-BGT vs RA-GT-ECA | 0 | 0 |  |  | 0.18(-0.60, 0.96) | Very low*‡ | 0.18(-0.60, 0.96) | Very low*‡ |  |
| DT-BGT vs TT-ECA | 0 | 0 |  |  | -0.23(-0.85, 0.38) | Very low*‡ | -0.23(-0.85, 0.38) | Very low*‡ |  |
| DT-BGT vs VR-GT | 0 | 0 |  |  | -0.04(-0.56, 0.47) | Low‡ | -0.04(-0.56, 0.47) | Low‡ |  |
| EC-BGT vs RA-GT | 0 | 0 |  |  | 0.20(-0.49, 0.90) | Very low*‡ | 0.20(-0.49, 0.90) | Very low*‡ |  |
| EC-BGT vs RA-GT-ECA | 0 | 0 |  |  | 0.61(0.07, 1.14) | Very low*‡ | 0.61(0.07, 1.14) | Very low*‡ |  |
| EC-BGT vs TT-ECA | 0 | 0 |  |  | 1.17(0.57, 1.78) | Very low*‡ | 1.17(0.57, 1.78) | Very low*‡ |  |
| EC-BGT vs VR-GT | 0 | 0 |  |  | 0.20(-0.53, 0.92) | Low*† | 0.20(-0.53, 0.92) | Low*† |  |
| RA-GT vs TT-ECA | 0 | 0 |  |  | -0.24(-0.91, 0.44) | Very low*‡ | -0.24(-0.91, 0.44) | Very low*‡ |  |
| RA-GT-ECA vs TT | 0 | 0 |  |  | -0.45(-1.18, 0.27) | Low‡ | -0.45(-1.18, 0.27) | Low‡ |  |
| RA-GT-ECA vs TT-ECA | 0 | 0 |  |  | 0.40(-0.15, 0.96) | Very low*‡ | 0.40(-0.15, 0.96) | Very low*‡ |  |
| TT-ECA vs VR-GT | 0 | 0 |  |  | -0.42(-1.31, 0.48) | Very low*‡ | -0.42(-1.31, 0.48) | Very low*‡ |  |

k = Number of studies providing direct evidence, prop = proportion of direct evidence, NMA (SMD) = estimated treatment effect (SMD) in network meta-analysis, direct (SMD) = estimated treatment effect (SMD) derived from direct evidence, indirect (SMD) = estimated treatment effect (SMD) derived from indirect evidence, p = p-value of test for disagreement (difference between direct and indirect evidence)

Reasons for downgrading: †Imprecision (1 downgrade), ‡Severe imprecision (2 downgrades), *risk of bias (1 downgrade), §inconsistency (1 downgrade)

**10.3 Details of GRADE assessment for all pairwise comparisons within the static steady-state balance network**

| **Comparison** | **k** | **prop** | **Direct SMD [95% CI]** | **Quality of evidence** | **Indirect SMD [95% CI]** | **Quality of evidence** | **NMA SMD [95% CI]** | **Quality of evidence** | **P-value** |
| --- | --- | --- | --- | --- | --- | --- | --- | --- | --- |
| AQE-BGT vs BGT | 1 | 0.66 | 0.64(-0.27, 1.55) | Low‡ | 2.01(0.73, 3.28) | Low‡ | 1.10(0.36, 1.84) | Low‡ | 0.0882 |
| AQE-BGT vs CON | 1 | 0.42 | 2.27(1.08, 3.46) | Low*† | 0.90(-0.11, 1.92) | Low‡ | 1.48(0.71, 2.25) | Low‡ | 0.0882 |
| BGT vs BGT-ECA | 4 | 1 | -0.51(-0.96, -0.06) | Very low*‡ |  |  | -0.51(-0.96, -0.06) | Very low*‡ |  |
| BGT vs BWS-TT | 1 | 0.48 | 0.16(-0.51, 0.83) | Low‡ | 0.30(-0.35, 0.95) | Very low*‡ | 0.26(-0.23, 0.70) | Low‡ | 0.7701 |
| BGT vs BWS-TT-ECA | 1 | 0.43 | -0.15(-0.89, 0.60) | Very low*‡ | -0.48(-1.12, 0.17) | Low‡ | -0.34(-0.82, 0.15) | Low‡ | 0.5089 |
| BGT vs CON | 1 | 0.28 | -0.08(-0.91, 0.74) | Low‡ | 0.57(0.04, 1.09) | Very low*‡ | 0.38(-0.06, 0.82) | Low‡ | 0.1935 |
| BGT vs EC-BGT | 1 | 0.62 | -0.43(-1.45, 0.59) | Very low*‡ | -1.45(-2.76, -0.15) | Very low*‡ | -0.82(-1.62, -0.01) | Very low*‡ | 0.2253 |
| BGT vs RA-GT | 1 | 0.34 | -0.58(-1.43, 0.27) | Very low*‡ | -0.11(-0.72, 0.50) | Low‡ | -0.27(-0.76, 0.22) | Low‡ | 0.3766 |
| BWS-TT vs BWS-TT-ECA | 2 | 0.8 | -0.64(-1.14, -0.14) | Moderate† | -0.31(-1.32, 0.69) | Very low*‡ | -0.57(-1.02, -0.12) | Moderate† | 0.5702 |
| BWS-TT vs CON | 1 | 0.59 | -0.03(-0.67, 0.61) | Low‡ | 0.40(-0.37, 1.17) | Low‡ | 0.15(-0.34, 0.64) | Low‡ | 0.3973 |
| BWS-TT-ECA vs CON | 1 | 0.58 | 1.05(0.38, 1.72) | Moderate† | 0.26(-0.53, 1.04) | Very low*‡ | 0.72(0.21, 1.23) | Moderate† | 0.1299 |
| DT-BGT vs TT | 1 | 1 | 1.04(0.24, 1.84) | Very low*‡ |  |  | 1.04(0.24, 1.84) | Very low*‡ |  |
| EC-BGT vs TT | 1 | 0.82 | 0.78(0.08, 1.49) | Moderate† | -0.24(-1.74, 1.26) | Very low*‡ | 0.60(-0.04, 1.24) | Moderate† | 0.2253 |
| RA-GT vs CON | 3 | 0.81 | 0.59(0.20, 0.98) | Moderate† | 0.91(1.12, 1.71) | Very low*‡ | 0.65(0.3, 1.00) | Moderate† | 0.471 |
| RA-GT vs VR-GT | 1 | 0.59 | -0.27(-1.07, 0.53) | Very low*‡ | -0.24(-1.2, 0.72) | Low*† | -0.26(-0.88, 0.36) | Low*† | 0.966 |
| TT vs VR-GT | 1 | 0.8 | -0.10(-0.85, 0.65) | Low‡ | -1.12(-2.6, 0.35) | Very low*‡ | -0.31(-0.98, 0.36) | Low‡ | 0.2253 |
| VR-GT vs CON | 1 | 0.32 | 1.36(0.24, 2.49) | Low*† | 0.70(-0.07, 1.47) | Very low*‡ | 0.91(0.27, 1.55) | Low*† | 0.3401 |
| AQE-BGT vs BGT-ECA | 0 | 0 |  |  | 0.59(-0.28, 1.46) | Very low*‡ | 0.59(-0.28, 1.46) | Very low*‡ |  |
| AQE-BGT vs BWS-TT | 0 | 0 |  |  | 1.33(0.49, 2.17) | Low‡ | 1.33(0.49, 2.17) | Low‡ |  |
| AQE-BGT vs BWS-TT-ECA | 0 | 0 |  |  | 0.76(-0.09, 1.61) | Very low*‡ | 0.76(-0.09, 1.61) | Very low*‡ |  |
| AQE-BGT vs DT-BGT | 0 | 0 |  |  | -0.16(-1.50, 1.17) | Low‡ | -0.16(-1.50, 1.17) | Low‡ |  |
| AQE-BGT vs EC-BGT | 0 | 0 |  |  | 0.28(-0.79, 1.35) | Very low*‡ | 0.28(-0.79, 1.35) | Very low*‡ |  |
| AQE-BGT vs RA-GT | 0 | 0 |  |  | 0.83(0.01, 1.65) | Very low*‡ | 0.83(0.01, 1.65) | Very low*‡ |  |
| AQE-BGT vs TT | 0 | 0 |  |  | 0.88(-0.19, 1.95) | Low‡ | 0.88(-0.19, 1.95) | Low‡ |  |
| AQE-BGT vs VR-GT | 0 | 0 |  |  | 0.57(-0.40, 1.54) | Low*† | 0.57(-0.40, 1.54) | Low*† |  |
| BGT vs DT-BGT | 0 | 0 |  |  | -1.26(-2.41, 0.11) | Very low*‡ | -1.26(-2.41, 0.11) | Very low*‡ |  |
| BGT vs TT | 0 | 0 |  |  | -0.22(-1.04, 0.60) | Very low*‡ | -0.22(-1.04, 0.60) | Very low*‡ |  |
| BGT vs VR-GT | 0 | 0 |  |  | -0.53(-1.23, 0.17) | Very low*‡ | -0.53(-1.23, 0.17) | Very low*‡ |  |
| BGT-ECA vs BWS-TT | 0 | 0 |  |  | 0.74(0.10, 1.39) | Low‡ | 0.74(0.10, 1.39) | Low‡ |  |
| BGT-ECA vs BWS-TT-ECA | 0 | 0 |  |  | 0.17(-0.49, 0.83) | Very low*‡ | 0.17(-0.49, 0.83) | Very low*‡ |  |
| BGT-ECA vs CON | 0 | 0 |  |  | 0.89(0.26, 1.52) | Low‡ | 0.89(0.26, 1.52) | Low‡ |  |
| BGT-ECA vs DT-BGT | 0 | 0 |  |  | -0.75(-1.98, 0.48) | Very low*‡ | -0.75(-1.98, 0.48) | Very low*‡ |  |
| BGT-ECA vs EC-BGT | 0 | 0 |  |  | -0.31(-1.23, 0.61) | Very low*‡ | -0.31(-1.23, 0.61) | Very low*‡ |  |
| BGT-ECA vs RA-GT | 0 | 0 |  |  | 0.24(-0.43, 0.91) | Very low*‡ | 0.24(-0.43, 0.91) | Very low*‡ |  |
| BGT-ECA vs TT | 0 | 0 |  |  | 0.29(-0.65, 1.22) | Very low*‡ | 0.29(-0.65, 1.22) | Very low*‡ |  |
| BGT-ECA vs VR-GT | 0 | 0 |  |  | -0.02(-0.85, 0.81) | Very low*‡ | -0.02(-0.85, 0.81) | Very low*‡ |  |
| BWS-TT vs DT-BGT | 0 | 0 |  |  | -1.50(-2.70, -0.30) | Very low*‡ | -1.50(-2.70, -0.30) | Very low*‡ |  |
| BWS-TT vs EC-BGT | 0 | 0 |  |  | -1.05(-1.95, -0.16) | Very low*‡ | -1.05(-1.95, -0.16) | Very low*‡ |  |
| BWS-TT vs RA-GT | 0 | 0 |  |  | -0.50(-1.07, 0.06) | Low‡ | -0.50(-1.07, 0.06) | Low‡ |  |
| BWS-TT vs TT | 0 | 0 |  |  | -0.45(-1.35, 0.44) | Low‡ | -0.45(-1.35, 0.44) | Low‡ |  |
| BWS-TT vs VR-GT | 0 | 0 |  |  | -0.76(-1.53, 0.00) | Low‡ | -0.76(-1.53, 0.00) | Low‡ |  |
| BWS-TT-ECA vs DT-BGT | 0 | 0 |  |  | -0.92(-2.13, 0.28) | Very low*‡ | -0.92(-2.13, 0.28) | Very low*‡ |  |
| BWS-TT-ECA vs EC-BGT | 0 | 0 |  |  | -0.48(-1.39, 0.43) | Very low*‡ | -0.48(-1.39, 0.43) | Very low*‡ |  |
| BWS-TT-ECA vs RA-GT | 0 | 0 |  |  | 0.07(-0.52, 0.65) | Very low*‡ | 0.07(-0.52, 0.65) | Very low*‡ |  |
| BWS-TT-ECA vs TT | 0 | 0 |  |  | 0.12(-0.79, 1.02) | Very low*‡ | 0.12(-0.79, 1.02) | Very low*‡ |  |
| BWS-TT-ECA vs VR-GT | 0 | 0 |  |  | -0.19(-0.97, 0.59) | Low*† | -0.19(-0.97, 0.59) | Low*† |  |
| DT-BGT vs CON | 0 | 0 |  |  | 1.64(0.50, 2.78) | Low‡ | 1.64(0.50, 2.78) | Low‡ |  |
| DT-BGT vs EC-BGT | 0 | 0 |  |  | 0.44(-0.58, 1.47) | Moderate† | 0.44(-0.58, 1.47) | Moderate† |  |
| DT-BGT vs RA-GT | 0 | 0 |  |  | 0.99(-0.15, 2.13) | Very low*‡ | 0.99(-0.15, 2.13) | Very low*‡ |  |
| DT-BGT vs VR-GT | 0 | 0 |  |  | 0.73(-0.31, 1.78) | Low‡ | 0.73(-0.31, 1.78) | Low‡ |  |
| EC-BGT vs CON | 0 | 0 |  |  | 1.64(0.50, 2.78) | Very low*‡ | 1.64(0.50, 2.78) | Very low*‡ |  |
| EC-BGT vs RA-GT | 0 | 0 |  |  | 0.55(-0.30, 1.39) | Very low*‡ | 0.55(-0.30, 1.39) | Very low*‡ |  |
| EC-BGT vs VR-GT | 0 | 0 |  |  | 0.29(-0.52, 1.09) | Very low*‡ | 0.29(-0.52, 1.09) | Very low*‡ |  |
| RA-GT vs TT | 0 | 0 |  |  | 0.05(-0.76, 0.86) | Very low*‡ | 0.05(-0.76, 0.86) | Very low*‡ |  |
| TT vs CON | 0 | 0 |  |  | 0.60(-0.21, 1.41) | Low‡ | 0.60(-0.21, 1.41) | Low‡ |  |

k = Number of studies providing direct evidence, prop = proportion of direct evidence, NMA (SMD) = estimated treatment effect (SMD) in network meta-analysis, direct (SMD) = estimated treatment effect (SMD) derived from direct evidence, indirect (SMD) = estimated treatment effect (SMD) derived from indirect evidence, p = p-value of test for disagreement (difference between direct and indirect evidence)

Reasons for downgrading: †Imprecision (1 downgrade), ‡Severe imprecision (2 downgrades), *risk of bias (1 downgrade)

The p-value indicated that the findings between direct and indirect comparisons was not statistically significant and therefore no comparisons have been downgraded based on inconsistency

**10.4 Details of GRADE assessment for all pairwise comparisons within the proactive balance network**

| **Comparison** | **k** | **prop** | **Direct SMD [95% CI]** | **Quality of evidence** | **Indirect SMD [95% CI]** | **Quality of evidence** | **NMA SMD [95% CI]** | **Quality of evidence** | **P-value** |
| --- | --- | --- | --- | --- | --- | --- | --- | --- | --- |
| BGT vs BGT-ECA | 4 | 0.68 | -0.31(-0.89, 0.26) | Very low*‡ | 0.56(-0.27, 1.40) | Very low*‡ | -0.03(-0.51, 0.44) | Very low*‡ | 0.0905 |
| BGT vs BWS-TT | 1 | 1 | 0.49(-0.51, 1.50) | Low‡ |  |  | 0.49(-0.51, 1.50) | Low‡ |  |
| BGT vs CON | 2 | 0.36 | 0.59('-0.21, 1.38) | Very low*‡ | 0.38(-0.22, 0.98) | Very low*‡ | 0.45(-0.02, 0.93) | Very low*‡ | 0.6801 |
| BGT vs RA-GT | 2 | 0.41 | 0.06(-0.78, 0.91) | Very low*‡ | 0.01(-0.70, 0.72) | Very low*‡ | 0.03(-0.51, 0.57) | Very low*‡ | 0.9210 |
| BGT vs VR-GT | 1 | 0.48 | 0.04(-0.90, 0.98) | Low‡ | -0.98(-1.89, -0.07) | Very low*‡ | -0.48(-1.14, 0.17) | Low‡ | 0.1274 |
| BGT-ECA vs CON | 2 | 0.51 | -0.00(-0.71, 0.71) | Very low*‡ | 0.99(0.26, 1.71) | Very low*‡ | 0.48(-0.02, 0.99) | Very low*‡ | 0.0563 |
| BGT-ECA vs TT-ECA | 1 | 0.4 | -0.40(-1.59, 0.79) | Very low*‡ | -0.65(-1.63, 0.32) | Very low*‡ | -0.55(-1.31, 0.20) | Very low*‡ | 0.7450 |
| BWS-TT vs BWS-TT-ECA | 1 | 1 | -1.61(-2.75, -0.47) | Moderate† |  |  | -1.61(-2.75, -0.47) | Moderate† |  |
| DT-BGT vs TT | 1 | 1 | 0.28(-0.78, 1.34) | Low‡ |  |  | 0.28(-0.78, 1.34) | Low‡ |  |
| EC-BGT vs CON | 1 | 1 | 0.28(-0.97, 1.53) | Low‡ |  |  | 0.28(-0.97, 1.53) | Low‡ |  |
| RA-GT vs CON | 3 | 0.62 | 0.37(-0.23, 0.96) | Very low*‡ | 0.51(-0.24, 1.26) | Low‡ | 0.42(-0.04, 0.89) | Low‡ | 0.7620 |
| RA-GT vs RA-GT-ECA | 1 | 0.46 | -0.03(-1.20, 1.14) | Low‡ | -0.17(-1.26, 0.91) | Very low*‡ | -0.11(-0.90, 0.69) | Low‡ | 0.8601 |
| RA-GT vs TT | 2 | 0.37 | 1.11(0.00, 2.21) | Low*† | 0.81(-0.04, 1.66) | Very low*‡ | 0.92(0.25, 1.60) | Low*† | 0.6796 |
| RA-GT-ECA vs CON | 1 | 0.53 | 0.85(-0.24, 1.94) | Very low*‡ | 0.17(-0.99, 1.32) | Very low*‡ | 0.53(-0.26, 1.32) | Very low*‡ | 0.3980 |
| RA-GT-ECA vs VR-GT | 1 | 0.6 | -0.66(-1.78, 0.46) | Very low*‡ | -0.03(-1.41, 1.35) | Very low*‡ | -0.41(-1.28, 0.46) | Very low*‡ | 0.4875 |
| TT vs CON | 1 | 0.3 | 0.23(-0.94, 1.40) | Low‡ | -0.82(-1.59, -0.05) | Very low*‡ | -0.50(-1.14, 0.14) | Low‡ | 0.1416 |
| TT vs TT-ECA | 3 | 0.83 | -1.50(-2.17, -0.82) | Low*† | 1.74(-3.25, -0.24) | Low‡ | -1.54(-2.16, -0.92) | Low‡ | 0.7676 |
| TT vs VR-GT | 2 | 0.53 | -1.59(-2.55, -0.64) | Low*† | -1.26(-2.28, -0.24) | Low‡ | -1.44(-2.13, -0.74) | Low‡ | 0.6399 |
| TT-ECA vs CON | 1 | 0.36 | 1.17(-0.04, 2.38) | Low‡ | 0.96(0.06, 1.87) | Very low*‡ | 1.04(0.31, 1.76) | Low‡ | 0.7875 |
| VR-GT vs CON | 1 | 0.34 | 1.51(0.38, 2.64) | Low*† | 0.64(-0.16, 1.45) | Very low*‡ | 0.94(0.28, 1.59) | Low*† | 0.2215 |
| BGT vs BWS-TT-ECA | 0 | 0 |  |  | -1.11(-2.63, 0.41) | Low‡ | -1.11(-2.63, 0.41) | Low‡ |  |
| BGT vs DT-BGT | 0 | 0 |  |  | 0.67(-0.60, 1.94) | Very low*‡ | 0.67(-0.60, 1.94) | Very low*‡ |  |
| BGT vs EC-BGT | 0 | 0 |  |  | 0.17(-1.16, 1.51) | Very low*‡ | 0.17(-1.16, 1.51) | Very low*‡ |  |
| BGT vs RA-GT-ECA | 0 | 0 |  |  | -0.08(-0.93, 0.78) | Very low*‡ | -0.08(-0.93, 0.78) | Very low*‡ |  |
| BGT vs TT | 0 | 0 |  |  | 0.95(0.26, 1.65) | Very low*‡ | 0.95(0.26, 1.65) | Very low*‡ |  |
| BGT vs TT-ECA | 0 | 0 |  |  | -0.58(-1.36, 0.19) | Very low*‡ | -0.58(-1.36, 0.19) | Very low*‡ |  |
| BGT-ECA vs BWS-TT | 0 | 0 |  |  | 0.53(-0.59, 1.64) | Very low*‡ | 0.53(-0.59, 1.64) | Very low*‡ |  |
| BGT-ECA vs BWS-TT-ECA | 0 | 0 |  |  | -1.08(-2.67, 0.51) | Very low*‡ | -1.08(-2.67, 0.51) | Very low*‡ |  |
| BGT-ECA vs DT-BGT | 0 | 0 |  |  | 0.70(-0.58, 1.99) | Very low*‡ | 0.70(-0.58, 1.99) | Very low*‡ |  |
| BGT-ECA vs EC-BGT | 0 | 0 |  |  | 0.21(-1.14, 1.56) | Very low*‡ | 0.21(-1.14, 1.56) | Very low*‡ |  |
| BGT-ECA vs RA-GT | 0 | 0 |  |  | 0.06(-0.55, 0.68) | Very low*‡ | 0.06(-0.55, 0.68) | Very low*‡ |  |
| BGT-ECA vs RA-GT-ECA | 0 | 0 |  |  | -0.04(-0.94, 0.85) | Very low*‡ | -0.04(-0.94, 0.85) | Very low*‡ |  |
| BGT-ECA vs TT | 0 | 0 |  |  | 0.99(0.26, 1.71) | Very low*‡ | 0.99(0.26, 1.71) | Very low*‡ |  |
| BGT-ECA vs VR-GT | 0 | 0 |  |  | -0.45(-1.18, 0.28) | Very low*‡ | -0.45(-1.18, 0.28) | Very low*‡ |  |
| BWS-TT vs CON | 0 | 0 |  |  | -0.04(-1.15, 1.07) | Very low*‡ | -0.04(-1.15, 1.07) | Very low*‡ |  |
| BWS-TT vs DT-BGT | 0 | 0 |  |  | 0.18(-1.44, 1.80) | Very low*‡ | 0.18(-1.44, 1.80) | Very low*‡ |  |
| BWS-TT vs EC-BGT | 0 | 0 |  |  | -0.32(-1.99, 1.35) | Very low*‡ | -0.32(-1.99, 1.35) | Very low*‡ |  |
| BWS-TT vs RA-GT | 0 | 0 |  |  | -0.46(-1.60, 0.68) | Very low*‡ | -0.46(-1.60, 0.68) | Very low*‡ |  |
| BWS-TT vs RA-GT-ECA | 0 | 0 |  |  | -0.57(-1.89, 0.75) | Very low*‡ | -0.57(-1.89, 0.75) | Very low*‡ |  |
| BWS-TT vs TT | 0 | 0 |  |  | 0.46(-0.76, 1.68) | Very low*‡ | 0.46(-0.76, 1.68) | Very low*‡ |  |
| BWS-TT vs TT-ECA | 0 | 0 |  |  | -1.08(-2.34, 0.19) | Very low*‡ | -1.08(-2.34, 0.19) | Very low*‡ |  |
| BWS-TT vs VR-GT | 0 | 0 |  |  | -0.98(-2.18, 0.22) | Low‡ | -0.98(-2.18, 0.22) | Low‡ |  |
| BWS-TT-ECA vs CON | 0 | 0 |  |  | 1.57(-0.03, 3.16) | Very low*‡ | 1.57(-0.03, 3.16) | Very low*‡ |  |
| BWS-TT-ECA vs DT-BGT | 0 | 0 |  |  | 1.78(-0.20, 3.76) | Very low*‡ | 1.78(-0.20, 3.76) | Very low*‡ |  |
| BWS-TT-ECA vs EC-BGT | 0 | 0 |  |  | 1.29(-0.74, 3.31) | Very low*‡ | 1.29(-0.74, 3.31) | Very low*‡ |  |
| BWS-TT-ECA vs RA-GT | 0 | 0 |  |  | 1.14(-0.47, 2.76) | Very low*‡ | 1.14(-0.47, 2.76) | Very low*‡ |  |
| BWS-TT-ECA vs RA-GT-ECA | 0 | 0 |  |  | 1.04(-0.71, 2.78) | Very low*‡ | 1.04(-0.71, 2.78) | Very low*‡ |  |
| BWS-TT-ECA vs TT | 0 | 0 |  |  | 2.07(0.40, 3.74) | Very low*‡ | 2.07(0.40, 3.74) | Very low*‡ |  |
| BWS-TT-ECA vs TT-ECA | 0 | 0 |  |  | 0.53(-1.17, 2.23) | Very low*‡ | 0.53(-1.17, 2.23) | Very low*‡ |  |
| BWS-TT-ECA vs VR-GT | 0 | 0 |  |  | 0.63(-1.03, 2.28) | Low‡ | 0.63(-1.03, 2.28) | Low‡ |  |
| DT-BGT vs CON | 0 | 0 |  |  | -0.22(-1.46, 1.02) | Low‡ | -0.22(-1.46, 1.02) | Low‡ |  |
| DT-BGT vs EC-BGT | 0 | 0 |  |  | -0.50(-2.26, 1.26) | Low‡ | -0.50(-2.26, 1.26) | Low‡ |  |
| DT-BGT vs RA-GT | 0 | 0 |  |  | -0.64(-1.90, 0.62) | Low‡ | -0.64(-1.90, 0.62) | Low‡ |  |
| DT-BGT vs RA-GT-ECA | 0 | 0 |  |  | -0.75(-2.16, 0.66) | Low‡ | -0.75(-2.16, 0.66) | Low‡ |  |
| DT-BGT vs TT-ECA | 0 | 0 |  |  | -1.26(-2.48, -0.03) | Low‡ | -1.26(-2.48, -0.03) | Low‡ |  |
| DT-BGT vs VR-GT | 0 | 0 |  |  | -1.16(-2.43, 0.11) | Low‡ | -1.16(-2.43, 0.11) | Low‡ |  |
| EC-BGT vs RA-GT | 0 | 0 |  |  | -0.14(-1.48, 1.19) | Very low*‡ | -0.14(-1.48, 1.19) | Very low*‡ |  |
| EC-BGT vs RA-GT-ECA | 0 | 0 |  |  | -0.25(-1.73, 1.23) | Very low*‡ | -0.25(-1.73, 1.23) | Very low*‡ |  |
| EC-BGT vs TT | 0 | 0 |  |  | 0.78(-0.63, 2.19) | Low‡ | 0.78(-0.63, 2.19) | Low‡ |  |
| EC-BGT vs TT-ECA | 0 | 0 |  |  | -0.76(-2.20, 0.69) | Low‡ | -0.76(-2.20, 0.69) | Low‡ |  |
| EC-BGT vs VR-GT | 0 | 0 |  |  | -0.66(-2.07, 0.75) | Low‡ | -0.66(-2.07, 0.75) | Low‡ |  |
| RA-GT vs TT-ECA | 0 | 0 |  |  | -0.62(-1.39, 0.16) | Low*† | -0.62(-1.39, 0.16) | Low*† |  |
| RA-GT vs VR-GT | 0 | 0 |  |  | -0.51(-1.22, 0.19) | Very low*‡ | -0.51(-1.22, 0.19) | Very low*‡ |  |
| RA-GT-ECA vs TT | 0 | 0 |  |  | 1.03(0.10, 1.96) | Very low*‡ | 1.03(0.10, 1.96) | Very low*‡ |  |
| RA-GT-ECA vs TT-ECA | 0 | 0 |  |  | -0.51(-1.52, 0.50) | Very low*‡ | -0.51(-1.52, 0.50) | Very low*‡ |  |
| TT-ECA vs VR-GT | 0 | 0 |  |  | 0.10(-0.73, 0.93) | Low‡ | 0.10(-0.73, 0.93) | Low‡ |  |

k = Number of studies providing direct evidence, prop = proportion of direct evidence, NMA (SMD) = estimated treatment effect (SMD) in network meta-analysis, direct (SMD) = estimated treatment effect (SMD) derived from direct evidence, indirect (SMD) = estimated treatment effect (SMD) derived from indirect evidence, p = p-value of test for disagreement (difference between direct and indirect evidence)

Reasons for downgrading: †Imprecision (1 downgrade), ‡Severe imprecision (2 downgrades), *risk of bias (1 downgrade)

The p-value indicated that the findings between direct and indirect comparisons was not statistically significant and therefore no comparisons have been downgraded based on inconsistency

**Appendix 11: Network meta-analysis funnel plots**

**
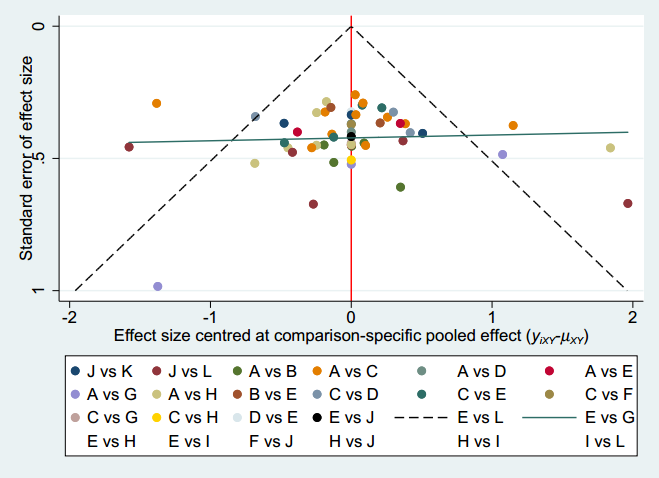
**

**Figure. S14 Funnel plot based on the balance test batteries.** A: BGT; B: BGT-ECA; C: BWS-TT; D: BWS-TT-ECA; E: CON; F: DT-BGT; G: EC-BGT; H: RA-GT; I:RA-GT-ECA; J: TT; K: TT-ECA; L: VR-GT.

**
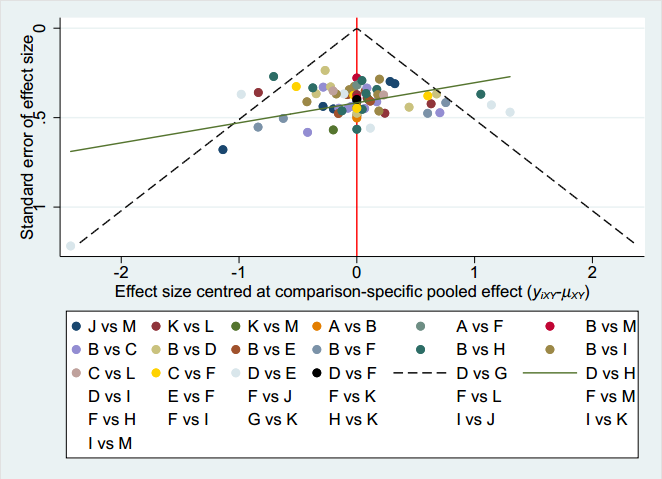
**

**Figure. S15 Funnel plot based on the dynamic steady-state balance.** A: AQE-BGT; B: BGT; C: BGT-ECA; D: BWS-TT; E: BWS-TT-ECA; F: CON; G: DT-BGT; H: EC-BGT; I: RA-GT; J: RA-GT-ECA; K: TT; L: TT-ECA; M: VR-GT.

**
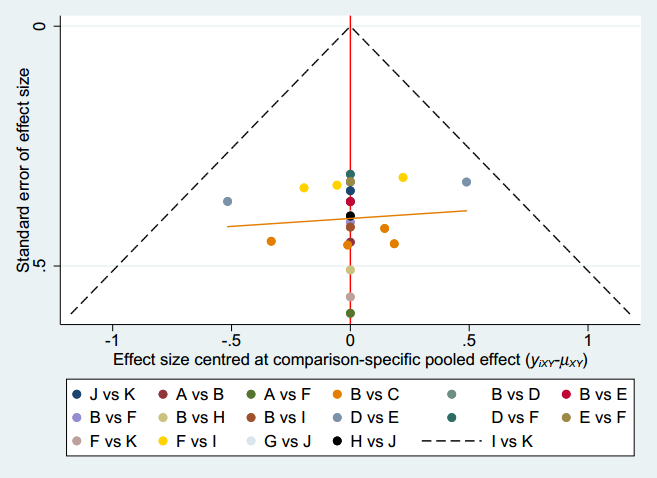
**

**Figure. S16 Funnel plot based on the static steady-state balance.** A: AQE-BGT; B: BGT; C: BGT-ECA; D: BWS-TT; E: BWS-TT-ECA; F: CON; G: DT-BGT; H: EC-BGT; I: RA-GT; J: TT; K: VR-GT.

**
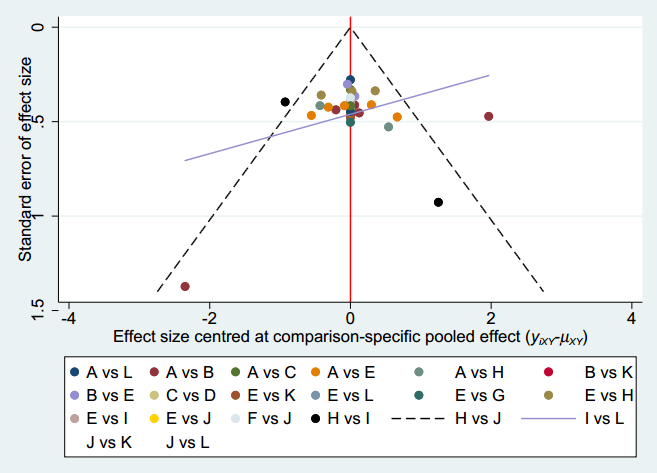
**

**Figure. S17 Funnel plot based on the Proactive balance.** A: BGT; B: BGT-ECA; C: BWS-TT; D: BWS-TT-ECA; E: CON; F: DT-BGT; G: EC-BGT; H: RA-GT; I: RA-GT-ECA; J: TT; K: TT-ECA; L: VR-GT.

**List of included literature**

1. Rose DK, DeMark L, Fox EJ, Clark DJ, Wludyka P. A Backward Walking Training Program to Improve Balance and Mobility in Acute Stroke: A Pilot Randomized Controlled Trial. J Neurol Phys Ther. 2018;42(1):12-21. doi:10.1097/npt.0000000000000210

2. Pignolo L, Basta G, Carozzo S, et al. A body-weight-supported visual feedback system for gait recovering in stroke patients: A randomized controlled study. Gait Posture. 2020;82:287-293. doi:10.1016/j.gaitpost.2020.09.020

3. Belas Dos Santos M, Barros de Oliveira C, Dos Santos A, Garabello Pires C, Dylewski V, Arida RM. A Comparative Study of Conventional Physiotherapy versus Robot-Assisted Gait Training Associated to Physiotherapy in Individuals with Ataxia after Stroke. Behav Neurol. 2018;2018:2892065. doi:10.1155/2018/2892065

4. Middleton A, Merlo-Rains A, Peters DM, et al. Body weight-supported treadmill training is no better than overground training for individuals with chronic stroke: a randomized controlled trial. Top Stroke Rehabil. 2014;21(6):462-476. doi:10.1310/tsr2106-462

5. Globas C, Becker C, Cerny J, et al. Chronic stroke survivors benefit from high-intensity aerobic treadmill exercise: a randomized control trial. Neurorehabil Neural Repair. 2012;26(1):85-95. doi:10.1177/1545968311418675

6. Park J, Park SY, Kim YW, Woo Y. Comparison between treadmill training with rhythmic auditory stimulation and ground walking with rhythmic auditory stimulation on gait ability in chronic stroke patients: A pilot study. NeuroRehabilitation. 2015;37(2):193-202. doi:10.3233/nre-151252

7. Kim NH, Park HY, Son JK, Moon Y, Lee JH, Cha YJ. Comparison of underwater gait training and overground gait training for improving the walking and balancing ability of patients with severe hemiplegic stroke: A randomized controlled pilot trial. Gait Posture. 2020;80:124-129. doi:10.1016/j.gaitpost.2020.05.022

8. Mackay-Lyons M, McDonald A, Matheson J, Eskes G, Klus MA. Dual effects of body-weight supported treadmill training on cardiovascular fitness and walking ability early after stroke: a randomized controlled trial. Neurorehabil Neural Repair. 2013;27(7):644-653. doi:10.1177/1545968313484809

9. Wright A, Stone K, Martinelli L, et al. Effect of combined home-based, overground robotic-assisted gait training and usual physiotherapy on clinical functional outcomes in people with chronic stroke: A randomized controlled trial. Clin Rehabil. 2021;35(6):882-893. doi:10.1177/0269215520984133

10. Cho KH, Lee WH. Effect of treadmill training based real-world video recording on balance and gait in chronic stroke patients: a randomized controlled trial. Gait Posture. 2014;39(1):523-528. doi:10.1016/j.gaitpost.2013.09.003

11. de Rooij IJM, van de Port IGL, Punt M, et al. Effect of Virtual Reality Gait Training on Participation in Survivors of Subacute Stroke: A Randomized Controlled Trial. Phys Ther. 2021;101(5). doi:10.1093/ptj/pzab051

12. Park HJ, Oh DW, Kim SY, Choi JD. Effectiveness of community-based ambulation training for walking function of post-stroke hemiparesis: a randomized controlled pilot trial. Clin Rehabil. 2011;25(5):451-459. doi:10.1177/0269215510389200

13. Tong RK, Ng MF, Li LS. Effectiveness of gait training using an electromechanical gait trainer, with and without functional electric stimulation, in subacute stroke: a randomized controlled trial. Arch Phys Med Rehabil. 2006;87(10):1298-1304. doi:10.1016/j.apmr.2006.06.016

14. Kim N, Park Y, Lee BH. Effects of community-based virtual reality treadmill training on balance ability in patients with chronic stroke. Journal of physical therapy science. 2015;27(3):655-658. doi:10.1589/jpts.27.655

15. Nam YG, Lee JW, Park JW, et al. Effects of Electromechanical Exoskeleton-Assisted Gait Training on Walking Ability of Stroke Patients: A Randomized Controlled Trial. Arch Phys Med Rehabil. 2019;100(1):26-31. doi:10.1016/j.apmr.2018.06.020

16. Rojek A, Mika A, Oleksy Ł, Stolarczyk A, Kielnar R. Effects of Exoskeleton Gait Training on Balance, Load Distribution, and Functional Status in Stroke: A Randomized Controlled Trial. Front Neurol. 2019;10:1344. doi:10.3389/fneur.2019.01344

17. Ogino T, Kanata Y, Uegaki R, et al. Effects of gait exercise assist robot (GEAR) on subjects with chronic stroke: A randomized controlled pilot trial. Journal of stroke and cerebrovascular diseases : the official journal of National Stroke Association. 2020;29(8):104886. doi:10.1016/j.jstrokecerebrovasdis.2020.104886

18. Cha YJ, Kim JD, Choi YR, Kim NH, Son SM. Effects of gait training with auditory feedback on walking and balancing ability in adults after hemiplegic stroke: a preliminary, randomized, controlled study. Int J Rehabil Res. 2018;41(3):239-243. doi:10.1097/mrr.0000000000000295

19. Takami A, Wakayama S. Effects of partial body weight support while training acute stroke patients to walk backwards on a treadmill-a controlled clinical trial using randomized allocation. Journal of physical therapy science. 2010;22(2):177-187.

20. Kim KH, Lee KB, Bae YH, Fong SSM, Lee SM. Effects of progressive backward body weight suppoted treadmill training on gait ability in chronic stroke patients: A randomized controlled trial. Technology and health care : official journal of the European Society for Engineering and Medicine. 2017;25(5):867-876. doi:10.3233/thc-160720

21. Kim J, Kim DY, Chun MH, et al. Effects of robot-(Morning Walk(®)) assisted gait training for patients after stroke: a randomized controlled trial. Clin Rehabil. 2019;33(3):516-523. doi:10.1177/0269215518806563

22. Bae YH, Ko YJ, Chang WH, et al. Effects of Robot-assisted Gait Training Combined with Functional Electrical Stimulation on Recovery of Locomotor Mobility in Chronic Stroke Patients: A Randomized Controlled Trial. Journal of physical therapy science. 2014;26(12):1949-1953. doi:10.1589/jpts.26.1949

23. Bang DH, Shin WS. Effects of robot-assisted gait training on spatiotemporal gait parameters and balance in patients with chronic stroke: A randomized controlled pilot trial. NeuroRehabilitation. 2016;38(4):343-349. doi:10.3233/nre-161325

24. Kang HK, Kim Y, Chung Y, Hwang S. Effects of treadmill training with optic flow on balance and gait in individuals following stroke: randomized controlled trials. Clin Rehabil. 2012;26(3):246-255. doi:10.1177/0269215511419383

25. Kim YW, Moon SJ. Effects of treadmill training with the eyes closed on gait and balance ability of chronic stroke patients. Journal of physical therapy science. 2015;27(9):2935-2938. doi:10.1589/jpts.27.2935

26. Huang WY, Tuan SH, Li MH, Hsu PT. Efficacy of a novel walking assist device with auxiliary laser illuminator in stroke Patients~ a randomized control trial. J Formos Med Assoc. 2022;121(3):592-603. doi:10.1016/j.jfma.2021.06.019

27. Huang WY, Li MH, Lee CH, Tuan SH, Sun SF, Liou IH. Efficacy of lateral stair walking training in patients with chronic stroke: A pilot randomized controlled study. Gait Posture. 2021;88:10-15. doi:10.1016/j.gaitpost.2021.04.026

28. van Bloemendaal M, Bus SA, Nollet F, Geurts ACH, Beelen A. Feasibility and Preliminary Efficacy of Gait Training Assisted by Multichannel Functional Electrical Stimulation in Early Stroke Rehabilitation: A Pilot Randomized Controlled Trial. Neurorehabil Neural Repair. 2021;35(2):131-144. doi:10.1177/1545968320981942

29. Nam YG, Park JW, Lee HJ, et al. Further effects of electromechanically assisted gait trainer (Exowalk®) in patients with chronic stroke: A randomized controlled trial. J Rehabil Med. 2020;52(9):jrm00097. doi:10.2340/16501977-2723

30. Yen CL, Wang RY, Liao KK, Huang CC, Yang YR. Gait training induced change in corticomotor excitability in patients with chronic stroke. Neurorehabil Neural Repair. 2008;22(1):22-30. doi:10.1177/1545968307301875

31. Dragin AS, Konstantinović LM, Veg A, Schwirtlich LB. Gait training of poststroke patients assisted by the Walkaround (body postural support). Int J Rehabil Res. 2014;37(1):22-28. doi:10.1097/MRR.0b013e328363ba30

32. Stein J, Bishop L, Stein DJ, Wong CK. Gait training with a robotic leg brace after stroke: a randomized controlled pilot study. Am J Phys Med Rehabil. 2014;93(11):987-994. doi:10.1097/phm.0000000000000119

33. Jung KS, Bang H, In TS, Cho HY. Gait training with auditory feedback improves trunk control, muscle activation and dynamic balance in patients with hemiparetic stroke: A randomized controlled pilot study. J Back Musculoskelet Rehabil. 2020;33(1):1-6. doi:10.3233/bmr-170852

34. Lee S, Lee K, Song C. Gait Training with Bilateral Rhythmic Auditory Stimulation in Stroke Patients: A Randomized Controlled Trial. Brain Sci. 2018;8(9). doi:10.3390/brainsci8090164

35. Lloréns R, Gil-Gómez JA, Alcañiz M, Colomer C, Noé E. Improvement in balance using a virtual reality-based stepping exercise: a randomized controlled trial involving individuals with chronic stroke. Clin Rehabil. 2015;29(3):261-268. doi:10.1177/0269215514543333

36. Cha Y, Kim Y, Hwang S, Chung Y. Intensive gait training with rhythmic auditory stimulation in individuals with chronic hemiparetic stroke: a pilot randomized controlled study. NeuroRehabilitation. 2014;35(4):681-688. doi:10.3233/nre-141182

37. Broderick P, Horgan F, Blake C, Ehrensberger M, Simpson D, Monaghan K. Mirror therapy and treadmill training for patients with chronic stroke: a pilot randomized controlled trial. Top Stroke Rehabil. 2019;26(3):163-172. doi:10.1080/10749357.2018.1556504

38. Bizovičar N, Matjačić Z, Stanonik I, Goljar N. Overground gait training using a motorized assistive device in patients with severe disabilities after stroke. Int J Rehabil Res. 2017;40(1):46-52. doi:10.1097/mrr.0000000000000199

39. Westlake KP, Patten C. Pilot study of Lokomat versus manual-assisted treadmill training for locomotor recovery post-stroke. J Neuroeng Rehabil. 2009;6:18. doi:10.1186/1743-0003-6-18

40. Sheikh M, Azarpazhooh MR, Hosseini HA. Randomized comparison trial of gait training with and without compelled weight-shift therapy in individuals with chronic stroke. Clin Rehabil. 2016;30(11):1088-1096. doi:10.1177/0269215515611467

41. Yeung LF, Ockenfeld C, Pang MK, et al. Randomized controlled trial of robot-assisted gait training with dorsiflexion assistance on chronic stroke patients wearing ankle-foot-orthosis. J Neuroeng Rehabil. 2018;15(1):51. doi:10.1186/s12984-018-0394-7

42. Yun N, Joo MC, Kim SC, Kim MS. Robot-assisted gait training effectively improved lateropulsion in subacute stroke patients: a single-blinded randomized controlled trial. Eur J Phys Rehabil Med. 2018;54(6):827-836. doi:10.23736/s1973-9087.18.05077-3

43. Fisher S, Lucas L, Thrasher TA. Robot-assisted gait training for patients with hemiparesis due to stroke. Top Stroke Rehabil. 2011;18(3):269-276. doi:10.1310/tsr1803-269

44. Han EY, Im SH, Kim BR, Seo MJ, Kim MO. Robot-assisted gait training improves brachial-ankle pulse wave velocity and peak aerobic capacity in subacute stroke patients with totally dependent ambulation: Randomized controlled trial. Medicine (Baltimore). 2016;95(41):e5078. doi:10.1097/md.0000000000005078

45. Jayaraman A, O'Brien MK, Madhavan S, et al. Stride management assist exoskeleton vs functional gait training in stroke: A randomized trial. Neurology. 2019;92(3):e263-e273. doi:10.1212/wnl.0000000000006782

46. Mao YR, Lo WL, Lin Q, et al. The Effect of Body Weight Support Treadmill Training on Gait Recovery, Proximal Lower Limb Motor Pattern, and Balance in Patients with Subacute Stroke. Biomed Res Int. 2015;2015:175719. doi:10.1155/2015/175719

47. Song KJ, Chun MH, Lee J, Lee C. The effect of robot-assisted gait training on cortical activation in stroke patients: A functional near-infrared spectroscopy study. NeuroRehabilitation. 2021;49(1):65-73. doi:10.3233/nre-210034

48. Park SW, Lee KJ, Shin DC, Shin SH, Lee MM, Song CH. The effect of underwater gait training on balance ability of stroke patients. Journal of physical therapy science. 2014;26(6):899-903. doi:10.1589/jpts.26.899

49. Chang KW, Lin CM, Yen CW, Yang CC, Tanaka T, Guo LY. The Effect of Walking Backward on a Treadmill on Balance, Speed of Walking and Cardiopulmonary Fitness for Patients with Chronic Stroke: A Pilot Study. Int J Environ Res Public Health. 2021;18(5). doi:10.3390/ijerph18052376

50. Stolz R, Nayyar R, Louie J, Bower KJ, Paul SK, Ng L. The effectiveness of a novel cable-driven gait trainer (Robowalk) combined with conventional physiotherapy compared to conventional physiotherapy alone following stroke: a randomised controlled trial. Int J Rehabil Res. 2019;42(4):377-384. doi:10.1097/mrr.0000000000000375

51. Peurala SH, Tarkka IM, Pitkänen K, Sivenius J. The effectiveness of body weight-supported gait training and floor walking in patients with chronic stroke. Arch Phys Med Rehabil. 2005;86(8):1557-1564. doi:10.1016/j.apmr.2005.02.005

52. Lee HJ, Cho KH, Lee WH. The effects of body weight support treadmill training with power-assisted functional electrical stimulation on functional movement and gait in stroke patients. Am J Phys Med Rehabil. 2013;92(12):1051-1059. doi:10.1097/phm.0000000000000040

53. Park J, Chung Y. The effects of robot-assisted gait training using virtual reality and auditory stimulation on balance and gait abilities in persons with stroke. NeuroRehabilitation. 2018;43(2):227-235. doi:10.3233/nre-172415

54. Jeong YG, Koo JW. The effects of treadmill walking combined with obstacle-crossing on walking ability in ambulatory patients after stroke: a pilot randomized controlled trial. Top Stroke Rehabil. 2016;23(6):406-412. doi:10.1080/10749357.2016.1168592

55. In T, Jin Y, Jung K, Cho HY. Treadmill training with Thera-Band improves motor function, gait and balance in stroke patients. NeuroRehabilitation. 2017;40(1):109-114. doi:10.3233/nre-161395

56. Hwang DY, Lee HJ, Lee GC, Lee SM. Treadmill training with tilt sensor functional electrical stimulation for improving balance, gait, and muscle architecture of tibialis anterior of survivors with chronic stroke: A randomized controlled trial. Technology and health care : official journal of the European Society for Engineering and Medicine. 2015;23(4):443-452. doi:10.3233/thc-150903

57. Chen IH, Yang YR, Chan RC, Wang RY. Turning-based treadmill training improves turning performance and gait symmetry after stroke. Neurorehabil Neural Repair. 2014;28(1):45-55. doi:10.1177/1545968313497102

58. Cho KH, Lee WH. Virtual walking training program using a real-world video recording for patients with chronic stroke: a pilot study. Am J Phys Med Rehabil. 2013;92(5):371-380; quiz 380-372, 458. doi:10.1097/PHM.0b013e31828cd5d3

59. Choi YH, Kim JD, Lee JH, Cha YJ. Walking and balance ability gain from two types of gait intervention in adult patients with chronic hemiplegic stroke: A pilot study. Assist Technol. 2019;31(2):112-115. doi:10.1080/10400435.2017.1387616

60. Graham SA, Roth EJ, Brown DA. Walking and balance outcomes for stroke survivors: a randomized clinical trial comparing body-weight-supported treadmill training with versus without challenging mobility skills. J Neuroeng Rehabil. 2018;15(1):92. doi:10.1186/s12984-018-0442-3

61. Nilsson L, Carlsson J, Danielsson A, et al. Walking training of patients with hemiparesis at an early stage after stroke: a comparison of walking training on a treadmill with body weight support and walking training on the ground. Clin Rehabil. 2001;15(5):515-527. doi:10.1191/026921501680425234

62. Elsner B, Schöler A, Kon T, Mehrholz J. Walking with rhythmic auditory stimulation in chronic patients after stroke: A pilot randomized controlled trial. Physiother Res Int. 2020;25(1):e1800. doi:10.1002/pri.1800

63. Yang YR, Tsai MP, Chuang TY, Sung WH, Wang RY. Virtual reality-based training improves community ambulation in individuals with stroke: a randomized controlled trial. Gait Posture. 2008;28(2):201-206. doi:10.1016/j.gaitpost.2007.11.007

64. Forrester LW, Roy A, Hafer-Macko C, Krebs HI, Macko RF. Task-specific ankle robotics gait training after stroke: a randomized pilot study. J Neuroeng Rehabil. 2016;13(1):51. doi:10.1186/s12984-016-0158-1

65. Inoue S, Otaka Y, Kumagai M, Sugasawa M, Mori N, Kondo K. Effects of Balance Exercise Assist Robot training for patients with hemiparetic stroke: a randomized controlled trial. J Neuroeng Rehabil. 2022;19(1):12. doi:10.1186/s12984-022-00989-6

66. Choi W. Effects of Robot-Assisted Gait Training with Body Weight Support on Gait and Balance in Stroke Patients. Int J Environ Res Public Health. 2022;19(10). doi:10.3390/ijerph19105814
